# Targeting m^6^A writer METTL3 with engineered nanovesicles reduces neuroinflammation in vitro and in vivo

**DOI:** 10.1038/s41467-026-75862-4

**Published:** 2026-08-08

**Authors:** Liangfu Xu, Yuanwei Pan, Guanjun Li, Peng She, Qian-Fang Meng, Zhigang Liu, Lang Rao

**Affiliations:** 1 Cancer Center, Dongguan Key Laboratory of Precision Diagnosis and Treatment for Tumors, The Tenth Affiliated Hospital, Southern Medical University (Dongguan People’s Hospital), Dongguan, China; 2 Shenzhen School of Clinical Medicine, Southern Medical University, Shenzhen, China; 3 Institute of Chemical Biology, Shenzhen Bay Laboratory, Shenzhen, China; 4 Department of Orthopedics, The Seventh Affiliated Hospital of Sun Yat-Sen University, Shenzhen, China

**Keywords:** Drug delivery, Tumour immunology

## Abstract

Epigenetic editing, particularly N^6^-methyladenosine (m^6^A) modification, represents a promising therapeutic strategy by silencing genes without altering DNA sequence. However, in vivo epigenetic intervention of neuroinflammation remains challenging and has rarely been explored. Here we developed a hybrid epigenetic nanomodulator, siMETTL3-hNVs, by integrating natural microglia-derived nanovesicles (NVs) with synthetic liposomes pre-loading small interfering RNA targeting the m^6^A writer methyltransferase-like 3 (METTL3). Natural NVs enabled siMETTL3-hNVs to achieve inflamed-brain delivery through CCR2-CCL2 chemotaxis and caveolae-mediated transcytosis across the blood-brain barrier. More importantly, relying on abundant cytokine receptors on the NVs, siMETTL3-hNVs served as decoys to neutralize pro-inflammatory cytokines, synergizing with the intracellular silencing of METTL3 to drive microglial M2 repolarization. In female mouse models of acute neuroinflammation and radiation-induced brain injury, siMETTL3-hNVs treatment significantly reduced cytokine levels, attenuated hippocampal damage, and ameliorated cognitive deficits. This work overcomes critical delivery bottlenecks in m^6^A-based therapeutics and establishes a robust strategy for epigenetic reprogramming of neuroinflammation.

## Abstract (web summary)

Strategies to modulate neuroinflammation have broad therapeutic potential. Here, the authors develop siMETTL3-hNVs, nanovesicles carrying siRNA targeting METTL3, and show they can reduce neuroinflammation in vitro and in vivo.

## Introduction

Neuroinflammation is a central pathological process driving a broad range of neurological disorders, from acute conditions such as stroke, traumatic brain injury, multiple sclerosis, and radiation-induced brain injury (RIBI) to chronic neurodegenerative diseases, including Alzheimer’s and Parkinson’s disease^1^. Although the central nervous system (CNS) maintains active immune surveillance under homeostasis, disruption of this equilibrium is a key contributor to neuroinflammatory pathology^2^. Historically, conventional anti-inflammatory drugs, including non-steroidal anti-inflammatory drugs (NSAIDs) and glucocorticoids, have demonstrated limited efficacy against neuroinflammatory diseases and are often hampered by side effects that restrict their clinical utility^3^. To overcome these limitations, various biological agents and targeted therapies, for instance migration inhibitors and B-cell-depleting monoclonal antibodies, have been developed^4^. Many have been validated in preclinical and clinical studies, with some established as standard treatments for diseases like stroke and multiple sclerosis^5,6^. More recently, inhibitors targeting key inflammatory pathways, particularly Janus kinase inhibitors, have shown promise in preclinical models by suppressing aberrant signaling^7^. However, their clinical translation has been challenging owing to systemic adverse effects, such as increased susceptibility to infections and malignancies^8,9^. These limitations highlight the urgent need to further elucidate the regulatory networks underlying neuroinflammation, which may reveal safer and more durable therapeutic targets.

N6-methyladenosine (m^6^A), the most abundant internal mRNA modification in eukaryotes, is a pivotal post-transcriptional regulator of gene expression and plays a critical role in CNS homeostasis and disease^10^. This modification is dynamic and reversible, regulated by writer, eraser, and reader proteins, which enables precise control of RNA metabolism and the orchestration of complex biological processes such as neuroimmune responses^11–13^. Among these regulators, methyltransferase-like 3 (METTL3), which serves as the core catalytic subunit, is a key determinant of neuroinflammatory responses in diverse pathological contexts^14^. Mechanistic studies have established that dysregulated METTL3-mediated m^6^A modification contributes to glial cell activation, propagation of inflammatory signaling, and exacerbated pathology in acute and chronic neurological disorders^15–17^. Notably, suppressor of cytokine signaling 3 (SOCS3) is a key endogenous feedback inhibitor of inflammatory signaling that constrains Janus kinase 2/signal transducer and activator of transcription 3 (JAK2–STAT3) activation in myeloid cells, including microglia, thereby limiting neuroinflammatory amplification^18,19^. Emerging evidence further suggests that m^6^A-dependent regulation can modulate SOCS-family signaling^20^, and a recent study identified an alkB homolog 5, RNA demethylase (ALKBH5)-m^6^A-SOCS3 axis in microglia/macrophages that alleviates neuroinflammation after brain injury by stabilizing SOCS3 transcripts and suppressing JAK2–STAT3 signaling^21^. Together, these findings position SOCS3 as a key downstream effector linking epitranscriptomic regulation to neuroinflammatory control. Although targeting aberrant m^6^A signaling with small-molecule inhibitors or RNA interference has demonstrated therapeutic potential, clinical translation remains challenging due to issues including poor selectivity and potential systemic toxicity^22^. Thus, there is an urgent need to develop efficient delivery systems that improve both the safety and efficacy of m^6^A-targeted therapies.

Extracellular vesicles (EVs) are cell membrane-derived vesicles released by cells that function as key mediators of intercellular communication and biological process regulation^23,24^. Owing to their high biocompatibility and low immunogenicity, EVs have been engineered as next-generation drug delivery vehicles^25,26^. In particular, EVs represent attractive candidates due to their potential to orchestrate a beneficial shift in the immune milieu, such as by driving the transition of macrophages/microglia from an M1 to an M2 phenotype^27,28^. However, the limited production of secreted EVs significantly restricts their widespread application in immunotherapy and drug delivery^25^. To address this, cell-derived nanovesicles (NVs) have developed as an alternative to EVs by sonication or extrusion of cellular membranes^29^. These NVs inherit the lipid composition and surface proteins of their parent cells, preserving essential biological functions, such as immunomodulation^30^. For example, our previous work demonstrated that NVs can neutralize a broad spectrum of cytokines and mitigate pulmonary inflammation^31,32^. Furthermore, NVs are cell-cell communication media and thus can cross the blood-brain barrier (BBB), making them promising nanocarriers for treating neurological disorders^33^. To further enhance the therapeutic utility of NVs, particularly their drug-loading capacity, a compelling strategy is to fuse them with synthetic liposomes^34^. This hybrid approach synergizes the biological advantages of NVs with the high drug-loading capacity of liposomes, while compensating for the poor innate targeting capability of liposomes.

In this study, we developed a hybrid nanovesicle/liposome platform pre-loading small interfering RNA targeting METTL3 (siMETTL3-hNVs) to combine epitranscriptomic regulation with immunomodulation. By interfacing M2 microglial membranes with synthetic liposomes, this system coupled biomimetic delivery with gene silencing. Through C–C motif chemokine ligand 2 (CCL2)/C–C chemokine receptor type 2 (CCR2) chemotaxis and caveolae-mediated transcytosis across the BBB, siMETTL3-hNVs accumulated in inflamed brain regions. The therapeutic effects were associated with neutralization of the inflammatory milieu and restoration of SOCS3 stability through METTL3 knockdown. This process reduced JAK-STAT signaling and promoted M2 repolarization. In models of LPS-induced neuroinflammation and radiation injury, this strategy improved cognitive outcomes and showed a favorable safety profile, supporting a nanovesicle-based approach for CNS epitranscriptomic regulation.

## Results

### Preparation and characterization of siMETTL3-hNVs

**Fig. 1:**
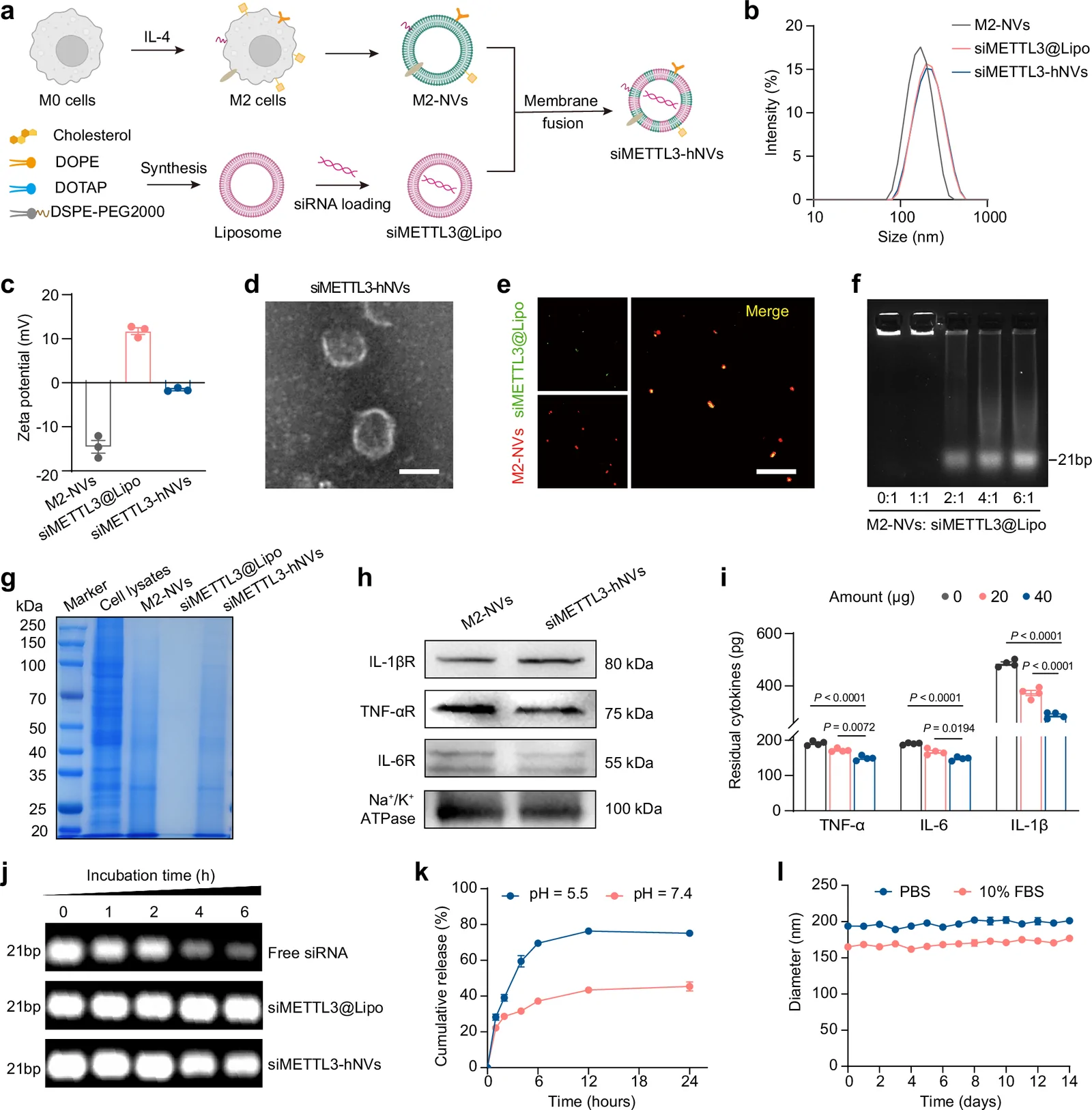
Preparation and characterization of siMETTL3-hNVs. **a** Schematic illustration of the preparation of siMETTL3-hNVs. Created in BioRender. Xu, L. (2026). https://BioRender.com/d164un1. **b**, **c** Hydrodynamic diameters (**b**) and zeta potentials (**c**) of M2-NVs, siMETTL3@Lipo and siMETTL3-hNVs, as determined by DLS (*n* = 3 independent experiments). **d** TEM image of siMETTL3-hNVs (*n* = 3 independent experiments). Scale bar, 100 nm. **e** Immunofluorescence images of siMETTL3-hNVs showing the colocalization of M2-NVs and siMETTL3@Lipo after fusion (*n* = 3 independent experiments). Scale bar, 10 μm. **f** Agarose gel electrophoresis analysis of siMETTL3-hNVs prepared with different mass ratios of M2-NVs to siMETTL3@Lipo (*n* = 3 independent experiments). **g** SDS-PAGE protein analysis of BV2 cell lysates, M2-NVs, siMETTL3@Lipo and siMETTL3-hNVs. **h** Western blot analysis of cytokine receptors, including interleukin-1β receptor (IL-1βR), interleukin-6 receptor (IL-6R) and TNF-αR, in M2-NVs and siMETTL3-hNVs. **i** Binding capacity of siMETTL3-hNVs toward TNF-α, IL-6 and IL-1β (*n* = 4 independent experiments; exact *P* values: TNF-α, 40 vs. 0 μg, *P* = 5.16E − 6; IL-6, 40 vs. 0 μg, *P* = 4.33E − 6; IL-1β, 40 vs. 0 μg, *P* = 3.57E − 21; 40 vs. 20 μg, *P* = 4.24E − 12). **j** Stability of siRNA in free form, siMETTL3@Lipo or siMETTL3-hNVs after incubation with fetal bovine serum for the indicated times, assessed by agarose gel electrophoresis (*n* = 3 independent experiments). **k** Cumulative release of siRNA from siMETTL3-hNVs in phosphate-buffered saline at pH 7.4 or 5.5 (*n* = 3 independent experiments). **l** Stability of siMETTL3-hNVs in phosphate-buffered saline or 10% fetal bovine serum over 14 days, determined by DLS (*n* = 3 independent experiments). For (**c**, **i**, **k**, **l**), data are presented as mean ± s.e.m., with statistical significance assessed using two-way ANOVA followed by Tukey’s multiple comparison test (**i**). Source data are provided as a Source Data file.

The preparation of siMETTL3-hNVs involved a three-step procedure: (1) obtaining M2-NVs; (2) preparing cationic liposomes encapsulating METTL3-siRNA (siMETTL3@Lipo); and (3) fabricating the final siMETTL3-hNVs by fusing M2-NVs with siMETTL3@Lipo (Fig. 1a). Initially, BV2 microglial cells were polarized to the M2 phenotype with interleukin-4 (IL-4), and then the polarization was confirmed by detecting CD206 expression via flow cytometry (Suppl. Fig. 1a). M2-NVs were generated from polarized cells by isolating plasma membranes and processing them into nanovesicles. Quantitative real-time PCR (qRT-PCR) analysis confirmed that M2-NVs exhibited significantly elevated expression levels of anti-inflammatory markers, including mannose receptor C-type 1 (*Mrc1*), arginase-1 (*Arg1*), and *Il4*, compared to M0-NVs (Suppl. Fig. 1b). Concurrently, cationic liposomes were prepared *via* thin-film hydration and then complexed with METTL3-siRNA to obtain siMETTL3@Lipo. After that, siMETTL3-hNVs were subsequently fabricated by fusing M2-NVs with siMETTL3@Lipo through a process of mixing, sonication, and extrusion. Thus, siMETTL3-hNVs were successfully prepared.

Transmission electron microscopy (TEM) and dynamic light scattering (DLS) analysis showed that all three nanoparticle types (M2-NVs, siMETTL3@Lipo, and siMETTL3-hNVs) exhibited spherical morphologies, with average diameters of 150.9 nm, 198.8 nm, and 190 nm, respectively (Fig. 1b–d; Suppl. Fig. 1c). To verify the membrane fusion, single NVs were pre-labeled with distinct fluorescent dyes, and confocal imaging of siMETTL3-hNVs revealed significant overlap of fluorescent signals (Fig. 1e), confirming successful membrane fusion. Furthermore, Förster resonance energy transfer (FRET) assays were conducted to monitor fusion. Upon mixing siMETTL3@Lipo with M2-NVs at mass ratios of 1:1 and 1:2, a characteristic decrease in donor emission and a concurrent increase in acceptor emission were observed, yielding FRET efficiencies of 35.2% and 41.1%, respectively (Suppl. Fig. 1d). Based on these results, a mass ratio of 1:1 (siMETTL3@Lipo to M2-NVs) was selected as optimal for the preparation of siMETTL3-hNVs (Fig. 1f; Suppl. Fig. 1e). Western blot analysis further confirmed that siMETTL3-hNVs retained the characteristic protein profile of M2-NVs, including the cytokine receptors IL-6R, IL-1βR, and tumor necrosis factor alpha receptor (TNF-αR) (Fig. 1g, h). Functionally, by leveraging these inherited receptors, siMETTL3-hNVs displayed a dose-dependent cytokine-scavenging capacity toward IL-6, IL-1β, and TNF-α (Fig. 1i; Suppl. Fig. 1f–h). Notably, 40 μg of siMETTL3-hNVs removed 42.01 ± 6.42 pg of TNF-α, 42.62 ± 5.16 pg of IL-6, and 198.47 ± 11.63 pg of IL-1β, further confirming their substantial cytokine-binding capability. Together, these data confirmed the fabrication of siMETTL3-hNVs as a hybrid nanoplatform with immunomodulatory properties.

The protective capacity of siMETTL3-hNVs for encapsulated siRNA was confirmed by a serum stability assay, and a distinct electrophoretic band remaining after digestion in the siMETTL3@Lipo and siMETTL3-hNVs groups indicated enhanced siRNA stability (Fig. 1j). Furthermore, the in vitro release profile of siMETTL3-hNVs was pH-responsive, with approximately 69.53% ± 2.79% of the siMETTL3 payload released within 6 hours at pH 5.5 (Fig. 1k). This accelerated release under acidic conditions can be attributed to the incorporation of 1,2-dioleoyl-sn-glycero-3-phosphoethanolamine (DOPE), which promotes endosomal membrane destabilization and facilitates cytosolic delivery of the cargo^34^. Additionally, siMETTL3-hNVs maintained structural stability for at least 14 days in buffer solutions (Fig. 1l) and demonstrated limited cytotoxicity and hemolytic activity in vitro (Suppl. Fig. 1i, j). These stability and biocompatibility profiles supported subsequent in vitro and in vivo applications.

### siMETTL3-hNVs exhibit enhanced inflamed-brain delivery and preferential uptake by microglia

**Fig. 2:**
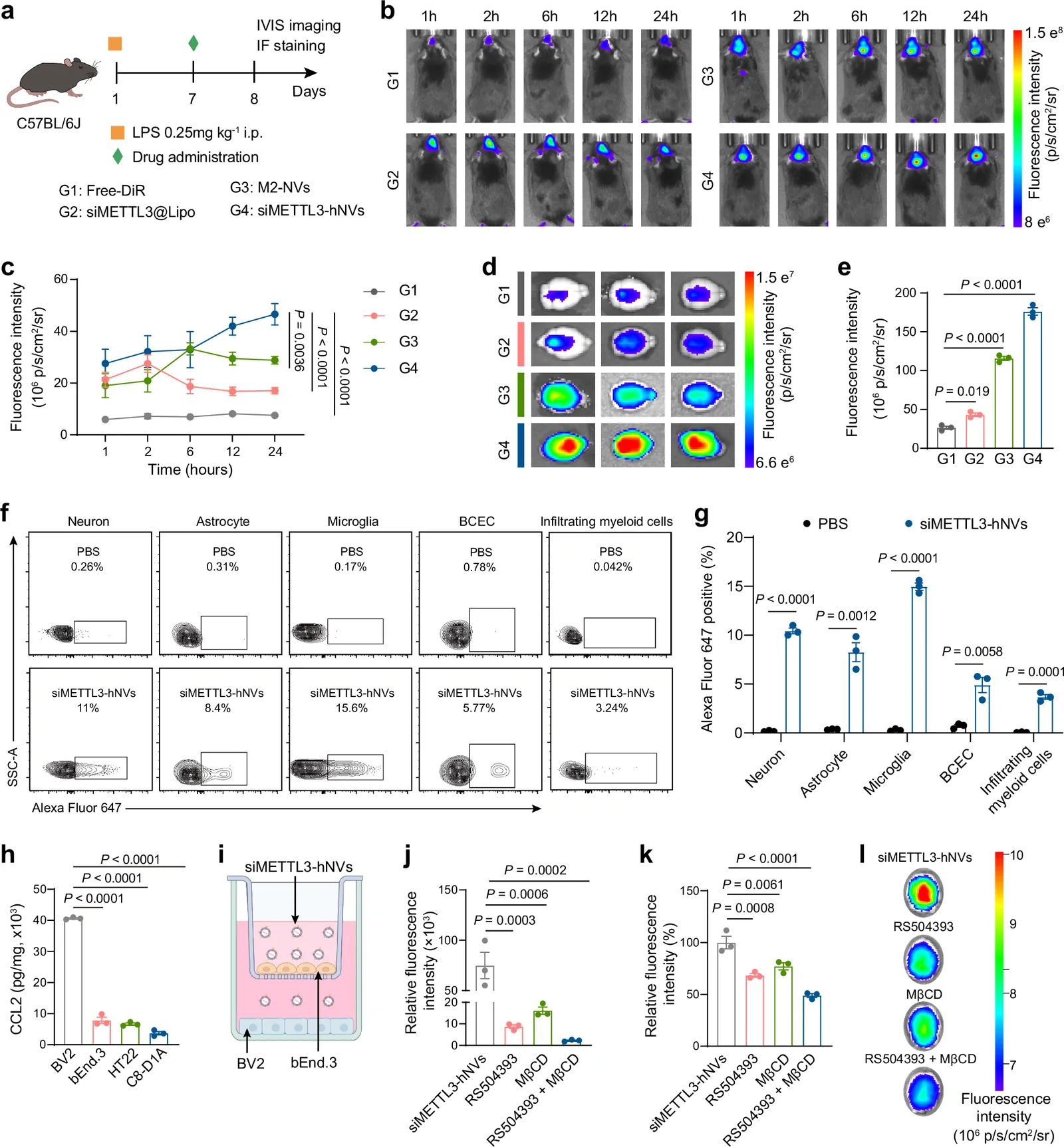
Brain delivery, cellular uptake, and biodistribution of siMETTL3-hNVs. **a** Schematic illustrating intravenous administration and in vivo imaging in the LPS-induced neuroinflammatory mouse model. **b**, **c** In vivo fluorescence images (**b**) and quantification of brain fluorescence intensity (**c**) at different time points after injection of Free-DiR, DiR-labelled siMETTL3@Lipo, M2-NVs or siMETTL3-hNVs (*n* = 3 biologically independent mice; exact *P* values: Free-DiR vs. siMETTL3-hNVs *P* = 3.48E − 9; siMETTL3@Lipo vs. siMETTL3-hNVs *P* = 1.89E − 6). **d, e** Ex vivo fluorescence images (**d**) and quantification (**e**) of isolated brains collected 24 h post-injection (*n* = 3 biologically independent brain samples; exact *P* values: Free-DiR vs. M2-NVs *P* = 1.66E − 7; Free-DiR vs. siMETTL3-hNVs *P* = 2.79E − 9). **f**, **g** Flow cytometry analysis (**f**) and quantification (**g**) of Alexa Fluor 647-positive cells among brain cell populations after intravenous administration of siMETTL3-hNVs (*n* = 3 biologically independent brain samples; exact *P* values: PBS vs. siMETTL3-hNVs in neurons *P* = 4.32E − 6; microglia *P* = 2.76E − 6). **h**, ELISA analysis of CCL2 levels in culture supernatants from BV2, bEnd.3, HT22 and C8-D1A cells after LPS stimulation (*n* = 3 independent experiments; exact *P* values: BV2 vs. bEnd.3 *P* = 6.44E − 10; BV2 vs. HT22 *P* = 4.81E − 10; BV2 vs. C8-D1A *P* = 2.48E − 10). **i**, Schematic of the bEnd.3–BV2 Transwell assay for BBB penetration assessment. **j**, Fluorescence intensity of lower-chamber BV2 cells after treatment with siMETTL3-hNVs, RS504393-pretreated siMETTL3-hNVs or MβCD-pretreated bEnd.3 cells (*n* = 3 independent experiments). **k**, **l** Quantification of brain fluorescence intensity (**k**) and representative IVIS images (**l**) after intravenous injection of siMETTL3-hNVs, including mice pretreated with MβCD or receiving RS504393-pretreated siMETTL3-hNVs (*n* = 3 biologically independent mice; exact *P* value: siMETTL3-hNVs vs. RS504393 + MβCD *P* = 2.71E − 5). All animal experiments in this figure were performed using six-week-old female C57BL/6J mice. For (**c**, **e**, **g**, **h**, **j**, **k**), data are presented as mean ± s.e.m., with statistical significance assessed using one-way ANOVA followed by Tukey’s multiple comparison test (**e**, **h**, **j**, **k**) or two-way ANOVA (**c**) and a two-tailed Student’s t-test (**g**). Source data are provided as a Source Data file. Panel (**a**, **i**) created in BioRender. Xu, L. (2026) https://BioRender.com/of7bkyt.

To evaluate the in vivo distribution of siMETTL3-hNVs, we employed an LPS-induced neuroinflammation model in six-week-old female C57BL/6J mice (Fig. 2a; Suppl. Fig. 2a–f). 1,1’-dioctadecyl-3,3,3’,3’-tetramethylindotricarbocyanine iodide (DiR)-labeled nanoparticles were administered via tail vein injection, and the brain-targeting capability of siMETTL3-hNVs was assessed using an IVIS imaging system. The accumulation of both M2-NVs and siMETTL3-hNVs in the brain was significantly higher than that of Free-DiR and siMETTL3@Lipo (Fig. 2b). This enhanced accumulation is likely mediated by the adhesive properties of surface protein molecules on the NVs^35^. Quantitative fluorescence analysis further demonstrated that siMETTL3-hNVs yielded the highest cerebral fluorescence intensity among all groups at 12 hours post-injection (Fig. 2c). Ex vivo imaging of harvested brains confirmed higher brain accumulation of siMETTL3-hNVs, showing fluorescence intensities 4.1-fold and 1.5-fold higher than those of siMETTL3@Lipo and M2‑NVs, respectively (Fig. 2d, e; Suppl. Fig. 2g, h). These results indicate that incorporation of the M2-NV membrane improves inflamed-brain delivery, whereas the higher performance of siMETTL3-hNVs over native M2-NVs likely reflects the hybrid formulation, which preserves membrane-associated recognition while improving siRNA loading and physicochemical robustness^36^.

To initially explore whether siMETTL3-hNVs exhibit cell-type-dependent uptake, we first compared their internalization across several brain-relevant cell types in vitro. siMETTL3-hNVs showed higher uptake in BV2 microglia than in C8-D1A astrocytes, HT22 neurons, and bEnd.3 endothelial cells (Suppl. Fig. 3a–e), and BV2 uptake increased over time (Suppl. Fig. 3f). In vivo flow cytometric analysis at 12 h after a single intravenous injection of Alexa Fluor 647-labeled siMETTL3-hNVs showed that microglia exhibited the highest uptake among the brain cell populations examined (14.97 ± 0.65%), followed by neurons (10.43 ± 0.51%), astrocytes (8.25 ± 1.67%), brain capillary endothelial cells (BCECs, 4.90 ± 1.33%) and infiltrating myeloid cells (3.67 ± 0.44%) (Fig. 2f, g; Suppl. Fig. 3g). Notably, although siMETTL3-hNVs were distributed to multiple brain-resident cell populations, uptake was consistently highest in microglia, in agreement with the in vitro results. This preferential microglial distribution was further supported by tissue-level co-localization of siMETTL3-hNVs with IBA1^+^ microglia in the inflamed brain (Suppl. Fig. 3h, i).

### Mechanistic investigation of inflamed-brain delivery by siMETTL3-hNVs

LPS-induced neuroinflammation is associated with partial BBB disruption^37^. Consistent with this, Evans blue extravasation confirmed increased BBB permeability in our model (Suppl. Fig. 4a, b). Based on this result, we were interested in answering a critical question as to how siMETTL3-hNVs crossed the BBB, namely whether their brain accumulation arose solely from passive leakage or also involved active transport processes. In this regard, the marked differences in brain accumulation among nanoparticle formulations under the same inflammatory conditions (Fig. 2b–e) suggested that passive leakage alone could not fully account for the superior brain delivery of siMETTL3-hNVs.

Chemokine-guided enrichment may contribute to inflamed-brain delivery^38^. CCL2 is known to be upregulated during neuroinflammation^39^. Consistent with this, ELISA analysis showed that, among BV2 microglia, bEnd.3 endothelial cells, HT22 neurons and C8-D1A astrocytes, BV2 cells secreted the highest level of CCL2 after LPS stimulation (Fig. 2h), supporting microglia as a major contributor to CCL2 production under our experimental conditions. In parallel, Western blot analysis confirmed increased CCR2 expression in IL-4-polarized M2-BV2 cells, indicating that this receptor could be inherited by siMETTL3-hNVs from the donor membrane (Supplementary Fig. 4c). To assess the contribution of the CCR2–CCL2 axis, a bEnd.3-BV2 Transwell model under inflammatory conditions was established as described previously (Supplementary Fig. 4d, e)^40^, and we found that pretreatment of siMETTL3-hNVs with RS504393 (a selective CCR2 antagonist) significantly reduced the fluorescence signal in the lower chamber (Fig. 2i, j, Supplementary Fig. 4f-j). Consistently, in vivo imaging showed that mice receiving RS504393-pretreated siMETTL3-hNVs exhibited markedly lower brain fluorescence intensity after intravenous injection (Fig. 2k, l). These results support the interpretation that the CCR2-CCL2 axis acts as a chemotactic cue that promotes inflammatory-site homing and brain enrichment of siMETTL3-hNVs.

BBB translocation depends on the interaction between endothelial cells and nanocarriers and may involve multiple uptake pathways determined by nanoparticle size, shape, surface chemistry, charge, and morphology^41,42^. In the bEnd.3-BV2 Transwell model, methyl-β-cyclodextrin (MβCD, a caveolae-mediated endocytic inhibitor) reduced the transcytosis efficiency of siMETTL3-hNVs by 72.92 ± 0.19%, whereas chlorpromazine (CPZ, a clathrin-mediated endocytic inhibitor) and 5-(N-ethyl-N-isopropyl)-amiloride (EIPA, a macropinocytosis-mediated endocytic inhibitor) showed no significant effect (Suppl. Fig. 4k, l). This result suggests that endothelial transport of siMETTL3-hNVs may preferentially rely on a caveolae-associated pathway. MβCD also significantly decreased fluorescence signals in both upper-chamber bEnd.3 cells and lower-chamber BV2 cells (Fig. 2j), and pretreatment of mice with MβCD similarly reduced brain fluorescence intensity after injection of DiR-labeled siMETTL3-hNVs in vivo, with a further reduction observed when combined with RS504393-pretreated siMETTL3-hNVs (Fig. 2k, l). Together, these findings support that caveolae might be critical mediators in facilitating the BBB crossing of siMETTL3-hNVs, in line with previous studies^43,44^.

To further evaluate the inflammation dependence of this process, we compared brain accumulation of siMETTL3-hNVs in healthy mice and LPS-induced neuroinflammatory mice with different degrees of inflammation. Brain accumulation was markedly lower in healthy mice and increased with inflammatory severity, in parallel with elevated CCL2 levels in brain tissue (Suppl. Fig. 4m–o). These results are consistent with a CCL2-related chemokine-guided enrichment process. Collectively, these data support a model in which siMETTL3-hNVs achieve enhanced inflamed-brain delivery through the combined effects of CCR2-CCL2 chemotaxis, caveolae-mediated transcytosis, and preferential uptake by microglia.

### Epigenetic regulation of microglia promotes M2 polarization and rescues neuronal viability

Efficient cytosolic delivery of siRNA is a prerequisite for effective gene silencing^45^. To evaluate the delivery efficiency of our nanomodulator, we first assessed the cellular trafficking of siMETTL3-hNVs in BV2 microglial cells. Confocal microscopy tracking of DiD-labeled siMETTL3-hNVs revealed a time-dependent localization pattern. At 6 h post-treatment, the DiD signal predominantly colocalized with lysosomes, indicating active endocytic uptake. Notably, by 8 hours, the fluorescence had redistributed into a diffuse cytoplasmic pattern (Suppl. Fig. 5a), demonstrating successful lysosomal escape of the siRNA cargo, a step for functional cytosolic delivery. Consequently, treatment with siMETTL3-hNVs led to a significant suppression of both *Mettl3* mRNA and METTL3 protein expression in BV2 cells (Suppl. Fig. 5b, c), confirming effective target gene knockdown. In line with the pivotal role of METTL3 as the core methyltransferase, this knockdown resulted in a global reduction of m^6^A modification levels in these cells (Suppl. Fig. 5d, e). Collectively, these results verify that siMETTL3-hNVs deliver functional siRNA into the microglial cytosol, effectively depleting METTL3 and its associated m^6^A methylation activity.

**Fig. 3:**
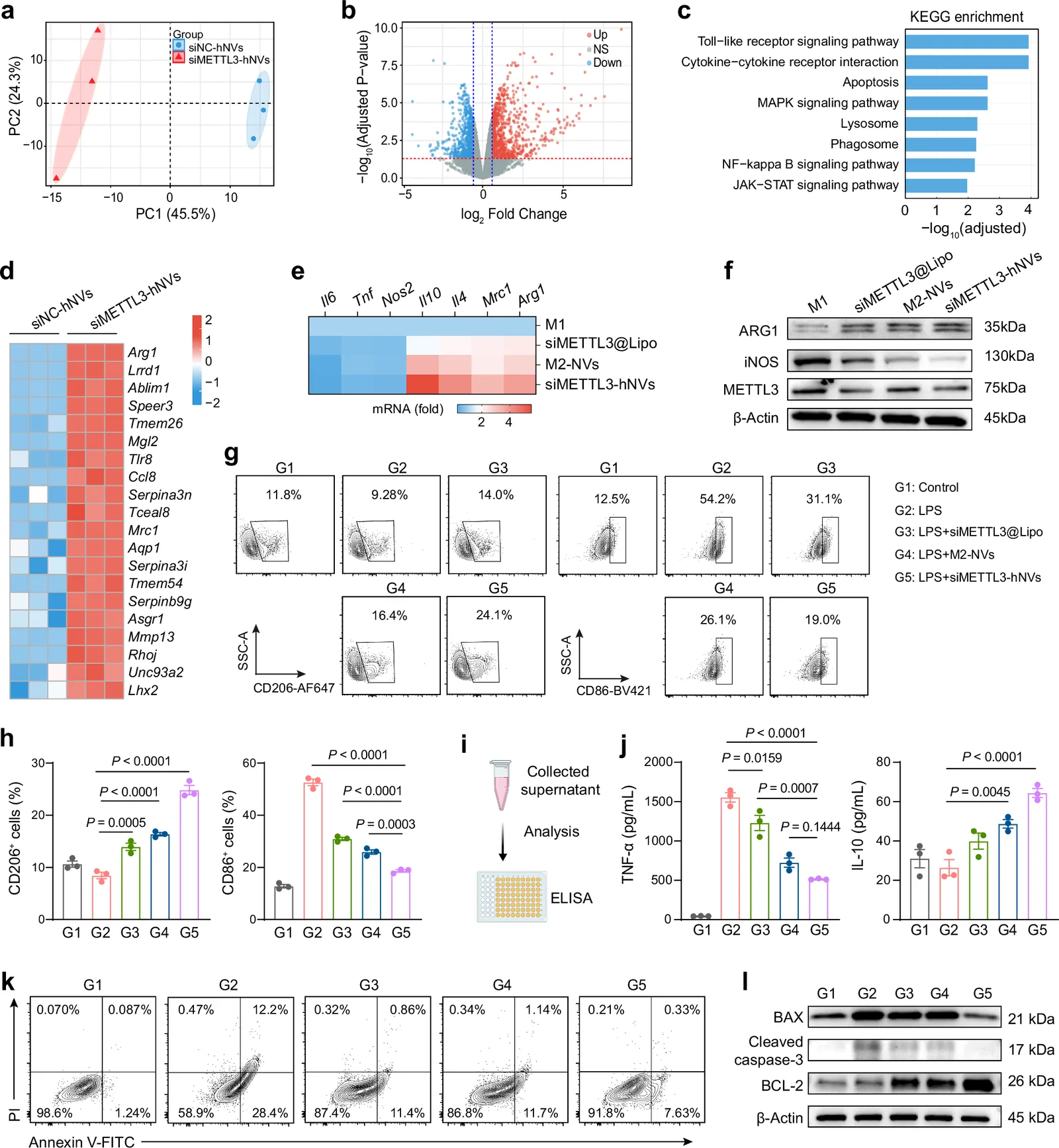
Epigenetic reprogramming of microglia by siMETTL3-hNVs confers anti-inflammatory and neuroprotective effects. **a** Principal component analysis of RNA-seq data from BV2 microglial cells treated with siNC-hNVs or siMETTL3-hNVs (*n* = 3 independent experiments). **b** Volcano plot showing differentially expressed genes between siNC-hNVs and siMETTL3-hNVs. **c** KEGG pathway enrichment analysis of the differentially expressed genes identified in (**b**). **d** Heatmap of selected differentially expressed genes in BV2 cells treated with siMETTL3-hNVs or siNC-hNVs (*n* = 3 independent experiments). **e** qRT-PCR analysis of M1 marker genes (*Il6*, *Tnf* and *Nos2*) and M2 marker genes (*Il10*, *Il4*, *Mrc1* and *Arg1*) in LPS-stimulated BV2 cells after the indicated treatments (*n* = 3 independent experiments). **f** Western blot analysis of ARG1, iNOS and METTL3 expression in LPS-stimulated BV2 cells after various treatments. **g**, **h** Flow cytometry analysis (**g**) and quantification (**h**) of CD206^+^ M2-like and CD86^+^ M1-like BV2 cells after treatment (*n* = 3 independent experiments; CD206 exact *P* values: G2 vs. G4 *P* = 2.05E − 5; G2 vs. G5 *P* = 8.21E − 9; CD86 exact *P*-values: G5 vs. G2 *P* = 3.14E − 13; G5 vs. G3 *P* = 3.08E − 6). **i** Schematic of conditioned-medium collection from BV2 cells after different treatments for ELISA analysis. Created in BioRender. Xu, L. (2026) https://BioRender.com/waa4xm2. **j** ELISA analysis of TNF-α and IL-10 levels in conditioned medium collected from BV2 cells after the indicated treatments (*n* = 3 independent experiments; exact *P* values: TNF-α, G2 vs. G5 *P* = 1.17E − 6; IL-10, G2 vs. G5 *P* = 7.34E − 5). **k**, **l** Flow cytometry analysis of HT22 cell apoptosis (**k**) and immunoblot analysis of apoptosis-related proteins (**l**) after treatment with different preparations. G1, Control; G2, LPS; G3, LPS + siMETTL3@Lipo; G4, LPS + M2-NVs; G5, LPS + siMETTL3-hNVs. For (**h**, **j**), data are presented as mean ± s.e.m., with statistical significance assessed using one-way ANOVA followed by Tukey’s multiple comparison test. Source data are provided as a Source Data file.

Aberrant epigenetic modifications and inflammatory microenvironment remodeling jointly drive the epigenetic reprogramming of microglia, polarizing them toward a pro‑inflammatory (M1) state^16^. This process exacerbates deleterious neuroimmune responses and contributes to disease progression^17,46,47^. To delineate how METTL3 silencing modulates microglial phenotype, we performed transcriptomic profiling of BV2 cells treated with siMETTL3-hNVs, with cells receiving siNC-hNVs serving as the control (Fig. 3a). This treatment induced substantial transcriptional changes, yielding 1,592 differentially expressed genes (DEGs) (Fig. 3b). Kyoto Encyclopedia of Genes and Genomes (KEGG) pathway analysis of these DEGs revealed their significant enrichment in the JAK-STAT and NF-κB signaling pathways (Fig. 3c), two key cascades governing inflammatory responses. Consistent with an anti-inflammatory shift, classical markers of M2 activation, such as *Arg1*, *Mgl2*, and *Mrc1*, were upregulated upon METTL3 depletion (Fig. 3d). Furthermore, Gene Set Enrichment Analysis (GSEA) corroborated this phenotype switch by showing that METTL3 knockdown was significantly associated with the negative regulation of key M1-related cytokines, including IL-12 and tumor necrosis factor (Suppl. Fig. 5f). Taken together, these transcriptomic data demonstrate that silencing METTL3 reprograms microglia by simultaneously suppressing pivotal pro-inflammatory pathways and activating a repertoire of genes characteristic of the restorative M2 phenotype.

To validate these transcriptomic findings under inflammatory conditions, BV2 cells were stimulated with LPS to induce classical M1 polarization. qRT‑PCR analysis confirmed that siMETTL3‑hNVs treatment significantly downregulated the expression of key M1 markers (*Tnf*, *Il6*, *Il1b* and *Nos2*) and concurrently upregulated characteristic M2 markers (*Arg1*, *Mrc1* and *Il4*) (Fig. 3e; Suppl. Fig. 5g). Western blot analysis further showed the stronger effect of siMETTL3‑hNVs: compared to untreated cells, siMETTL3@Lipo, and M2‑NVs controls, it increased the protein level of the M2 marker ARG1 and decreased that of the M1 marker iNOS (Fig. 3f). Moreover, siMETTL3‑hNVs were also capable of directly repolarizing resting (M0) BV2 cells toward an M2‑like phenotype (Suppl. Fig. 5h, i), indicating that its effect is not limited to inflammatory contexts. Flow cytometric analysis of surface markers quantitatively confirmed this phenotypic shift, revealing a significant increase in the proportion of CD206⁺ M2‑like cells and a concomitant decrease in CD86⁺ M1‑like cells following siMETTL3‑hNVs treatment (Fig. 3g, h). Consistent with this functional repolarization, ELISA showed that siMETTL3-hNVs reduced pro-inflammatory cytokines (TNF-α, IL-6, and IL-1β) while increasing anti-inflammatory cytokines (IL-4 and IL-10) (Fig. 3i, j; Suppl. Fig. 5j–l). Moreover, M2-NVs reduced TNF-α, IL-6, and IL-1β to a greater extent than siMETTL3@Lipo, by 2.8 ± 0.36-fold, 1.87 ± 0.28-fold, and 1.5 ± 0.19-fold, respectively, supporting a substantial contribution of membrane-mediated cytokine scavenging to the overall anti-inflammatory effect. Collectively, these results indicate that siMETTL3-hNVs can reprogram microglial polarization from a pro-inflammatory to an anti-inflammatory state.

**Fig. 4:**
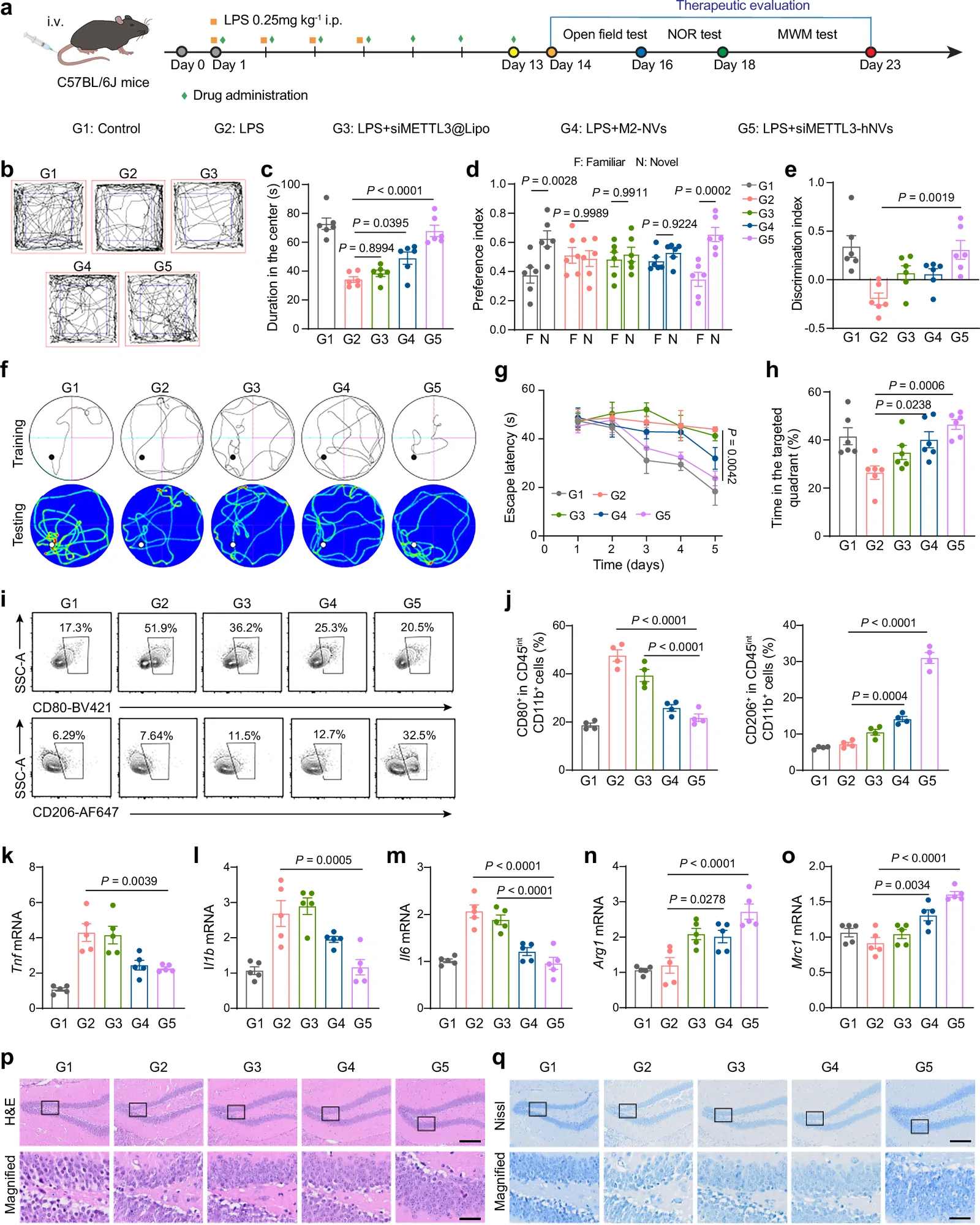
Cognition and memory deficit rescue of siMETTL3-hNVs in a LPS-induced mouse model. **a** Schematic of the treatment regimen in the LPS-induced neuroinflammation mouse model. Created in BioRender. Xu, L. (2026) https://BioRender.com/p3dgl4h. **b**, **c** Movement tracings in the open-field test (**b**) and quantification of time spent in the centre zone (**c**) (*n* = 6 biologically independent mice; exact *P*-value: G2 vs. G5 *P* = 2.54E − 6). **d**, **e** Quantification of the preference index (**d**) and discrimination index (**e**) in the novel object recognition test (*n* = 6 biologically independent mice). **f**–**h** Swim paths and heatmaps in the Morris water maze test (**f**), escape latency during training (**g**) and time spent in the target quadrant during the probe trial (**h**) (*n* = 6 biologically independent mice). **i,j**, Flow cytometry analysis (**i**) and quantification (**j**) of CD80^+^ M1-like and CD206^+^ M2-like microglia (*n* = 4 biologically independent brain samples; CD80 exact *P* values: G2 vs. G5 *P* = 5.37E − 7; G3 vs. G5 *P* = 6.07E − 5; CD206 exact *P* value: G2 vs. G5 *P* = 4.18E − 11). **k**–**o** qRT-PCR analysis of M1 marker genes (*Tnf*, *Il1b* and *Il6*) and M2 marker genes (*Arg1* and *Mrc1*) in brain tissues (*n* = 5 biologically independent brain samples; *Il6* exact *P*-values: G2 vs. G5 *P* = 2.64E − 6; G3 vs. G5 *P* = 3.33E − 5; *Arg1* exact *P* value: G2 vs. G5 *P* = 5.27E − 5; *Mrc1* exact *P*-value: G2 vs. G5 *P* = 3.27E − 6). **p**, **q** Representative haematoxylin and eosin (H&E) staining (**p**) and Nissl staining (**q**) of hippocampal sections after different treatments (*n* = 3 biologically independent brain samples). Scale bars, 100 μm for the upper panels and 20 μm for the magnified lower panels. G1, Control; G2, LPS; G3, LPS + siMETTL3@Lipo; G4, LPS + M2-NVs; G5, LPS + siMETTL3-hNVs. All behavioral and tissue analyses in this figure were performed using six-week-old female C57BL/6J mice. For (**c**–**e**, **g**, **h**, **j**–**o**), data are presented as mean ± s.e.m., with statistical significance assessed using one-way ANOVA followed by Tukey’s multiple comparison test (**c**, **e**, **h**, **j**–**o**) or two-way ANOVA (**d**, **g**). Source data are provided as a Source Data file.

Microglial behavior critically regulates neuronal activity, with microglia-neuron interactions being pivotal for maintaining neuroimmune homeostasis^48^. Accordingly, preserving neuronal survival and synaptic circuit integrity is essential for halting neurodegenerative disease progression^49^. To evaluate the neuroprotective efficacy of siMETTL3-hNVs, HT22 hippocampal neurons were cultured in conditioned medium collected from BV2 microglia subjected to different treatments (Suppl. Fig. 6a). Neuronal survival was significantly higher in the siMETTL3-hNVs group (83.6%) than in the PBS control group (61.0%) (Suppl. Fig. 6b). In a direct microglia-neuron co-culture system, MAP2 immunofluorescence staining revealed that siMETTL3-hNVs treatment markedly attenuated LPS-induced dendritic damage (Suppl. Fig. 6c, d), indicating its potential to support neural circuit integrity. Flow cytometric analysis further confirmed that siMETTL3-hNVs significantly reduced the apoptosis rate of HT22 cells from 40.6% to 7.96% (Fig. 3k). At the molecular level, siMETTL3-hNVs treatment upregulated the anti-apoptotic protein BCL-2, downregulated the pro-apoptotic protein BAX, and suppressed the activation of the key apoptotic protease caspase-3, as evidenced by reduced levels of cleaved caspase-3 (Fig. 3l). Collectively, these findings demonstrated that the neuroprotective effects of siMETTL3-hNVs were mediated by modulating the mitochondrial BCL-2/BAX pathway^50^, thereby inhibiting downstream caspase-3 activation.

### siMETTL3-hNVs ameliorate neuroinflammation and rescue cognitive function via microglial reprogramming

The therapeutic efficacy of siMETTL3-hNVs against neuroinflammation was evaluated in a mouse model, with post-treatment cognitive function assessed through a series of behavioral tests (Fig. 4a). Treatment with siMETTL3-hNVs attenuated weight loss and accelerated recovery compared to the PBS group (Suppl. Fig. 7a). Motor function was first assessed using the open field test. Compared to the model group, mice treated with siMETTL3-hNVs showed significant increases in total distance moved, average speed, time spent in the center, and number of entries into the center zone (Fig. 4b, c; Suppl. Fig. 7b–d). Cognitive and memory functions were then evaluated using the novel object recognition test. The siMETTL3-hNVs-treated group displayed a strong preference for the novel object (Fig. 4d, e), with a discrimination index comparable to that of healthy controls, indicating restored cognitive function.

In the Morris water maze test, siMETTL3-hNVs-treated mice demonstrated significantly enhanced spatial learning, as indicated by shorter escape latencies across training days and a greater preference for the target quadrant in the probe trial (Fig. 4f). During the probe trial on day 6, the siMETTL3-hNVs group spent significantly more time in the target quadrant and crossed the original platform location more frequently than the control groups (Fig. 4g, h; Suppl. Fig. 7e, f), indicating improved spatial memory. In contrast, the behavioral performance of the siMETTL3@Lipo group remained comparable to that of the disease model controls, suggesting that the hybrid nanovesicle design contributed to the observed therapeutic effect.

The persistent pro-inflammatory polarization of microglia constitutes a major barrier to resolving neuroinflammation, as these cells sustain a cytotoxic milieu that accelerates neuronal degeneration^51,52^. Consequently, therapeutically targeting epigenetic regulators to reprogram microglial phenotypes may help interrupt this pathological cycle. Flow cytometric analysis confirmed that siMETTL3-hNVs effectively reversed microglial polarization, decreasing the CD80⁺ pro-inflammatory population to 20.5% and increasing the CD206⁺ anti-inflammatory population to 32.5% (Fig. 4i, j; Supplementary Fig. 7g). The phenotypic shift was further corroborated by the transcriptional downregulation of pro-inflammatory genes (*Il1b*, *Il6* and *Tnf*) and upregulation of anti-inflammatory genes (*Arg1 *and *Mrc1*) (Fig. 4k–o). Furthermore, ELISA showed that siMETTL3-hNVs administration significantly reduced levels of pro-inflammatory cytokines (TNF-α, IL-6) in hippocampal and cortical tissues (Suppl. Fig. 7h, i). This reduction in inflammatory mediators contributed to a broader shift in the glial phenotypic landscape (Suppl. Fig. 7j, k). Consequently, the ameliorated inflammatory milieu enhanced neurotrophic support, as reflected by the restoration of Nissl body density in hippocampal neurons to near-normal levels (Fig. 4p, q; Suppl. Fig. 7l). Collectively, these findings indicate that siMETTL3-hNVs ameliorate neuroinflammation by modulating microglial phenotypes and reducing the cytotoxic inflammatory milieu, thereby supporting neuronal protection in this model.

### METTL3 knockdown inhibits the JAK2/STAT3 pathway through the m^6^A-SOCS3 axis in neuroinflammation

**Fig. 5:**
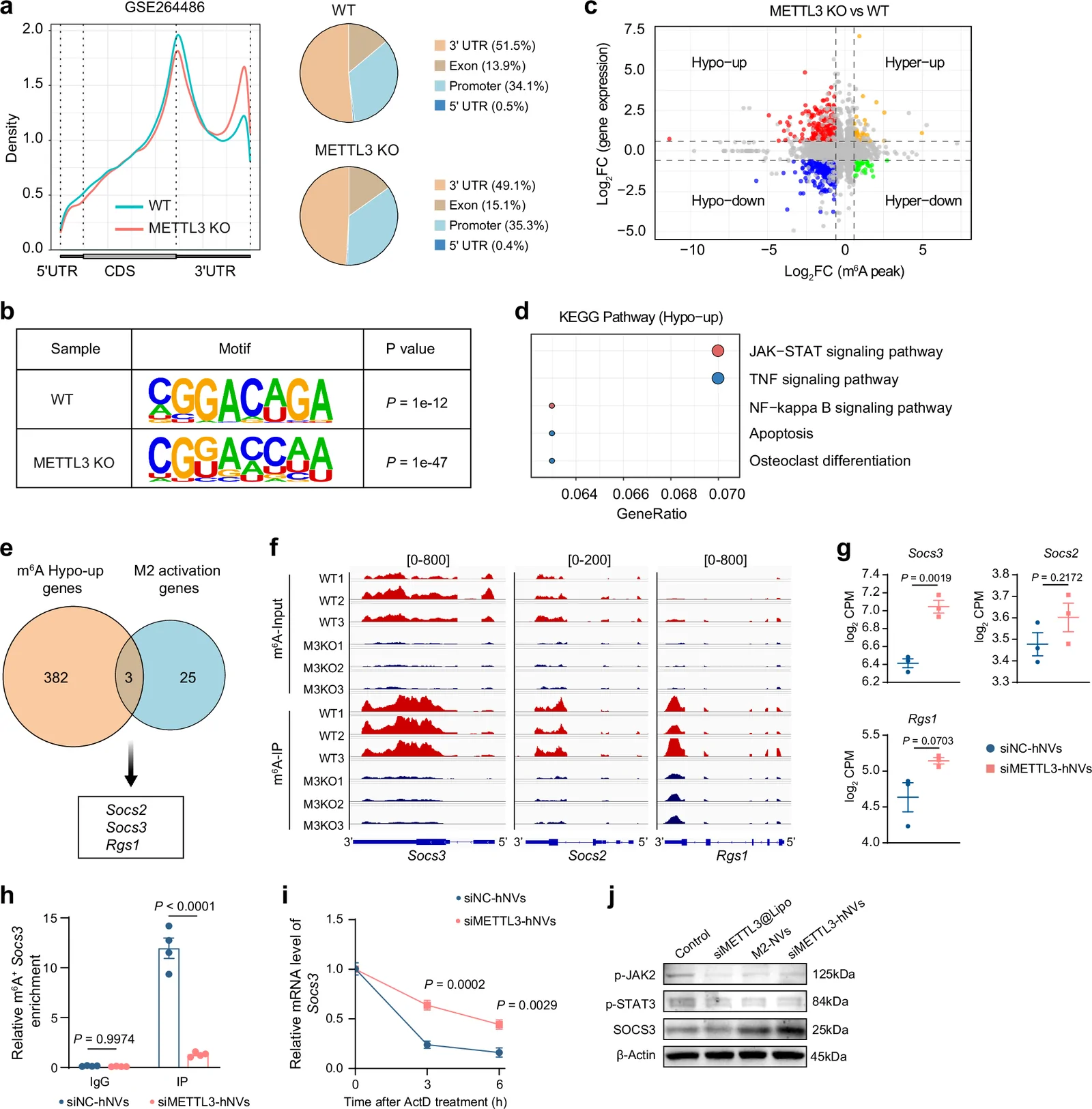
METTL3 knockdown drives microglial M2 polarization via the m^6^A-SOCS3-JAK2-STAT3 axis. **a** Genome-wide distribution of m^6^A peaks across methylated transcripts in LPS-stimulated BV2 cells from wild-type (WT) and METTL3-knockout (KO) groups. Data were derived from the public dataset GSE264486. **b** Motif enrichment analysis of m^6^A peaks from the same public dataset. **c** Correlation between gene expression changes from RNA-seq and m^6^A methylation changes from m^6^A-seq upon METTL3 knockout. **d** KEGG pathway enrichment analysis of genes that were both hypomethylated and upregulated in METTL3-KO cells. **e** Venn diagram showing the overlap between m^6^A-hypomethylated/upregulated genes in METTL3-deficient BV2 cells and curated M2-activation-associated genes. **f** m^6^A-seq read profiles across the genomic loci of *Socs3*, *Socs2* and *Rgs1* from the public dataset. **g** Normalized RNA-seq expression levels of *Socs3*, *Socs2* and *Rgs1* in BV2 cells treated with siNC-hNVs or siMETTL3-hNVs, shown as log_2_CPM values (*n* = 3 independent experiments). **h** MeRIP-qPCR validation of m^6^A enrichment on *Socs3* transcripts in BV2 cells treated with siNC-hNVs or siMETTL3-hNVs (*n* = 3 independent experiments; exact *P*-value: siNC-hNVs vs. siMETTL3-hNVs in IP *P* = 1.04E − 8). **i** RNA stability assay measuring *Socs3* mRNA decay after actinomycin D treatment in BV2 cells treated with siNC-hNVs or siMETTL3-hNVs (*n* = 3 independent experiments). **j** Western blot analysis of phosphorylated JAK2, phosphorylated STAT3 and total SOCS3 protein levels in BV2 cells after the indicated treatments. For **g**–**i** data are presented as mean ± s.e.m., with statistical significance assessed using two-tailed Student’s t-test (**g**) and two-way ANOVA followed by Tukey’s multiple comparison test (**h**, **i**). Source data are provided as a Source Data file.

METTL3 is a major methyltransferase responsible for m^6^A deposition^53^. To elucidate how METTL3-dependent m^6^A modification regulates microglial differentiation, we analyzed a public m^6^A‑sequencing (m^6^A‑seq) dataset (GSE264486). We first characterized the transcriptome-wide distribution of m^6^A. Peaks were predominantly enriched in the 3’ untranslated regions (3’ UTRs) of mRNAs (Fig. 5a). Motif analysis identified a strong enrichment for the canonical RRACH sequence (Fig. 5b), consistent with known m^6^A consensus^54^. Integrated analysis of m^6^A‑seq and RNA‑seq data identified transcripts that were both hypomethylated and upregulated upon METTL3 knockdown as direct potential targets (Fig. 5c). Pathway enrichment analysis revealed that these targets were significantly associated with several key signaling pathways, including the JAK‑STAT pathway (Fig. 5d). The JAK‑STAT pathway is a central regulator of immune cell polarization, whose hyperactivation typically drives pro‑inflammatory responses^55^. We then intersected these m^6^A‑hypomethylated potential targets with a curated set of genes known to promote M2 activation^56^ (Supplementary Table 1), and identified 3 candidates (Fig. 5e): *Socs2*, *Socs3* and *Rgs1* (Fig. 5f). Among these, *Socs3* exhibited the most pronounced upregulation following *Mettl3* knockdown (Fig. 5g). MeRIP‑qPCR confirmed a significant decrease in m^6^A methylation on *Socs3* mRNA upon *Mettl3* knockdown (Fig. 5h). Subsequently, an RNA degradation assay demonstrated that *Mettl3* knockdown markedly increased *Socs3* mRNA stability, leading to its accumulation (Fig. 5i). These data suggest that *Socs3* mRNA is a target of METTL3-mediated m^6^A modification.

SOCS3 is a feedback regulator of the JAK-STAT signaling cascade^57^. It plays a critical role in constraining the activation of macrophages and microglia, primarily by serving as a key negative regulator of the JAK2-STAT3 pathway^19^. Corroborating this inhibitory function, gene set variation analysis (GSVA) revealed significant attenuation of STAT3 pathway activity following METTL3 knockdown (Suppl. Fig. 8). This bioinformatic evidence supported a mechanism wherein METTL3 silencing relieved the m^6^A-mediated repression of SOCS3, leading to its accumulation and the subsequent suppression of JAK2-STAT3 signaling pathway. We experimentally validated this upstream regulation by demonstrating that siMETTL3-hNVs treatment markedly elevated SOCS3 protein levels and, in parallel, suppressed JAK2 and STAT3 phosphorylation (Fig. 5j). Collectively, these findings delineated a coherent METTL3–m^6^A–SOCS3–JAK2/STAT3 regulatory axis. Mechanistically, METTL3 silencing stabilized SOCS3 mRNA and increased SOCS3 protein expression. This was associated with reduced JAK2-STAT3 phosphorylation, contributing to the attenuation of the pro-inflammatory phenotype.

### siMETTL3-hNVs confer cognitive recovery in RIBI by reprogramming the inflammatory microenvironment

**Fig. 6:**
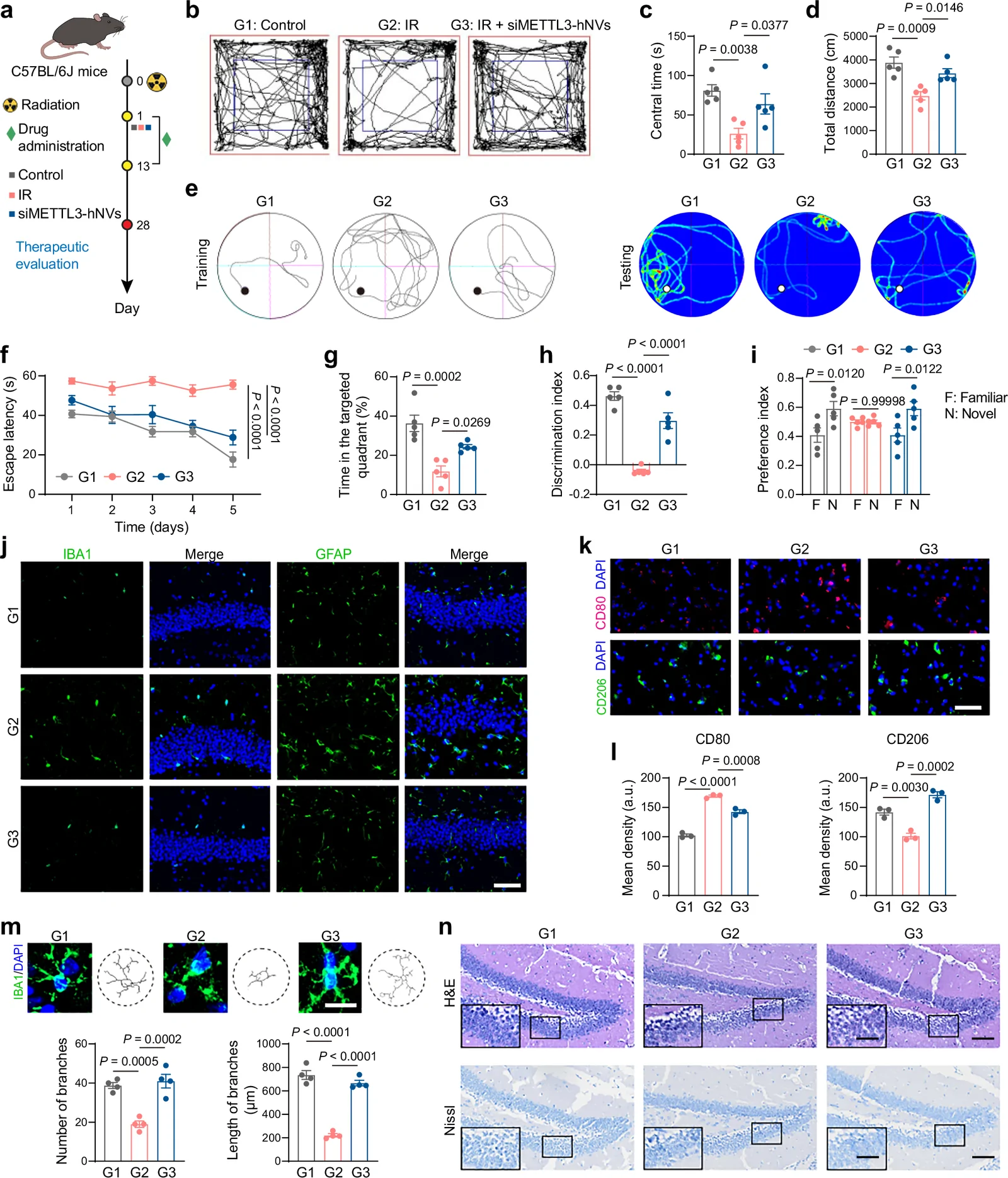
Therapeutic effects of siMETTL3-hNVs in a RIBI mouse model. **a** Experimental design for siMETTL3-hNVs administration in the radiation-induced brain injury mouse model. Created in BioRender. Xu, L. (2026) https://BioRender.com/0qdokxk. **b**–**d** Movement paths in the open-field test (**b**) and quantification of central time (**c**) and total distance (**d**) (*n* = 5 biologically independent mice). **e–g** Video tracking images and heatmaps in the Morris water maze test (**e**), escape latency over the five-day training course (**f**) and time spent in the target zone during the probe trial (**g**) (*n* = 5 biologically independent mice; exact *P* values for **f**: G1 vs. G2 *P* = 7.03E − 11; G2 vs. G3 P = 4.48E − 7). **h**, **i** Discrimination index (**h**) and preference index (**i**) in the novel object recognition test (*n* = 5 biologically independent mice; exact *P*-values for (**h**): G1 vs. G2 *P* = 9.72E − 7; G2 vs. G3 *P* = 5.73E − 5). **j** Immunofluorescence images of IBA-1 and GFAP in the hippocampus after different treatments (*n* = 3 biologically independent brain samples). Scale bar, 100 μm. **k**, **l** Immunofluorescence images (**k**) and quantification (**l**) of CD80^+^ and CD206^+^ microglia in brain sections (*n* = 3 biologically independent brain samples; CD80^+^ exact *P*-value: G1 vs. G2 *P* = 4.01E − 6). Scale bar, 40 μm. **m** Magnified images of IBA-1^+^ microglia and corresponding skeletonized traces, with quantification of branch number and branch length (*n* = 4 biologically independent brain samples; exact *P* values for branch length: G2 vs. G1 *P* = 5.46E − 7; G2 vs. G3 *P* = 1.89E − 6). Scale bars, 10 μm. **n** Haematoxylin and eosin and Nissl staining of hippocampal sections after different treatments (*n* = 3 biologically independent brain samples). Scale bars, 100 μm for the main images and 20 μm for the insets. G1, Control; G2, irradiation; G3, irradiation + siMETTL3-hNVs. All experiments in this figure were performed using six-week-old female C57BL/6J mice. For (**c**, **d**, **f**–**i**, **l**, **m**), data are presented as mean ± s.e.m., with statistical significance assessed using one-way ANOVA followed by Tukey’s multiple comparison test (**c**, **d**, **g**, **h**, **l**, **m**) or two-way ANOVA (**f**, **i**). Source data are provided as a Source Data file.

RIBI is a severe condition affecting 20-50% of patients undergoing cranial radiotherapy, characterized by progressive cognitive decline in the absence of effective treatments^58^. Given that microglial dysfunction is a key driver of RIBI pathogenesis^39,59^, we evaluated the therapeutic potential of siMETTL3-hNVs in a mouse model of this condition (Fig. 6a). The RIBI model was established by administering 30 Gy whole-brain irradiation to six-week-old female C57BL/6J mice. Body weight was monitored every other day thereafter. siMETTL3-hNVs treatment attenuated irradiation-induced body weight loss in mice (Suppl. Fig. 9a). siMETTL3-hNVs treatment improved behavioral, cognitive, and memory performance in irradiated mice (Fig. 6b–i; Suppl. Fig. 9b–e). In contrast, PBS-treated irradiated mice exhibited pronounced cognitive impairment. Collectively, these data indicate functional improvement after siMETTL3-hNVs treatment in a preclinical model of RIBI.

Glial cell activation is a hallmark pathological feature of radiation-induced brain injury^60^. To assess neuroinflammation, we examined the activation of astrocytes and microglia in the hippocampal region by measuring the expression of GFAP and IBA-1, respectively. In RIBI model mice, siMETTL3-hNVs treatment significantly reduced the activation of both microglia and astrocytes in the hippocampus (Fig. 6j). Given the hippocampus’s critical role in memory and cognition, we next analyzed pro-inflammatory cytokine expression in isolated hippocampal tissue. mRNA levels of *Il1b*, *Il6*, and *Tnf* were significantly elevated after irradiation, and this increase was markedly suppressed by siMETTL3-hNVs treatment (Suppl. Fig. 9f–j). Immunofluorescence and flow cytometry analyses confirmed that siMETTL3-hNVs promoted microglial repolarization from the pro-inflammatory M1 phenotype to the anti-inflammatory M2 phenotype (Fig. 6k, l; Suppl. Fig. 9k, l). Concurrently, increases in microglial branch length and number indicated the restoration of morphological homeostasis (Fig. 6m). Together, these results demonstrated that siMETTL3‑hNVs reduced several neuroinflammatory features in RIBI, including glial hyperactivation and dysregulated cytokine signaling.

To evaluate the neuroprotective efficacy of siMETTL3-hNVs against radiation-induced brain injury (RIBI) in vivo, we conducted histological analyses using H&E and Nissl staining. Histological analysis revealed that siMETTL3-hNVs treatment significantly alleviated neuronal damage, reducing cell shrinkage and nuclear pyknosis while restoring Nissl bodies to normal distribution (Fig. 6n; Suppl. Fig. 9m). Furthermore, histopathological assessment of major organs revealed no significant abnormalities in any treatment group (Suppl. Fig. 10), supporting the favorable biosafety profile of siMETTL3‑hNVs. Together, these results indicated that siMETTL3‑hNVs provide neuroprotective effects against RIBI by preserving neuronal structure and maintaining systemic safety.

## Discussion

This study describes siMETTL3-hNVs, a hybrid nanoplatform that combines epitranscriptomic regulation with immunomodulation for neuroinflammation treatment. The platform enables METTL3 small interfering RNA delivery with preferential uptake by microglia, thereby reducing N6-methyladenosine modification, increasing SOCS3 mRNA stability, and limiting JAK2–STAT3 signaling. This process is associated with microglial repolarization and attenuation of the neuroinflammatory microenvironment. In both lipopolysaccharide-induced and radiation-induced neuroinflammatory mouse models, siMETTL3-hNVs improved cognitive function, reduced neuronal injury and showed a favorable safety profile, supporting further evaluation of this platform for central nervous system inflammatory disorders.

Compared with genome-editing tools such as CRISPR-Cas9, which can induce DNA double-strand breaks and may carry risks of chromosomal deletions or rearrangements^61^, this epitranscriptomic targeting strategy presents a safer alternative. Its inherent reversibility, which allows for the restoration of epigenetic homeostasis upon treatment cessation, is a distinct advantage when long-term silencing safety is unconfirmed or sustained inhibition is unnecessary^62^. Furthermore, the therapeutic scope of epitranscriptomic regulation is broadly extensible, targeting diverse regulators such as DNA methyltransferases, histone deacetylases, and key proteins including TET2, as well as emerging modification types like m^7^G and 5hmC^46,47,63,64^. This versatility supports further investigation of epigenetic intervention as an adaptable therapeutic framework for neuroinflammation.

The siMETTL3-hNVs platform has several features relevant to further development. First, it addresses the poor scalability of natural EVs via a continuous membrane extrusion process amenable to industrial-scale manufacturing, thus enabling reliable, high-throughput production. Furthermore, siMETTL3-hNVs exhibit functional advantages over synthetic carriers by inheriting native membrane proteins that promote cytosolic cargo delivery and confer immune evasion capabilities^65^. The enrichment of functional proteins within lipid raft microdomains provides a structural basis for the spatial organization of bioactive molecules^66^. In addition, the endogenous composition ensures high biocompatibility while reducing immune activation and cytotoxicity, which is essential for chronic treatments requiring repeated administration^32,67^. Together, these features support further optimization and evaluation of this nanovesicle platform.

The efficacy and safety profile of siMETTL3-hNVs observed in acute inflammatory and radiation-induced brain injury models should be considered alongside several limitations. First, this study primarily used acute or injury-associated neuroinflammatory models; therefore, the long-term therapeutic utility of siMETTL3-hNVs in chronic neurodegenerative conditions remains to be established. In addition, the translational relevance of these findings requires further evaluation because of species differences in nanomedicine pharmacokinetics and immune responses. Future work should optimize dosing regimens, assess long-term safety, evaluate efficacy in additional disease models, and clarify manufacturing parameters required for translational development.

## Methods

### Materials

The following reagents were obtained commercially: siMETTL3 (GenePharma, Shanghai, China); standard laboratory reagents, including lysis buffer, antibody diluent, protease inhibitor cocktail, antigen retrieval solution, and bovine serum albumin (BSA) (Sangon Biotech, Shanghai, China); primary and secondary antibodies for immunoassays (Abmart, Shanghai, China); an Annexin V-FITC/PI apoptosis detection kit (Beyotime Biotechnology, Shanghai, China); ELISA kits (Dakewe Biotech, Shenzhen, China); and all primers (Tsingke Biotech, Beijing, China). All other chemicals were purchased from Sigma-Aldrich (USA).

### Animals

Female C57BL/6J mice (6 weeks old) were purchased from GemPharmatech (Jiangsu, China). Mice were housed under specific pathogen-free (SPF) conditions on a 12 h light/dark cycle with ad libitum access to food and water. Only female mice were used in this study; therefore, sex was not included as a biological variable in the statistical analysis. All animal experiments were approved by the Institutional Animal Care and Use Committee of Shenzhen Bay Laboratory (Approval No. AERL202403) and were conducted in accordance with the NIH Guide for the Care and Use of Laboratory Animals.

### Cells

Cell lines (BV2, HT22, C8-D1A, and bEnd.3) were obtained from the American Type Culture Collection (ATCC). Cells were cultured in Dulbecco’s Modified Eagle’s Medium (DMEM) supplemented with 10% fetal bovine serum (FBS) and 1% penicillin-streptomycin at 37 °C in a humidified atmosphere containing 5% CO₂. Cells were routinely passaged to maintain confluence and viability.

### Bioinformatics analysis

The raw sequencing data for dataset GSE264486 were obtained from the Gene Expression Omnibus (GEO) database. Raw RNA-seq reads were quality-assessed using FastQC (v0.11.9). Adapter sequences and low-quality bases were trimmed using Trimmomatic (v0.39) to obtain high-quality clean reads. The clean reads were aligned to the GRCm38/mm10 reference genome with HISAT2 (v2.2.1). The resulting alignments were processed with SAMtools (v1.12) to convert SAM files to BAM format, sort them, and generate indexes. Peak calling was performed on the aligned reads using exomePeak2 (v2.0.0) with default parameters to identify significantly enriched regions (peaks). Motif analysis of the identified peaks was carried out using HOMER (v4.11) to uncover potential regulatory motifs. Finally, the Integrative Genomics Viewer (IGV; v2.12.3) was used to visualize read coverage and peak annotations across specific genomic loci.

### Preparation and characterization of single NVs

Preparation of M2-NVs: BV2 microglial cells were first polarized to the M2 phenotype by stimulation with 20 ng/mL IL-4 (Sigma-Aldrich) for 48 h. The polarized cells were then resuspended in a hypotonic lysis buffer and mechanically disrupted with a Dounce homogenizer. The resulting lysate was centrifuged at 3,200 × g for 5 min to remove nuclei and cellular debris. The supernatant was collected, carefully layered onto a pre-established discontinuous OptiPrep density gradient (10% and 50%), and ultracentrifuged at 100,000 × g for 2 h using an LE-80K ultracentrifuge (Beckman Coulter). The fraction at the interface between the gradient layers, which contained the M2-NVs, was collected. This fraction was washed *via* a second ultracentrifugation step (100,000 × g, 2 h) and finally resuspended in an appropriate buffer for subsequent use. Preparation of siMETTL3@Lipo: siMETTL3@Lipo were prepared using the thin-film hydration and extrusion method. Briefly, a lipid mixture comprising DOTAP, DOPE, DSPE-PEG2000, and cholesterol at a molar ratio of 20:24:5:1 was dissolved in chloroform. The chloroform was removed by rotary evaporation at room temperature to form a thin, homogeneous lipid film. This film was then hydrated with sterile water for injections. The resulting suspension was extruded through a polycarbonate membrane using an Avanti Hand Extruder (Avanti Polar Lipids) to generate blank liposomes. The final product, siMETTL3@Lipo, was obtained by incubating the blank liposomes with 20 μM METTL3-siRNA at 37 °C for 15 min to facilitate siRNA loading.

### Preparation of siMETTL3-hNVs

To prepare siMETTL3-hNVs, M2‑NVs and siMETTL3@Lipo were mixed at a 1:1 protein-to-lipid weight ratio. The mixture was sonicated for 5 min and then extruded through a 100 nm polycarbonate membrane using a mini extruder. Protein content in the resulting siMETTL3‑hNVs pellet and the supernatant was quantified using the Bradford assay. Assessment of hydrodynamic diameter and zeta potential during synthesis was carried out with a DLS instrument (Malvern Instruments Nano-ZS90). Morphology was characterized by TEM (Hitachi HT7800). To assess membrane fusion, M2‑NVs and siMETTL3@Lipo were labeled with the lipophilic fluorescent dyes DiD (red) and DiO (3,3’-dioctadecyloxacarbocyanine perchlorate, green), respectively. Successful fusion was confirmed by two complementary methods: visualizing dye colocalization using confocal laser scanning microscopy (CLSM; ZEISS LSM980) and quantifying FRET.

### qRT-PCR analysis

Total RNA was extracted from tissue or cell samples using a commercial RNA extraction kit (Vazyme Biotech, Nanjing, China). cDNA was synthesized from the extracted RNA by reverse transcription using HiScript II Q RT SuperMix (Vazyme Biotech) according to the manufacturer’s instructions. qRT-PCR amplifications were performed in a 20 μL reaction mixture containing cDNA and SYBR Green Master Mix (Vazyme Biotech). Each sample was assayed in triplicate on a QuantStudio 5 Real-Time PCR System (Applied Biosystems, USA). The primer sequences used are listed in Suppl. Table 2.

### SDS-PAGE and Western blot

Proteins were extracted from M2 cells, M2-NVs, siMETTL3@Lipo, and siMETTL3-hNVs using RIPA lysis buffer supplemented with protease and phosphatase inhibitors. For electrophoretic separation, 20 μg of protein per sample was denatured and separated on 10% SDS-polyacrylamide gels. For Coomassie blue staining, the gels were fixed, stained, and subsequently destained. For Western blot, proteins were transferred onto polyvinylidene difluoride (PVDF) membranes. The membranes were then blocked with 5% BSA. The membranes were then incubated sequentially with designated primary antibodies and a corresponding horseradish peroxidase (HRP)-conjugated secondary antibody.

### Cytokine binding quantification

To assess cytokine binding activity, 100 µL of particle suspensions at serial concentrations were incubated with an equal volume of PBS containing 1 ng each of IL-1β, IL-6, and TNF-α at 37 °C for 2 h. After centrifugation (15,000 × g, 15 min) to pellet the particles, cytokine concentrations in the supernatant were measured using ELISA kits according to the manufacturer’s instructions.

### siRNA release kinetics

The siRNA release profile was determined using a centrifugal ultrafiltration method. Purified nanoparticles were incubated at 37 °C with continuous agitation in PBS at pH 7.4 or 5.5. At predetermined time intervals, aliquots of the release medium were collected. The concentration of released fluorescent siRNA in the aliquots was measured against a standard curve to calculate the cumulative release percentage.

### In vitro cellular uptake

To analyze cellular uptake, BV2, C8-D1A, HT22, and bEnd.3 cells seeded in 24-well plates were incubated with DiD-labeled siMETTL3-hNVs for 30 or 90 min. Cellular uptake was quantified by flow cytometry (CytoFLEX S, Beckman Coulter). To evaluate uptake under inflammatory conditions, BV2 cells were seeded at a density of 5 × 10⁵ cells per well and pre-treated with LPS (100 ng/mL) for 24 h to induce an M1-like polarization state. The pre-treated cells were then incubated with DiD-labeled siMETTL3-hNVs. Nanoparticle internalization was quantified by flow cytometry at the indicated time points.

### Endolysosomal escape assay

To track nanoparticle endolysosomal escape, BV2 cells were incubated with DiD-labeled siMETTL3-hNVs for various durations (0, 4, 6, and 8 h). At each time point, lysosomes and nuclei were stained with LysoTracker Green (50 nM) and Hoechst 33342 (1 μg/mL), respectively. After fixation with 4% paraformaldehyde, fluorescence images were acquired by CLSM. The colocalization of siMETTL3-hNVs with lysosomes was quantified using Mander’s overlap coefficient in ImageJ software. Data were visualized using GraphPad Prism 9.0.

### In vitro cytotoxicity assay

The viability of BV2 and bEnd.3 cells after treatment with nanoparticles was evaluated using the Cell Counting Kit-8 (CCK-8) assay. Briefly, cells seeded in 96-well plates were treated for 24 h with siMETTL3@Lipo, M2-NVs, or siMETTL3-hNVs at a dose of 2 pmol siMETTL3 per well. Then, 10 µL of CCK-8 reagent was added to each well, and the plates were incubated at 37 °C for 2 h. The absorbance at 450 nm was measured using a microplate reader (Tecan, Switzerland). Cell viability was normalized to that of untreated controls.

### Microglial phenotypic switching assay

The ability of nanoparticles to induce microglial phenotypic switching was assessed in BV2 cells. After M1 polarization was induced by treating cells with LPS (100 ng/mL) for 24 h, the cells were incubated with respective nanoparticles for another 24 h. Phenotypes were then analyzed by flow cytometry following immunostaining with antibodies against the M1 marker CD86 and the M2 marker CD206.

### In vitro inhibition of inflammation

BV2 microglial cells were pre-stimulated with LPS (100 ng/mL) for 24 h to induce a pro-inflammatory state. The cells were then treated for 30 min with PBS, siMETTL3@Lipo, M2-NVs, or siMETTL3-hNVs at equal mass concentrations. Subsequently, nanoparticles were removed from the culture medium by centrifugation at 15,000 × g for 15 min. The concentrations of IL-6, TNF-α, IL-4, and IL-10 in the collected supernatant were measured using ELISA.

### Immunofluorescence staining

For immunofluorescence staining, cells grown in confocal dishes were washed with PBS and then fixed with 4% paraformaldehyde (PFA) for 15 min. Cells were then permeabilized with 0.3% Triton X-100 for 30 min, blocked with 10% BSA for 2 h at room temperature (RT), and incubated with primary antibodies against IBA1 (1:1000), MAP2 (1:500), and GFAP (1:1000) overnight at 4 °C. Finally, cells were incubated with fluorescent secondary antibodies for 1 h at RT and counterstained with DAPI. Images were acquired using CLSM.

### Apoptosis assay

Apoptosis was analyzed in a Transwell co-culture system of BV2 and HT22 cells using an Annexin V-FITC/PI apoptosis detection kit (Beyotime, China). After inflammatory priming with LPS (100 ng/mL for 24 h), the co-cultures were treated for 24 h with PBS, siMETTL3@Lipo, M2-NVs, or siMETTL3-hNVs. HT22 cells were then collected, stained following the manufacturer’s protocol, and analyzed by flow cytometry. In parallel, the expression of key apoptosis-related proteins (Bax, Bcl-2, Cleaved Caspase-3) was analyzed by Western blotting.

### m^6^A dot blot assay

Total RNA was extracted using a commercial RNA extraction kit, as described in the methods section. Briefly, denatured RNA samples were serially diluted, spotted onto a nylon membrane, and UV-crosslinked. The membrane was blocked with 5% non-fat milk and then incubated sequentially with an anti-m^6^A antibody and a HRP-conjugated secondary antibody. To verify equal RNA loading, a parallel membrane was stained with 0.02% methylene blue.

### RNA-seq assay and data analysis

RNA-seq was performed to profile the transcriptomes of METTL3-silenced BV2 microglial cells stimulated with LPS (100 ng/mL for 24 h). Following RNA extraction, high-quality total RNA was used to construct sequencing libraries, which were sequenced on an Illumina NovaSeq 6000 platform (Sinotech Genomics, Shanghai, China). Clean reads obtained after quality control and adapter trimming were aligned to the GRCm38 reference genome for gene expression quantification. Differentially expressed genes were identified using thresholds of |log_2_(fold change)| > 0.585 and false discovery rate < 0.05. Functional enrichment was analyzed using KEGG, GSVA, and GSEA. Resulting plots were generated with the ggplot2 package in R.

### Methylated RNA immunoprecipitation (MeRIP)-qPCR

Total RNA was extracted from BV2 cells under experimental and control conditions using TRIzol reagent. Fragmented RNA was subjected to MeRIP using a commercial kit (BersinBio) with anti-m^6^A antibody or control IgG conjugated to magnetic beads. The eluted RNA was analyzed by qRT-PCR on a Roche LightCycler 480 II system using SYBR Green Premix (AG, China) and gene-specific primers flanking the predicted m^6^A peak regions. The m^6^A enrichment was calculated relative to the input RNA and normalized to the IgG control group.

### mRNA stability assay

The decay rates of target mRNAs were assessed in BV2 cells treated with siMETTL3‑hNVs or siNC-hNVs. Transcription was halted by actinomycin D treatment, and total RNA was isolated at serial time points (0, 3, and 6 h) thereafter. The abundance of target transcripts was measured at each time point by qRT‑PCR and normalized to 18S rRNA for data analysis.

### In vitro BBB Transwell model and endocytic inhibition assays

An in vitro BBB model was established by seeding bEnd.3 cells (6 × 10⁴ cells/well) onto Transwell inserts. The model was used for experiments after the TEER reached values exceeding 100 Ω·cm². BV2 microglial cells (1 × 10⁵ cells/well) were cultured in the lower chamber. DiR-labeled nanoparticles were then applied to the upper chamber. The cellular uptake and distribution of nanoparticles within the BBB model were visualized by CLSM after 12 h of incubation, while the extent of transendothelial transport was quantified at 24 h by measuring DiR fluorescence in the lower chamber cells using flow cytometry. To investigate the mechanism of transendothelial transport, bEnd.3 cells were pretreated with MβCD (4.5 mM), CPZ (5 μg/mL), or EIPA (10 μM) for 30 min at 37 °C, respectively, before addition of siMETTL3-hNVs. To assess the contribution of the CCR2–CCL2 axis, siMETTL3-hNVs were pretreated with RS504393 (10 μM) for 30 min at 37 °C before being applied to the upper chamber. Transendothelial transport was then quantified by flow cytometry analysis of lower-chamber BV2 cells.

### CCL2 secretion assay in different brain-relevant cell types

To compare the inflammatory production of CCL2 across different brain-relevant cell types, BV2, bEnd.3, HT22, and C8-D1A cells were cultured under identical conditions and stimulated with LPS (100 ng/mL) for 24 h. Culture supernatants were collected, clarified by centrifugation, and analyzed using a commercial CCL2 ELISA kit according to the manufacturer’s instructions. Cytokine levels were normalized to total cellular protein content determined by BCA assay.

### In vivo biodistribution and brain-cell distribution of siMETTL3-hNVs

For whole-particle biodistribution analysis, siMETTL3-hNVs, M2-NVs, and siMETTL3@Lipo were labeled with DiR and intravenously administered to mice via the tail vein. Neuroinflammation was induced via intraperitoneal (i.p.) injections of LPS (0.25 mg/kg daily for 7 days). Mice were then intravenously injected with free DiR or DiR-labeled siMETTL3@Lipo, M2-NVs, or siMETTL3-hNVs. Whole-body fluorescence was monitored at designated time points using an IVIS Spectrum imaging system (PerkinElmer). Finally, major organs and brains were collected for ex vivo fluorescence imaging. For cargo-distribution analysis at the cellular level, siMETTL3-hNVs were formulated using Alexa Fluor 647-labeled siRNA and administered via a single intravenous injection. At 12 h post-injection, brains were harvested, dissociated into single-cell suspensions, and analyzed by flow cytometry to determine the percentage of Alexa Fluor 647-positive cells in different brain cell populations. Brain-cell flow cytometry was performed using the following panel: Live/Dead (BV510), CD45 (PerCP-Cy5.5), CD11b (FITC), CD31 (BV421), ACSA-2 (PE), NeuN (Vio 780), and Alexa Fluor 647-labeled siRNA (AF647).

### In vivo inhibitor validation and inflammation-dependent brain delivery studies

For inflammation-dependent brain delivery studies, mice were assigned to a healthy control group or to neuroinflammatory groups treated with LPS at 0.1, 0.25, 0.5, or 1 mg/kg by intraperitoneal injection once daily for 7 consecutive days. Fluorescently labeled siMETTL3-hNVs were then intravenously administered, and brain accumulation was quantified by IVIS imaging. Brain tissues were collected for CCL2 ELISA. For in vivo inhibitor validation, mice received an intraperitoneal injection of MβCD (100 mg/kg) 1 h before intravenous administration of DiR-labeled siMETTL3-hNVs. In separate experiments, siMETTL3-hNVs were pretreated with RS504393 before injection. Brain fluorescence was quantified by IVIS imaging at 12 h post-injection, followed by ex vivo IVIS imaging of isolated brains.

### Evans blue assay for BBB permeability

BBB integrity in the LPS-induced neuroinflammatory model was assessed using an Evans blue extravasation assay. Briefly, mice were intravenously injected with 2% Evans blue solution through the tail vein. After circulation for 4 h, mice were deeply anesthetized and perfused with phosphate-buffered saline to remove intravascular dye. Brains were harvested, photographed, and homogenized in formamide. Evans blue was extracted at 55 °C, and dye content was quantified by measuring absorbance at 620 nm or fluorescence using the indicated excitation/emission settings.

### Neuroinflammatory mouse models and treatments

Two distinct neuroinflammatory mouse models were established: (1) an LPS-induced model, created by daily intraperitoneal injections of LPS (0.25 mg/kg) for 7 consecutive days; and (2) a RIBI model, generated by administering a single-fraction whole-brain irradiation dose of 30 Gy at a rate of 3 Gy/min. Starting on the first day of model establishment, mice received intravenous injections of saline, siMETTL3@Lipo, M2-NVs, or siMETTL3-hNVs every other day. Upon treatment completion, brain tissues were harvested for analysis. Microglial polarization was analyzed by immunofluorescence staining for the M1 marker CD80 and the M2 marker CD206. Neuroprotective effects were assessed through hematoxylin and eosin (H&E) and Nissl staining. Immune cell infiltration was quantified by flow cytometry. For brain immune cell isolation, freshly collected brain tissues were mechanically dissociated into single-cell suspensions and passed through a cell strainer. Myelin and debris were removed by centrifugation using a 30% Percoll gradient. Cells collected from the pellet were washed with staining buffer and used for subsequent flow cytometry analysis: Live/Dead (BV510), CD45 (PerCP-Cy5.5), CD11b (FITC), CD80 (BV421), and CD206 (AF647). Cytokine levels in brain tissue homogenates were measured using ELISA.

### Neurobehavioral tests

Open-field test: Mice were individually placed in a cubic arena (60 × 60 × 60 cm) and allowed to explore freely for 10 min. Their movement was digitally tracked. Locomotor activity and anxiety-like behavior were quantified by measuring the total distance traveled, average speed, and the time spent in the central zone. The arena was thoroughly cleaned between trials. Novel object recognition test: Mice were first habituated to an open-field arena (40 × 40 cm). During the training phase, they were exposed to two identical objects for 5 min. After a 1 h retention interval, one familiar object was replaced with a novel object for a 5 min test session. Recognition memory was evaluated by calculating a discrimination index based on the exploration time of the novel and familiar objects. The arena and objects were cleaned with 75% ethanol between trials to eliminate olfactory cues. Morris water maze test: Mice were trained over five consecutive days (two trials per day) to locate a submerged platform in a circular pool filled with water maintained at 23 ± 1 °C. Twenty-four hours after the last training session, a 60 s probe trial was conducted with the platform removed. Spatial memory retention was assessed by measuring the time spent in the target quadrant and the number of platform-location crossings.

### Safety evaluation

Hemocompatibility was evaluated using a standard hemolysis assay. Specifically, mouse red blood cells were isolated and incubated with serial concentrations of siMETTL3@Lipo, M2-NVs, or siMETTL3-hNVs for 1 h at 37 °C, followed by centrifugation. The absorbance of the supernatant was measured at 570 nm. The hemolysis percentage was calculated relative to the saline (negative control) and distilled water (100% hemolysis, positive control) groups. For in vivo biosafety assessment, major organs (e.g., heart, liver, spleen, lungs, and kidneys) were harvested from euthanized mice at the endpoint of the treatment regimen.

### Statistics and reproducibility

Data processing, statistical analysis, and figure generation were performed using GraphPad Prism 9.5, Fiji/ImageJ 1.54p, FlowJo v10.8.1, Living Image 4.8.4, and R 4.4.3. Bioinformatics tools and package versions are listed in the relevant Methods sections. All experiments have been reproduced at least three times, and all attempts at replication were successful with self-consistent results. Data are presented as mean ± standard error of the mean (s.e.m), unless otherwise stated. Differences between two groups were assessed using an unpaired two-tailed Student’s t-test unless otherwise stated. For comparisons among multiple groups, one-way or two-way analysis of variance (ANOVA) was applied, as appropriate, followed by Tukey’s post hoc test for multiple comparisons. For RNA-seq analysis, differential expression was assessed using limma-voom followed by empirical Bayes moderated t-test with Benjamini–Hochberg adjustment. A *P* value of less than 0.05 was considered statistically significant.

### Reporting summary

Further information on research design is available in the Nature Portfolio Reporting Summary linked to this article.

## Supplementary information

Supplementary Information

Reporting Summary

Transparent Peer Review file

## Source data

Source data

## Data Availability

All data generated or analyzed during this study are available within the article, Supplementary Information, or Source Data file. The RNA-seq data generated in this study have been deposited in the Sequence Read Archive under accession number PRJNA1400104. The public m6A-seq dataset analyzed in this study is available from the Gene Expression Omnibus under accession number GSE264486. Source data are provided with this paper.

## Code availability

The R and shell scripts used for the sequencing data analysis have been deposited in Zenodo^68^ and are available at https://doi.org/10.5281/zenodo.20627531.
