# Supplementary material for: Targeting m6A writer METTL3 with engineered nanovesicles reduces neuroinflammation in vitro and in vivo
Source: Nat Commun. 2026 Aug 8;17:9534. doi: 10.1038/s41467-026-75862-4 (PMC13547405; doi:10.1038/s41467-026-75862-4)
Supplement: Supplementary file 1 [file 41467_2026_75862_MOESM1_ESM.pdf]

Supplementary Materials

Targeting m^{6}A writer METTL3 with engineered nanovesicles reduces neuroinflammation in vitro and in vivo

Liangfu Xu (徐良富)[1,2,3], Yuanwei Pan (潘远伟)[3], Guanjun Li (李冠俊)[1,2], Peng She (佘鹏)[4], Qian-Fang Meng (孟倩芳)[3,*], Zhigang Liu (刘志刚)[1,2,*], and Lang Rao (饶浪)[3,*]

[1] Cancer Center, Dongguan Key Laboratory of Precision Diagnosis and Treatment for Tumors, The Tenth Affiliated Hospital, Southern Medical University (Dongguan People's Hospital), Dongguan 523059, China.

[2] Shenzhen School of Clinical Medicine, Southern Medical University, Shenzhen 518101, China.

[3] Institute of Chemical Biology, Shenzhen Bay Laboratory, Shenzhen 518132, China.

[4] Department of Orthopedics, The Seventh Affiliated Hospital of Sun Yat-Sen University, Shenzhen 518000, China.

* Corresponding e-mail: mengqf@szbl.ac.cn (Q.-F.M.), zhigangliu1983@hotmail.com (Z.L.), and lrao@szbl.ac.cn (L.R.).

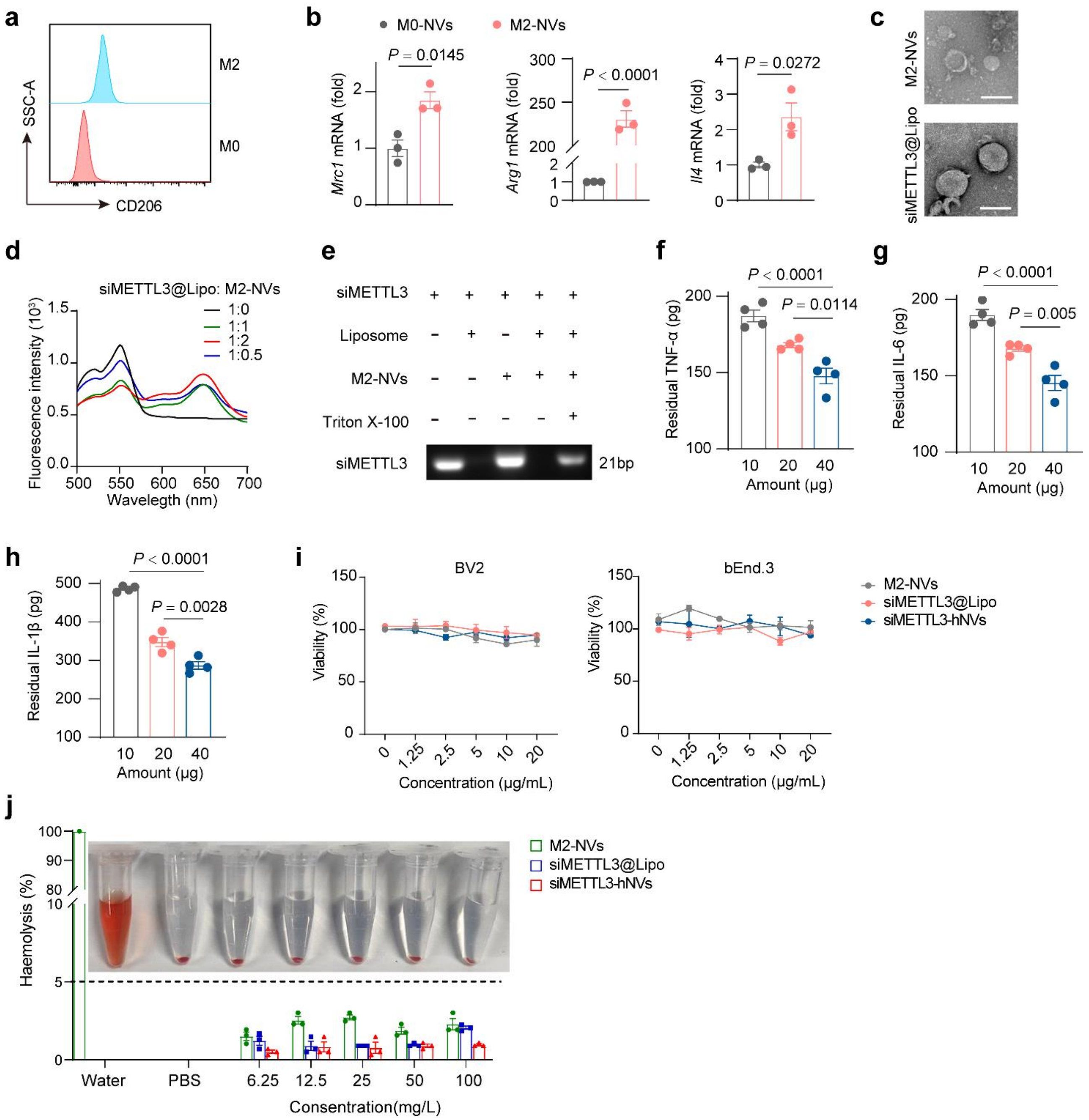

Supplementary Fig. 1 | Preparation and characterization of siMETTL3-hNVs. a, Flow cytometry analysis of CD206 expression in BV2 microglial cells after IL-4 stimulation to induce an M2-polarized phenotype (n = 3 independent experiments). **b,** Relative mRNA expression of *Mrc1*, *Arg1* and *Il4* in M0-NVs and M2-NVs (n = 3 independent experiments; exact P value: *Arg1*, M0-NVs vs. M2-NVs P = 1.72E−5). **c,** TEM images of M2-NVs and siMETTL3@Lipo (n = 3 independent experiments). Scale bar, 100 nm. **d,** Fluorescence changes of DiD-doped siMETTL3@Lipo after mixing with M2-NVs at different mass ratios, as assessed by Förster resonance energy transfer. **e,** Effect of different formulation components on the encapsulation efficiency of siMETTL3, assessed by agarose gel electrophoresis. **f–h,** Binding capacity of M2-NVs toward TNF-α (**f**), IL-6 (**g**) and IL-1β (**h**) (n = 4 independent experiments; exact P values: **f**, 10 vs. 40 μg P = 1.21E−4; **g**, 10 vs. 40 μg P = 3.62E−5; **h**, 10 vs. 40 μg P = 2.33E−7). **i,** *In vitro* cytotoxicity of M2-NVs, siMETTL3@Lipo and siMETTL3-

hNVs at different concentrations in bEnd.3 and BV2 cells (n = 3 independent experiments). **j,** Haemolysis assay of red blood cells incubated with M2-NVs, siMETTL3@Lipo or siMETTL3-hNVs; the black dashed line indicates the haemolysis threshold of 5% (n = 3 independent experiments). For **b** and **f–j**, data are presented as mean ± s.e.m., with statistical significance assessed using two-tailed Student's t-test (**b**) and one-way ANOVA followed by Tukey's multiple comparison test (**f–h**).

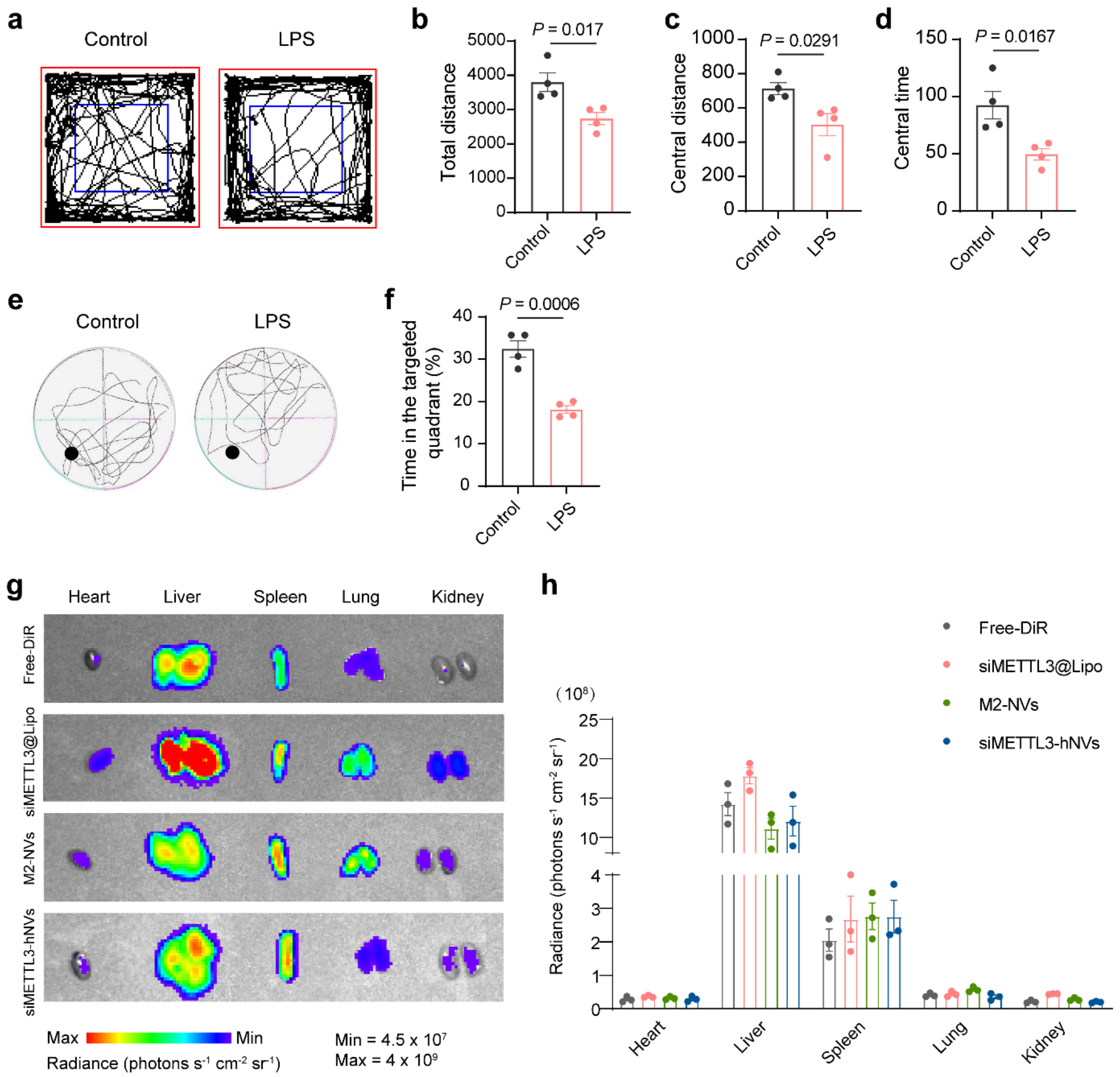

Supplementary Fig. 2 | Validation of the lipopolysaccharide-induced neuroinflammation model and biodistribution of siMETTL3-hNVs. a–d, Representative movement paths of mice in the open-field test (**a**) and quantitative analysis of total distance (**b**), centre distance (**c**) and centre time (**d**) (*n* = 4 biologically independent mice). **e, f** Representative video tracking images in the Morris water maze test (**e**) and quantification of time spent in the target zone during the probe trial (**f**) (*n* = 4 biologically independent mice). **g, h** *Ex vivo* fluorescence images (**g**) and corresponding radiance quantification (**h**) of isolated tissues collected 24 h after administration of the indicated preparations (*n* = 3 biologically independent mice). Experiments were performed using six-week-old female C57BL/6J mice. For **b–d**, **f** and **h**, data are presented as mean ± s.e.m., with statistical significance assessed using a two-tailed Student's t-test (**b–d**, **f**).

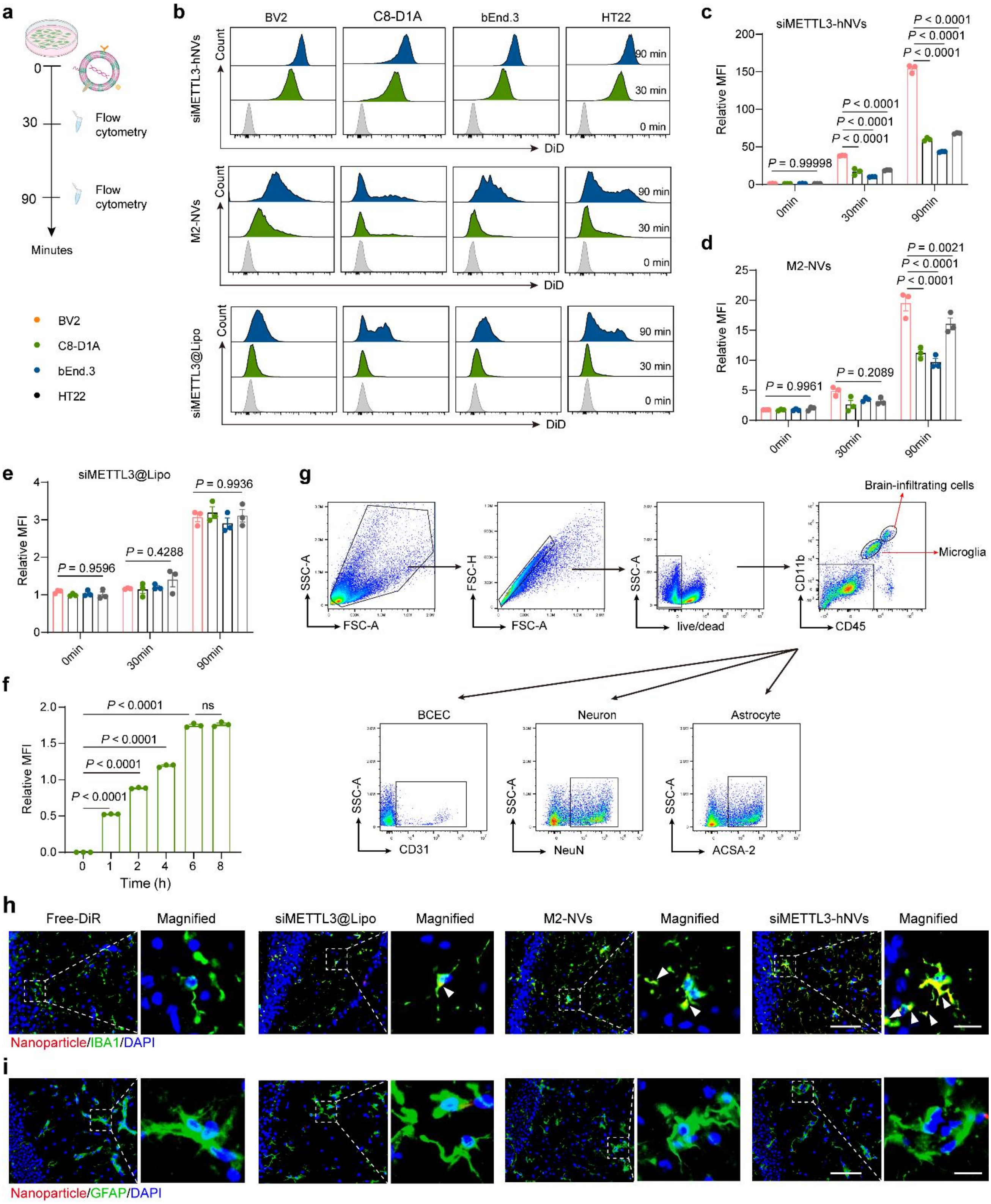

Supplementary Fig. 3 | Cell-type-dependent uptake of siMETTL3-hNVs *in vitro* and *in vivo*. a, Schematic illustration of cellular uptake of siMETTL3-hNVs by different brain-relevant cell types. Created in BioRender. Xu, L. (2026) https://BioRender.com/42o0l20. **b,** Flow cytometry analysis of siMETTL3-hNVs, M2-NVs and siMETTL3@Lipo uptake by BV2 microglia, C8-D1A astrocytes, HT22 neurons and bEnd.3 endothelial cells after 30 or 90 min of incubation (n = 3 independent

experiments). **c–e,** Quantitative analysis of mean fluorescence intensity (MFI) in the indicated cell types after treatment with siMETTL3-hNVs (**c**), M2-NVs (**d**) or siMETTL3@Lipo (**e**) (*n* = 3 independent experiments; exact *P* values of **c**: 30 min, BV2 vs. C8-D1A *P* = 5.47E−11, BV2 vs. bEnd.3 *P* = 1.43E−13, BV2 vs. HT22 *P* = 3.93E−10; 90 min, BV2 vs. C8-D1A *P* = 1.05E−25, BV2 vs. bEnd.3 *P* = 2.19E−27, BV2 vs. HT22 *P* = 8.93E−25; exact *P* values of **d**: 90 min, BV2 vs. C8-D1A *P* = 3.12E−9, BV2 vs. bEnd.3 *P* = 1.03E−10). **f,** Flow cytometric quantification of BV2 uptake after incubation with DiD-labelled siMETTL3-hNVs for the indicated durations (*n* = 3 independent experiments; exact *P* values: 0 vs. 1 h *P* = 8.87E−13; 0 vs. 2 h *P* = 1.73E−15; 0 vs. 4 h *P* = 4.83E−17; 0 vs. 6 h *P* = 4.91E−19; 0 vs. 8 h *P* = 4.43E−19). **g,** Gating strategy for flow cytometry analysis of brain cell populations. Markers used for gating included NeuN for neurons, ACSA-2 for astrocytes, CD11b for microglia and CD31 for brain capillary endothelial cells. **h, i** Immunofluorescence images of brain sections collected 24 h after intravenous administration of Free-DiR or the indicated DiR-labelled formulations and stained for IBA1 (**h**) or GFAP (**i**) (*n* = 3 biologically independent mice). Scale bars, 100 μm (main images) and 25 μm (enlarged views). Experiments were performed using six-week-old female C57BL/6J mice. For **c–f,** data are presented as mean ± s.e.m., with statistical significance assessed using one-way ANOVA (**f**) or two-way ANOVA (**c**, **d**, and **e**) followed by Tukey's multiple comparison test.

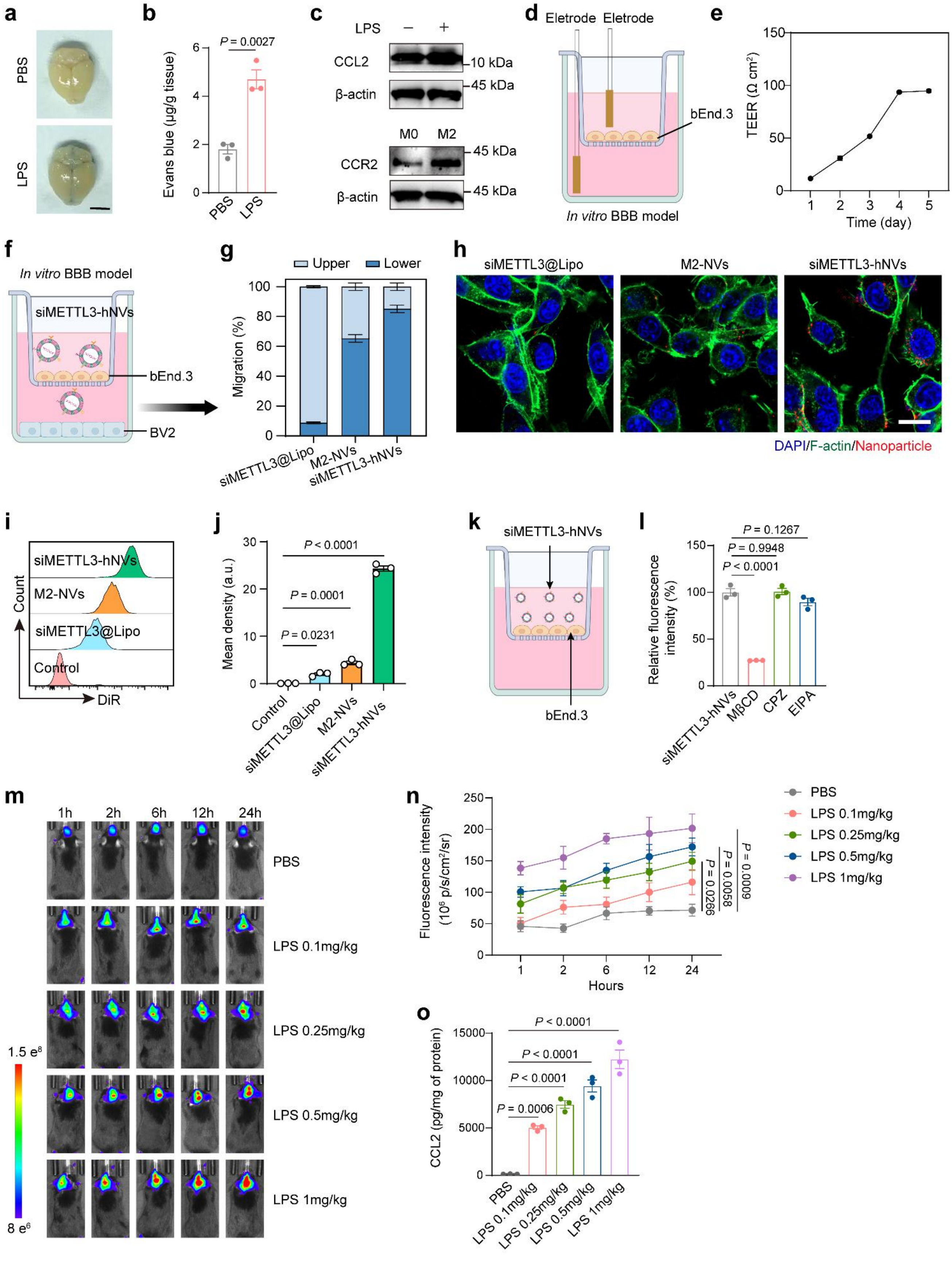

Supplementary Fig. 4 | Mechanistic evaluation of inflamed-brain delivery. a, b Representative images (**a**) and quantification (**b**) of Evans blue extravasation in brain tissues from PBS- or LPS-

injected mice (*n* = 3 biologically independent mice). Scale bars, 0.5 cm. **c,** Western blot analysis of CCL2 expression in LPS-stimulated BV2 cells and CCR2 expression in M2-BV2 cells. **d, e** Schematic illustration of endothelial barrier tightness measurement by transendothelial electrical resistance (TEER) assay (**d**) and TEER values of bEnd.3 endothelial monolayers over culture time (**e**; *n* = 3 independent experiments). **f, g** Schematic of the in vitro BBB model (**f**) and quantification of fluorescence intensity in the BBB model (**g**; *n* = 3 independent experiments). **h,** CLSM images of lower-chamber BV2 cells after treatment with DiD-labelled siMETTL3@Lipo, M2-NVs or siMETTL3-hNVs (*n* = 3 independent experiments). Scale bars, 20 μm. **i, j** Flow cytometry histograms (**i**) and quantitative analysis (**j**) of nanoparticle uptake by BV2 cells in the lower chamber after transendothelial transport (*n* = 3 independent experiments; exact *P* values: Control vs. M2-NVs *P* = 1.39E−4; Control vs. siMETTL3-hNVs *P* = 2.06E−10). **k, l** Schematic of the bEnd.3 Transwell assay for endocytic inhibition (**k**) and relative fluorescence intensity of siMETTL3-hNVs after pretreatment with different endocytic inhibitors, normalized to the siMETTL3-hNVs group (**l**; *n* = 3 independent experiments; exact *P* value: siMETTL3-hNVs vs. MβCD *P* = 4.17E−6). **m, n** *In vivo* fluorescence images (**m**) and quantification of brain fluorescence intensity (**n**) in healthy mice and LPS-induced neuroinflammatory mice receiving PBS or siMETTL3-hNVs (*n* = 3 biologically independent mice). **o,** ELISA analysis of CCL2 levels in brain tissues under different inflammatory conditions (*n* = 3 biologically independent mice; exact *P* values: PBS vs. LPS 0.25 mg/kg *P* = 1.06E−5; PBS vs. LPS 0.5 mg/kg *P* = 1.08E−6; PBS vs. LPS 1 mg/kg *P* = 8.24E−8). Experiments were performed using six-week-old female C57BL/6J mice. For **b**, **e**, **g**, **j**, **l**, **n** and **o**, data are presented as mean ± s.e.m., with statistical significance assessed using two-tailed Student's t-test (**b**) and one-way ANOVA (**j**, **l** and **o**) or two-way ANOVA (**n**) followed by Tukey's multiple comparison test. Panels **d**, **f**, and **k** created in BioRender. Xu, L. (2026) https://BioRender.com/of7bkyt.

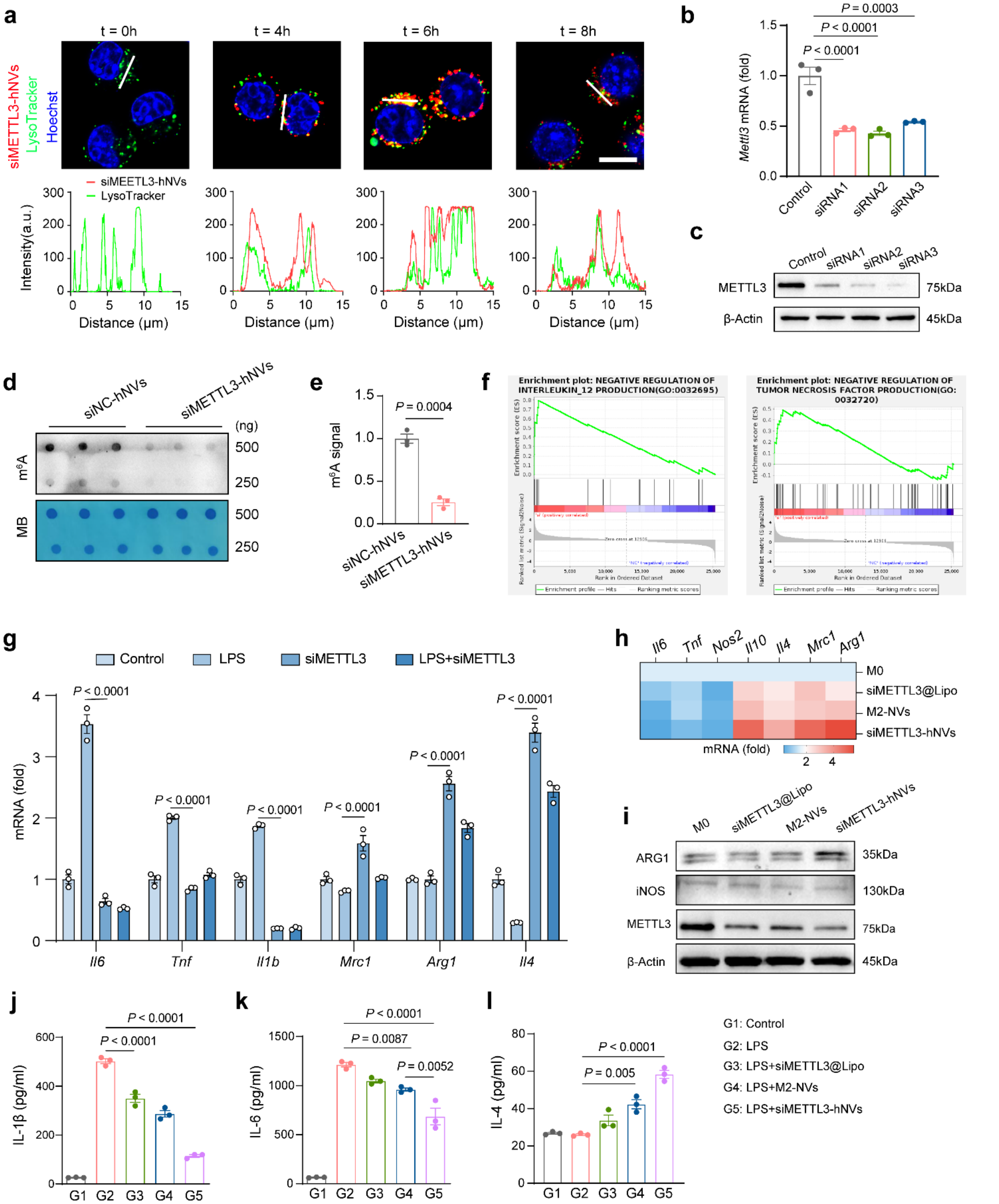

Supplementary Fig. 5 | Intracellular delivery, METTL3 silencing and microglial reprogramming. a, CLSM images of BV2 cells treated with DiD-labelled siMETTL3-hNVs (50 μg/mL) for different durations. Lysosomes were stained with LysoTracker Green (green), and nuclei were stained with Hoechst 33342 (blue) (n = 3 independent experiments). Scale bar, 10 μm. **b, c** qRT-PCR analysis (**b**) and western blot analysis (**c**) of METTL3 expression in LPS-stimulated BV2 cells treated with

different siRNA sequences targeting METTL3 (n = 3 independent experiments for **b**; exact P values: Control vs. siRNA1 P = 8.54E−5; Control vs. siRNA2 P = 5.64E−5). **d, e** Dot blot analysis (**d**) and quantification (**e**) of m^6A levels in BV2 cells. Methylene blue staining served as the loading control (n = 3 independent experiments). **f,** GSEA plots showing enrichment profiles of upregulated genes. **g,** qRT-PCR analysis of inflammatory and polarization-associated genes in LPS-treated BV2 cells with or without Mettl3 knockdown (n = 3 independent experiments; exact P values: *Il6* P = 2.79E−31; *Tnf* P = 6.55E−15; *Il1b* P = 6.72E−21; *Mrc1* P = 2.45E−9; *Arg1* P = 1.06E−19; *Il4* P = 1.16E−32). **h, i** qRT-PCR analysis of M1 marker genes (*Il6*, *Tnf* and *Nos2*) and M2 marker genes (*Arg1*, *Mrc1*, *Il10* and *Il4*) (**h**) and immunoblot analysis of ARG1, iNOS and METTL3 (**i**) in BV2 cells after different treatments (n = 3 independent experiments for **h**). **j–l,** ELISA analysis of IL-1β (**j**), IL-6 (**k**) and IL-4 (**l**) secreted by BV2 cells after different treatments (n = 3 independent experiments; exact P values: **j,** G2 vs. G3 P = 1.35E−5, G2 vs. G5 P = 1.75E−9; **k,** G2 vs. G5 P = 2.57E−5; **l,** G2 vs. G5 P = 6.62E−7). G1, Control; G2, LPS; G3, LPS + siMETTL3@Lipo; G4, LPS + M2-NVs; G5, LPS + siMETTL3-hNVs. For **b**, **e**, **g** and **j–l**, data are presented as mean ± s.e.m., with statistical significance assessed using two-tailed Student's t-test (**e**) and one-way ANOVA (**b**, **j-l**) or two-way ANOVA (**g**) followed by Tukey's multiple comparison test.

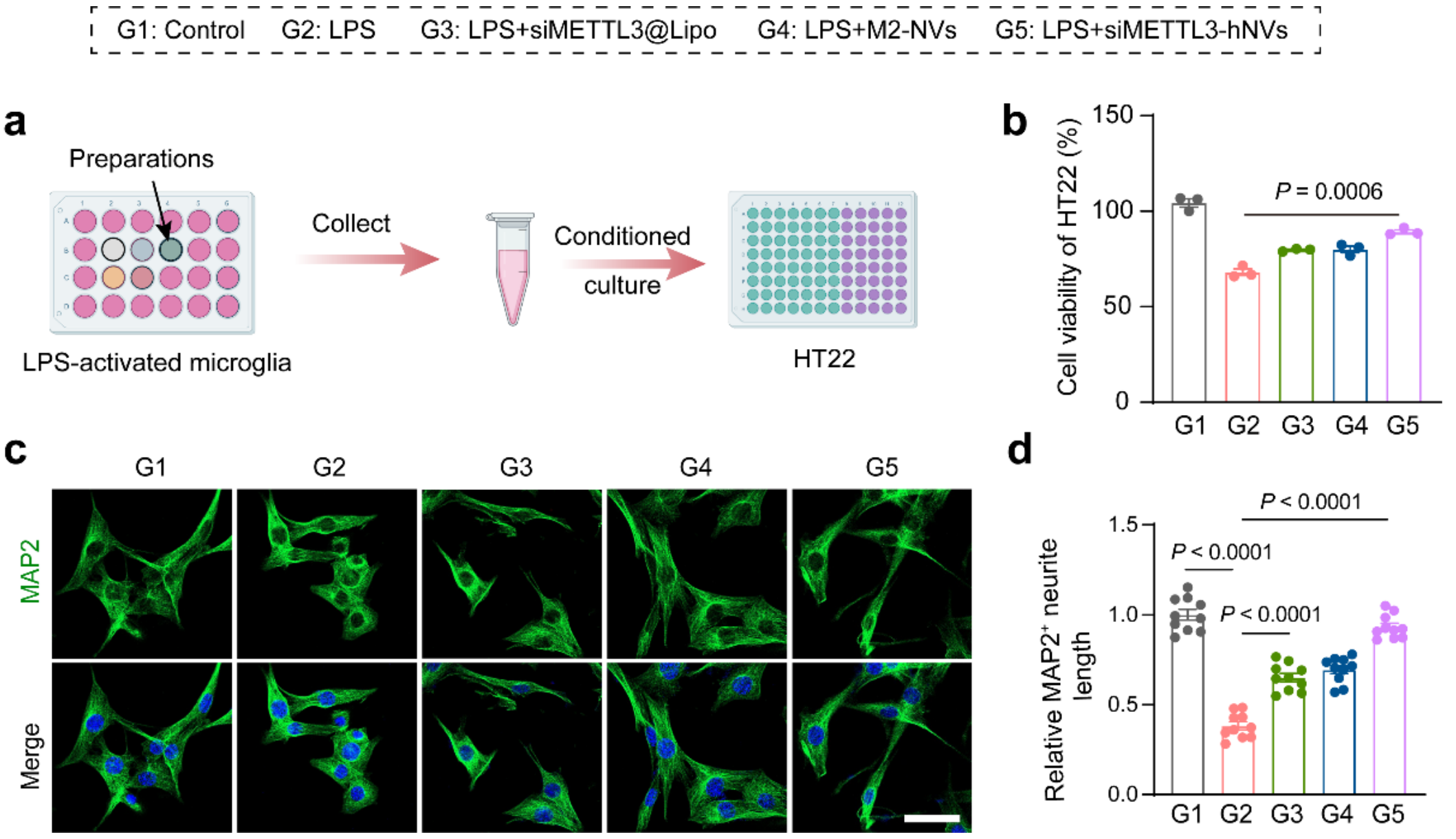

Supplementary Fig. 6 | Neuroprotection in microglia-neuron models. a, Schematic of the experimental design for cytokine analysis and HT22 neuron conditioned culture. Created in BioRender. Xu, L. (2026) https://BioRender.com/n69hlel. **b,** Viability of HT22 cells incubated with conditioned medium collected from differently treated BV2 cells (n = 3 independent experiments). **c, d** Immunofluorescence images (**c**) and quantification (**d**) of relative MAP2-positive neurite length in HT22 neurons after different treatments (n = 10 independent experiments; exact P values: G2 vs. G1 P = 7.66E−22; G2 vs. G3 P = 1.67E−9; G2 vs. G5 P = 6.87E−20). G1, Control; G2, LPS; G3, LPS + siMETTL3@Lipo; G4, LPS + M2-NVs; G5, LPS + siMETTL3-hNVs. Scale bar, 10 μm. For **b** and **d**, data are presented as mean ± s.e.m., with statistical significance assessed using one-way ANOVA followed by Tukey's multiple comparison test.

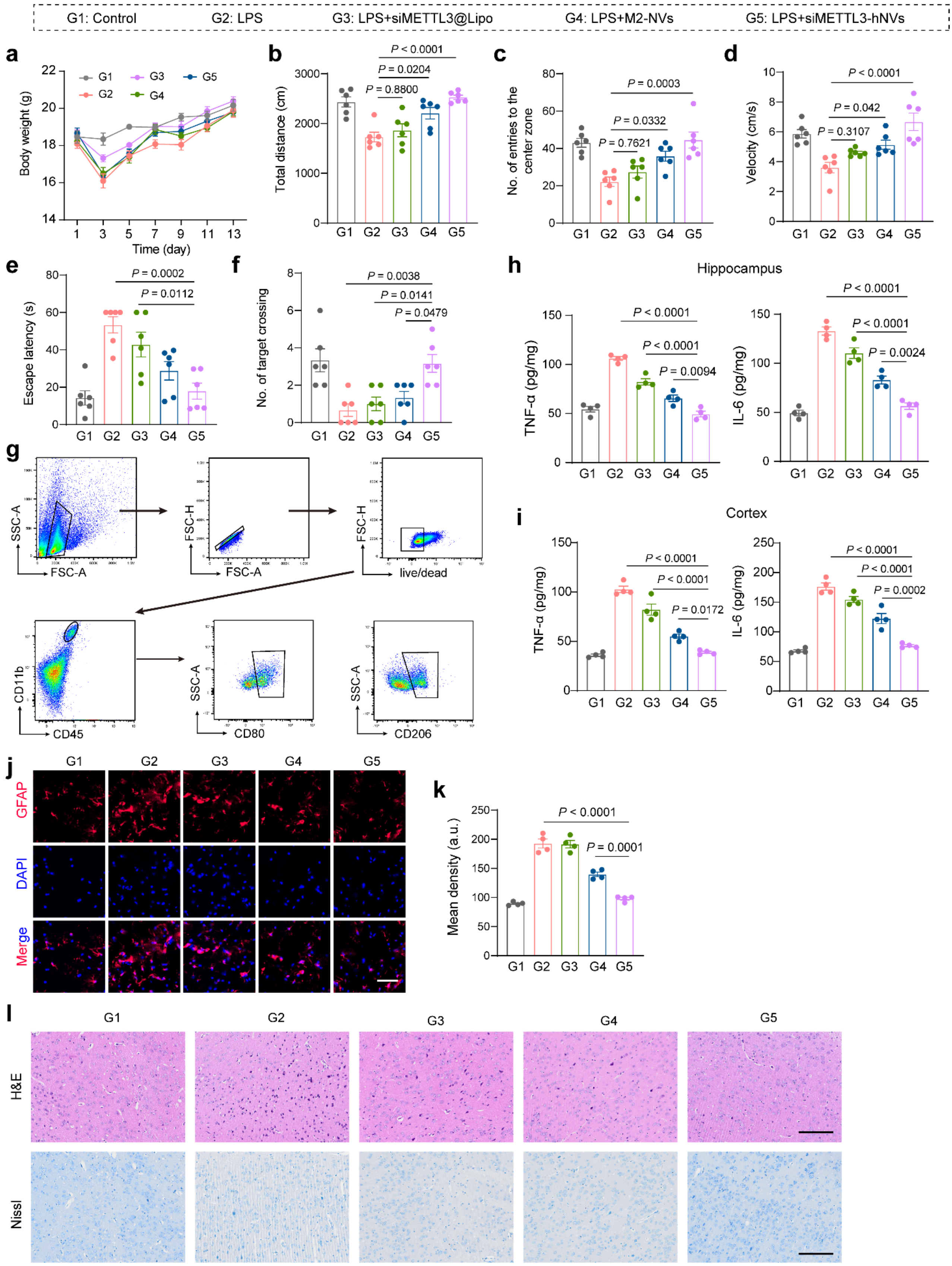

Supplementary Fig. 7 | In vivo therapeutic efficacy of siMETTL3-hNVs in lipopolysaccharide-induced neuroinflammation. a, Body weight curves of LPS-induced neuroinflammatory mice after different treatments (n = 6 biologically independent mice). **b–d,** Quantitative analysis of total distance

(**b**), number of entries into the centre zone (**c**) and velocity (**d**) in the open-field test (*n* = 6 biologically independent mice; exact *P* values: **b,** G2 vs. G5 *P* = 7.87E−5; **d,** G2 vs. G5 *P* = 2.51E−5). **e, f** Quantification of latency in the probe test (**e**) and number of target crossings (**f**) in the Morris water maze test (*n* = 6 biologically independent mice). **g,** Gating strategy for M1-like and M2-like microglia, showing the identification of $CD45^{int}CD11b^{+}CD80^{+}$ M1-like and $CD45^{int}CD11b^{+}CD206^{+}$ M2-like microglia. **h, i** ELISA analysis of TNF-α and IL-6 levels in the hippocampus (**h**) and cortex (**i**) after different treatments (*n* = 4 biologically independent mice; exact *P* values: **h,** TNF-α, G2 vs. G5 *P* = 4.64E−9, G3 vs. G5 *P* = 6.44E−6; **h,** IL-6, G2 vs. G5 *P* = 8.07E−9, G3 vs. G5 *P* = 8.97E−7; **i,** TNF-α, G2 vs. G5 *P* = 3.71E−9, G3 vs. G5 *P* = 7.12E−7; **i,** IL-6, G2 vs. G5 *P* = 1.04E−8, G3 vs. G5 *P* = 2.92E−7). **j, k** GFAP immunofluorescence images (**j**) and quantification (**k**) of hippocampal sections after treatment (*n* = 4 biologically independent mice; exact *P* values: G2 vs. G5 *P* = 6.95E−10; G5 vs. G4 *P* = 9.90E−5). Scale bar, 40 μm. **l,** Representative H&E and Nissl staining images of the hippocampus after different treatments. Scale bar, 100 μm. G1, Control; G2, LPS; G3, LPS + siMETTL3@Lipo; G4, LPS + M2-NVs; G5, LPS + siMETTL3-hNVs. Experiments were performed using six-week-old female C57BL/6J mice. For **a–f**, **h**, **i** and **k**, data are presented as mean ± s.e.m., with statistical significance assessed using one-way ANOVA followed by Tukey's multiple comparison test (**b–f**, **h**, **i** and **k**).

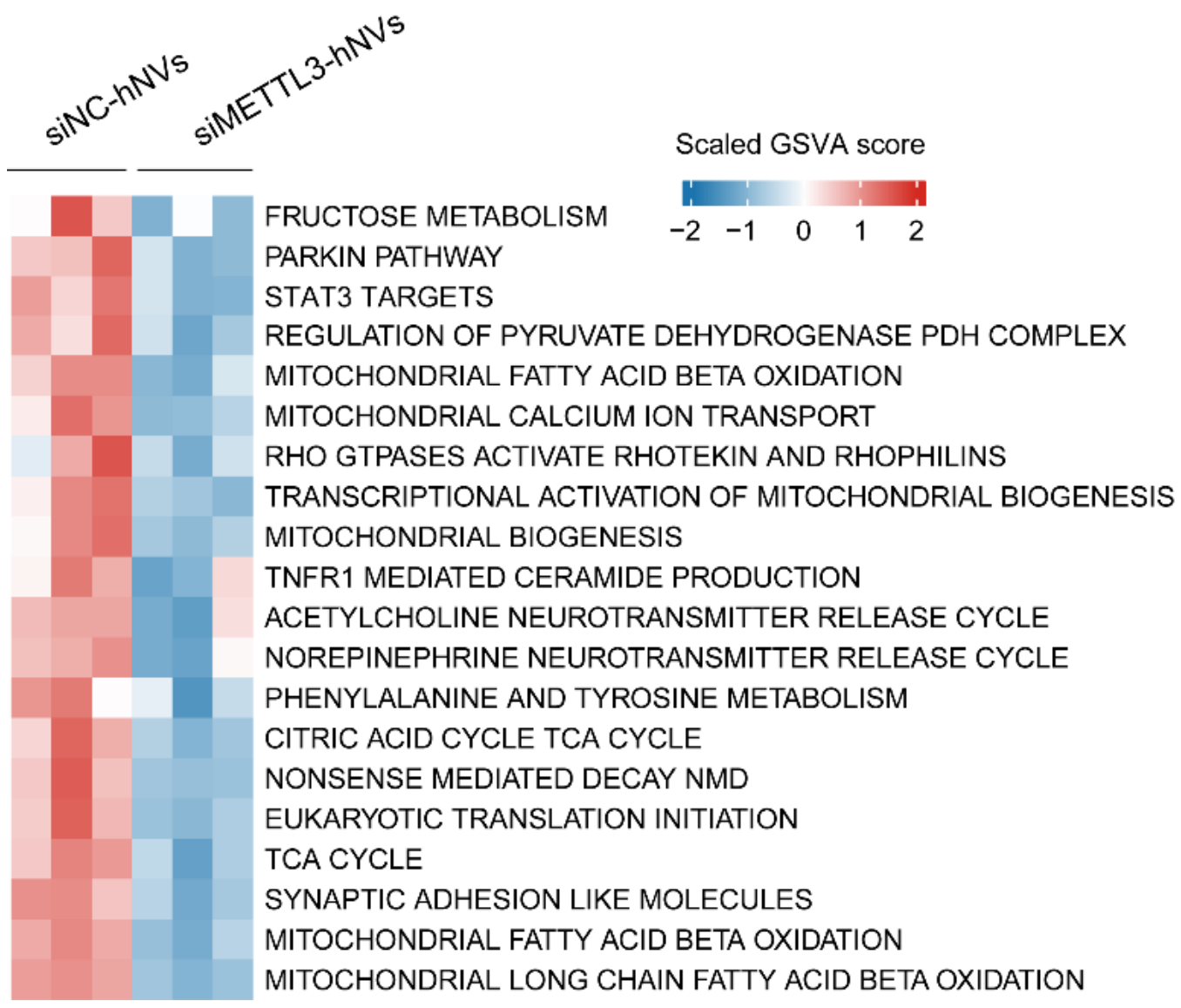

Supplementary Fig. 8 | Heatmap shows the selected signaling pathways (rows) that were significantly enriched in GSVA analyses.

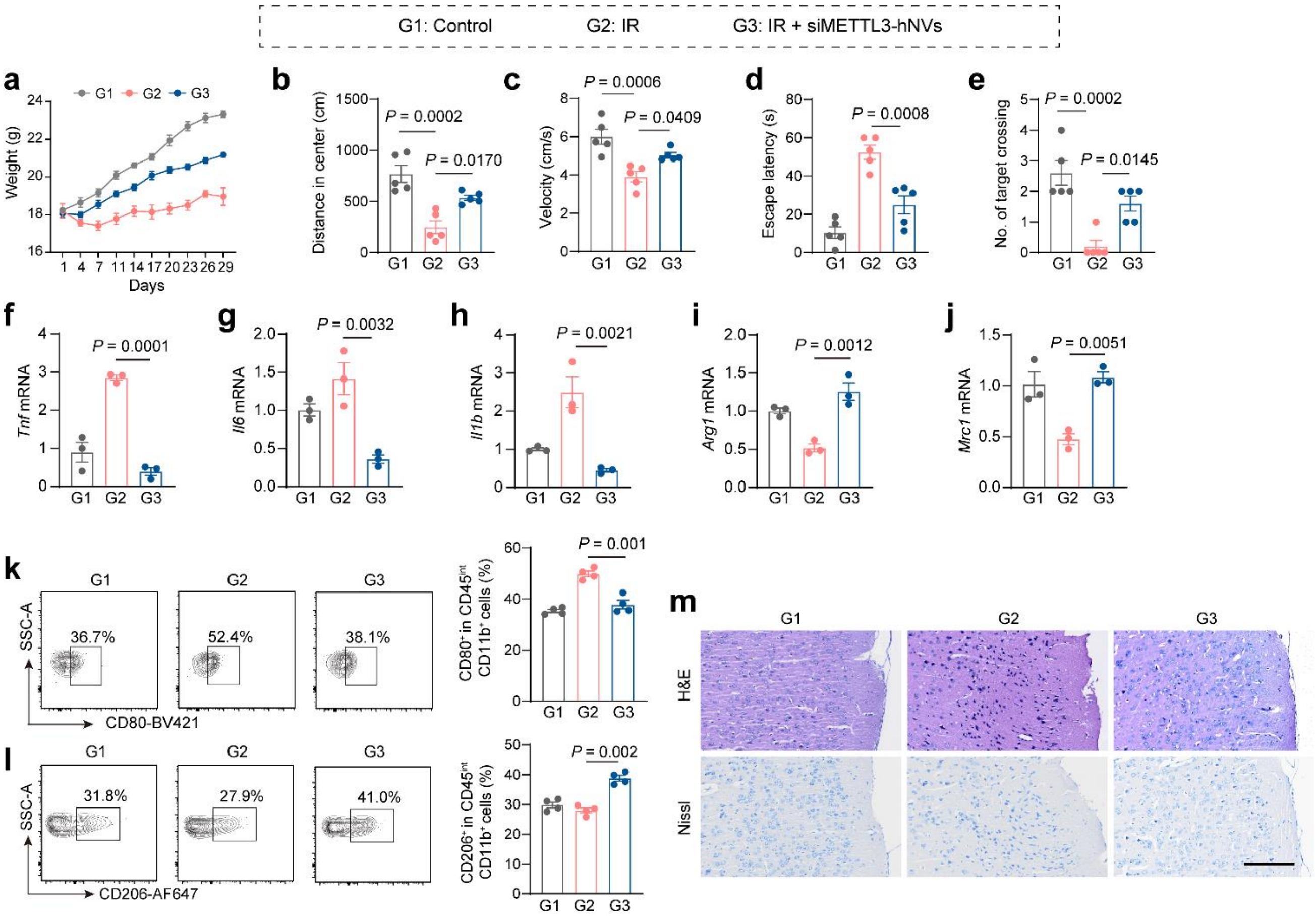

Supplementary Fig. 9 | Behavioral recovery and inflammatory microenvironment remodeling in radiation-induced brain injury. a, Body weight curves of RIBI mice after different treatments (*n* = 5 biologically independent mice). **b, c** Quantitative analysis of centre distance (**b**) and velocity (**c**) in the open-field test (*n* = 5 biologically independent mice). **d, e** Quantification of latency in the probe test (**d**) and number of target crossings (**e**) in the Morris water maze test (*n* = 5 biologically independent mice). **f–j,** qRT-PCR analysis of *Tnf*, *Il6*, *Il1b*, *Arg1* and *Mrc1* expression in brain tissues after different treatments (*n* = 3 biologically independent mice). **k, l** Flow cytometric quantification of $CD80^+$ (**k**) and $CD206^+$ (**l**) microglia in brains from RIBI mice (*n* = 4 biologically independent mice). **m,** Representative haematoxylin and eosin and Nissl staining images of cortical sections after different treatments (*n* = 3 biologically independent mice). Scale bars, 100 μm. Experiments were performed using six-week-old female C57BL/6J mice. For **a–l**, data are presented as mean ± s.e.m., with statistical significance assessed using one-way ANOVA followed by Tukey's multiple comparison test (**b-l**).

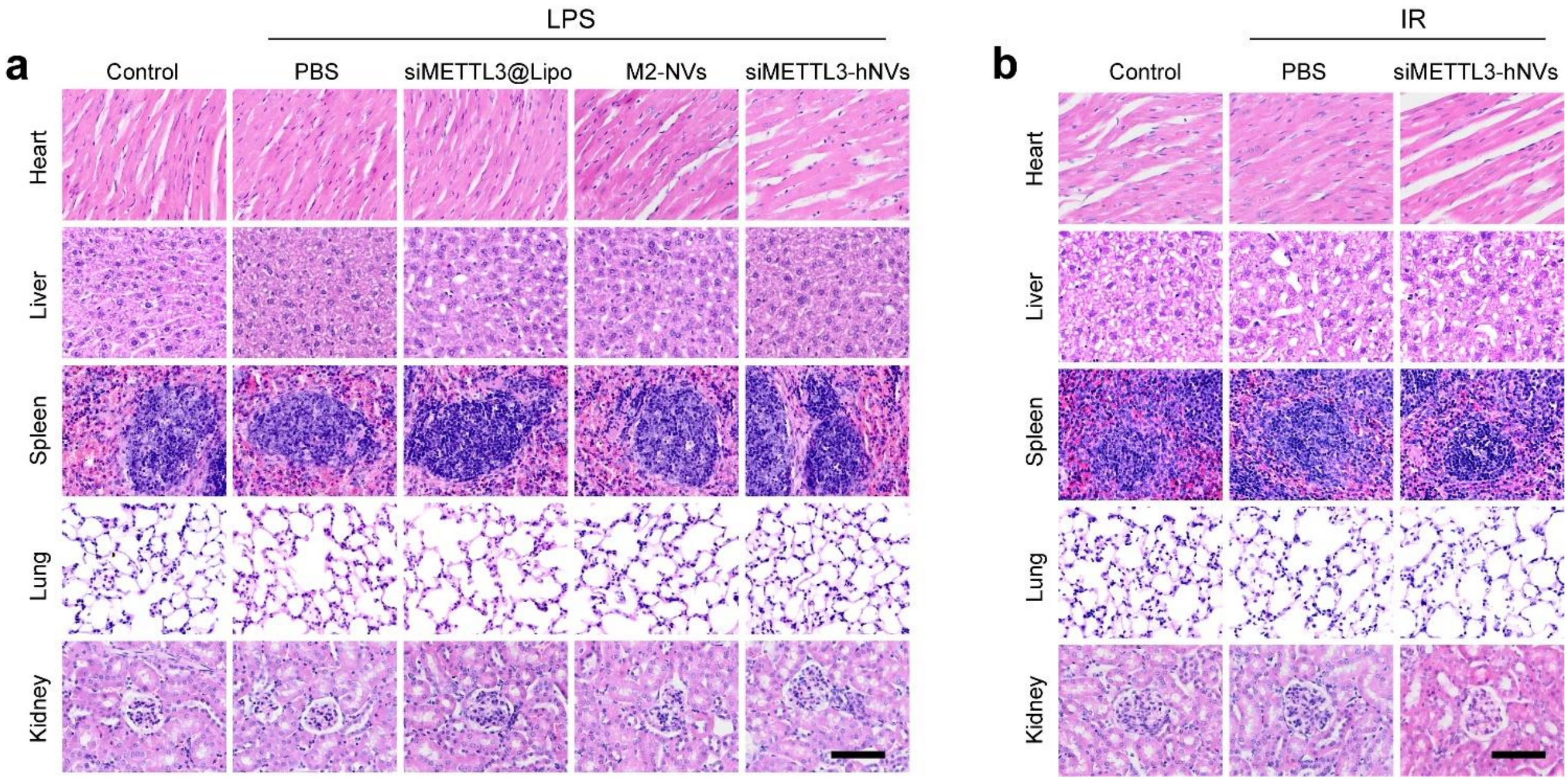

Supplementary Fig. 10 | *In vivo* biosafety evaluation. Histological analysis of heart, liver, spleen, lung, and kidney tissues from (**a**) LPS-induced neuroinflammation mice and (**b**) RIBI mice, stained with H&E (n = 3 biologically independent mice). Scale bar, 100 μm. Experiments were performed using six-week-old female C57BL/6J mice.

Uncropped and unprocessed scans

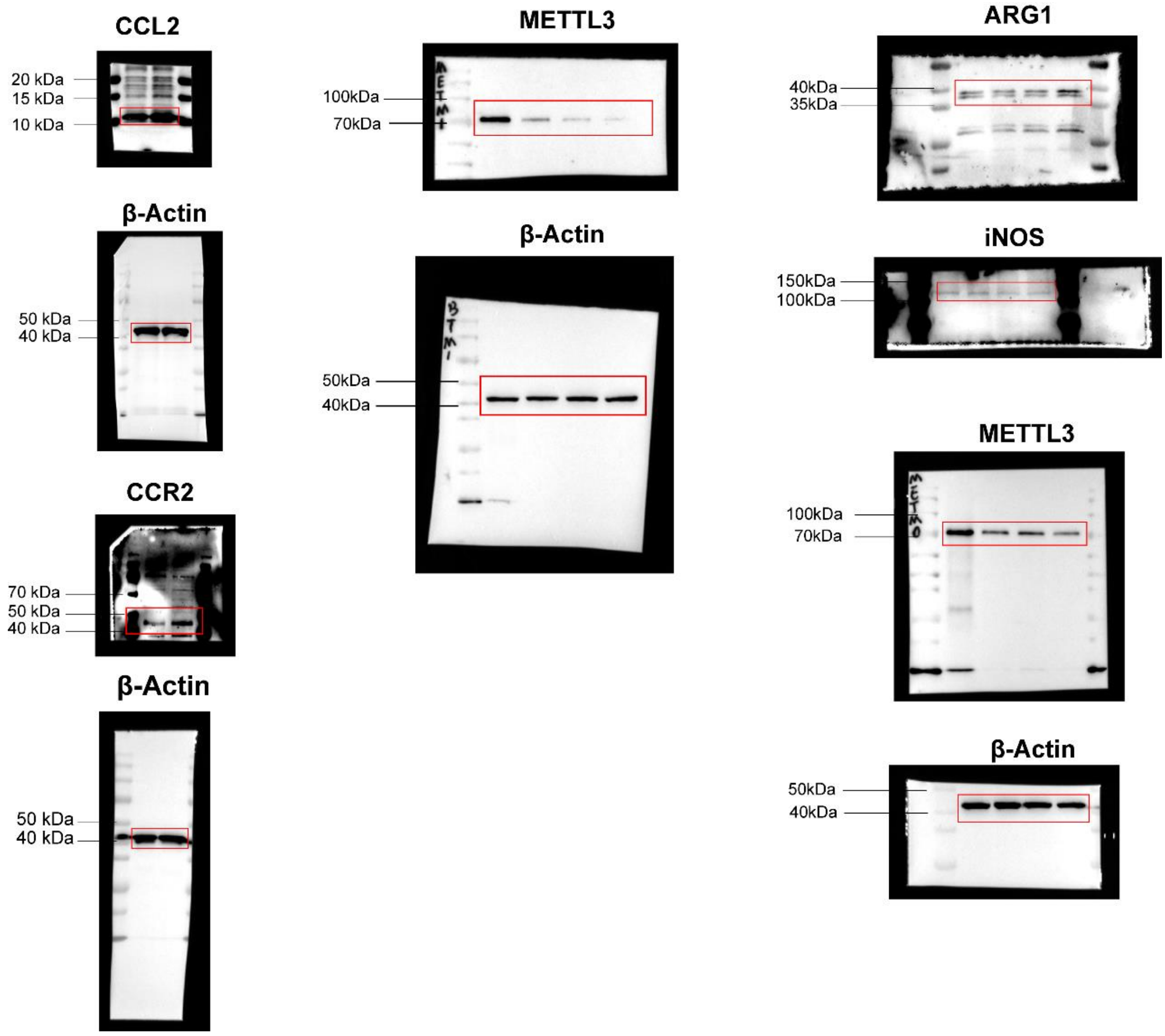

Supplementary table 1. List of M2 macrophage activation-associated genes.

The M2 macrophage activation-associated genes						
STAT6	SOCS1	STAT3	MMP12	CCL18	PPARG	SOCS3
NFIL3	KLF4	ALOX15	MMP1	CCL17	SOCS2	SBNO2
DUSP6	RIPK3	TGM2	CD163	CCL13	GATA3	SMAD2
IRF4	PTX3	IL17RB	CD206	CCL4	ID3	RGS1

Supplementary table 2. List of oligonucleotides

Name	Sequence	Note
18S rRNA-qPCR-F	TGTGCCGCTAGAGGTGAAATT	Primers for qPCR
18S rRNA-qPCR-R	TGGCAAATGCTTTCGCTTT	
METTL3-F	CGTAGTGATAGTCCCGTGCC	
METTL3-R	TGGCGTAGAGATGGCAAGAC	
ARG1-F	ACATTGGCTTGCGAGACGTA	
ARG1-R	ATCACCTTGCCAATCCCCAG	
MRC1-F	GGCTGATTACGAGCAGTGGA	
MRC1-R	ACATGCCAGGGTCACCTTTC	
NOS2-F	GAGCCACAGTCCTCTTTGCT	
NOS2-R	ACCACCAGCAGTAGTTGCTC	
TNF-α-F	CCTCACACTCACAAACCACCA	
TNF-α-R	ACAAGGTACAACCCATCGGC	
IL-1β-F	ATGCCACCTTTTGACAGTGATG	
IL-1β-R	TGTGCTGCTGCGAGATTTGA	
IL-6-F	TGATGGATGCTACCAAACTGGA	
IL-6-R	TCTGTGACTCCAGCTTATCTCTTG	
IL-4-F	GTACCAGGAGCCATATCCACG	
IL-4-R	GTTGCTGTGAGGACGTTTGG	
IL-10-F	TGAATTCCCTGGGTGAGAAGC	
IL-10-R	CATTCATGGCCTTGTAGACACC	
SOCS3-F	CTGCAGGAGAGCGGATTCTA	
SOCS3-R	CTCACACTGGATGCGTAGGT	
GAPDH-F	GGGTCCCAGCTTAGGTTCATC	
GAPDH-R	ATCCGTTCACACCGACCTTC	
METTL3 siRNA sense-1	CACCUUGGGAUAUUCACAUTT	Targeting METTL3 mRNA or negative control
METTL3 siRNA antisense-1	AUGUGAAUAUCCCAAGGUGTT	
METTL3 siRNA sense-2	CCUCCAAGAUGAUGCACAUTT	
METTL3 siRNA antisense-2	AUGUGCAUCAUCUUGGAGGTT	
METTL3 siRNA sense-3	CCUGCAAAUAUGUUCACUATT	
METTL3 siRNA antisense-3	UAGUGAACAUAUUUGCAGGTT	
NC siRNA sense	UUCUCCGAACGUGUCACGUTT	
NC siRNA antisense	ACGUGACACGUUCGGAGAATT	
