# Supplementary material for: Targeting m6A writer METTL3 with engineered nanovesicles reduces neuroinflammation in vitro and in vivo
Source: Nat Commun. 2026 Aug 8;17:9534. doi: 10.1038/s41467-026-75862-4 (PMC13547405; doi:10.1038/s41467-026-75862-4)
Supplement: Supplementary file 2 [file 41467_2026_75862_MOESM2_ESM.pdf]

nature portfolio

Corresponding author(s): Qianfang Meng, Zhigang Liu, Lang Rao

Last updated by author(s): Jun 13, 2026

Reporting Summary

Nature Portfolio wishes to improve the reproducibility of the work that we publish. This form provides structure for consistency and transparency in reporting. For further information on Nature Portfolio policies, see our Editorial Policies and the Editorial Policy Checklist.

Statistics

For all statistical analyses, confirm that the following items are present in the figure legend, table legend, main text, or Methods section.

n/a	Confirmed	
☐	☒	The exact sample size (*n*) for each experimental group/condition, given as a discrete number and unit of measurement
☐	☒	A statement on whether measurements were taken from distinct samples or whether the same sample was measured repeatedly
☐	☒	The statistical test(s) used AND whether they are one- or two-sided<br>*Only common tests should be described solely by name; describe more complex techniques in the Methods section.*
☒	☐	A description of all covariates tested
☐	☒	A description of any assumptions or corrections, such as tests of normality and adjustment for multiple comparisons
☐	☒	A full description of the statistical parameters including central tendency (e.g. means) or other basic estimates (e.g. regression coefficient) AND variation (e.g. standard deviation) or associated estimates of uncertainty (e.g. confidence intervals)
☐	☒	For null hypothesis testing, the test statistic (e.g. *F*, *t*, *r*) with confidence intervals, effect sizes, degrees of freedom and *P* value noted<br>*Give P values as exact values whenever suitable.*
☒	☐	For Bayesian analysis, information on the choice of priors and Markov chain Monte Carlo settings
☒	☐	For hierarchical and complex designs, identification of the appropriate level for tests and full reporting of outcomes
☒	☐	Estimates of effect sizes (e.g. Cohen's *d*, Pearson's *r*), indicating how they were calculated

Our web collection on statistics for biologists contains articles on many of the points above.

Software and code

Policy information about availability of computer code

Data collection	TEM images were collected using Hitachi HT7800. Confocal images were collected using ZEISS LSM980. DLS and zeta potential data were collected using Malvern Zetasizer Nano-ZS90. Flow cytometry data were collected using CytoFLEX S. qRT-PCR data were collected using QuantStudio 5 and Roche LightCycler 480 II. In vivo fluorescence images were collected using IVIS Spectrum and Living Image 4.8.4. Behavioural tracking data were collected using SMART v3.06. RNA-seq data were generated using Illumina NovaSeq 6000.
Data analysis	Data analysis was performed using GraphPad Prism 9.5, Fiji/ImageJ 1.54p, FlowJo v10.8.1, Living Image 4.8.4 and R 4.4.3. Sequencing data were analyzed using FastQC v0.11.9, Trimmomatic v0.39, HISAT2 v2.2.1, SAMtools v1.12, exomePeak2 v2.0.0, HOMER v4.11 and IGV v2.12.3. Differential expression was assessed using limma-voom with empirical Bayes moderated t-test and Benjamini–Hochberg adjustment. Plots were generated using ggplot2 in R.

For manuscripts utilizing custom algorithms or software that are central to the research but not yet described in published literature, software must be made available to editors and reviewers. We strongly encourage code deposition in a community repository (e.g. GitHub). See the Nature Portfolio guidelines for submitting code & software for further information.

Data

Policy information about availability of data

All manuscripts must include a data availability statement. This statement should provide the following information, where applicable:

- Accession codes, unique identifiers, or web links for publicly available datasets
- A description of any restrictions on data availability
- For clinical datasets or third party data, please ensure that the statement adheres to our policy

All data generated or analyzed during this study are available within the article, Supplementary Information or Source Data file. The RNA-seq data generated in this study have been deposited in the Sequence Read Archive under accession number PRJNA1400104. The public m6A-seq dataset analyzed in this study is available from the Gene Expression Omnibus under accession number GSE264486. Source data are provided with this paper.

Research involving human participants, their data, or biological material

Policy information about studies with human participants or human data. See also policy information about sex, gender (identity/presentation), and sexual orientation and race, ethnicity and racism.

Reporting on sex and gender	N/A
Reporting on race, ethnicity, or other socially relevant groupings	N/A
Population characteristics	N/A
Recruitment	N/A
Ethics oversight	N/A

Note that full information on the approval of the study protocol must also be provided in the manuscript.

Field-specific reporting

Please select the one below that is the best fit for your research. If you are not sure, read the appropriate sections before making your selection.

[x] Life sciences [ ] Behavioural & social sciences [ ] Ecological, evolutionary & environmental sciences

For a reference copy of the document with all sections, see nature.com/documents/nr-reporting-summary-flat.pdf

Life sciences study design

All studies must disclose on these points even when the disclosure is negative.

Sample size	Sample sizes were established based on prior pilot studies, experimental experience, and standard protocols within the field (Feng, Yunxuan et al., 2025, Nature Materials). For instance, at least three mice were evaluated to assess statistical similarities or differences across groups.
Data exclusions	No data were excluded from the study.
Replication	All the biological experiments were repeated at least three times and the experimental findings were highly reproducible.
Randomization	To minimize potential bias, samples and animals were randomly allocated during the grouping process in vitro and in vivo.
Blinding	All the investigators were blinded to group allocation for data collection and analysis.

Reporting for specific materials, systems and methods

We require information from authors about some types of materials, experimental systems and methods used in many studies. Here, indicate whether each material, system or method listed is relevant to your study. If you are not sure if a list item applies to your research, read the appropriate section before selecting a response.

Materials & experimental systems

n/a	Involved in the study
☐	☒ Antibodies
☐	☒ Eukaryotic cell lines
☒	☐ Palaeontology and archaeology
☐	☒ Animals and other organisms
☒	☐ Clinical data
☒	☐ Dual use research of concern
☒	☐ Plants

Methods

n/a	Involved in the study
☒	☐ ChIP-seq
☐	☒ Flow cytometry
☒	☐ MRI-based neuroimaging

Antibodies

Antibodies used

1. Fixable Viability Stain 510 (BD Biosciences; Cat# 564406; 1:1000).
2. PerCP-Cy5.5 anti-mouse CD45 (BD Biosciences; Clone 30-F11; Cat# 550994; 1:200).
3. FITC anti-mouse CD11b (Invitrogen; Clone M1/70; Cat# 11-0112-82; 1:200).
4. Brilliant Violet 421 anti-mouse CD80 (BD Biosciences; Clone 16-10A1; Cat# 562611; 1:200).
5. Brilliant Violet 421 anti-mouse CD86 (BD Biosciences; Clone GL1; Cat# 564198; 1:200).
6. Alexa Fluor 647 anti-mouse CD206 (MMR) (BioLegend; Clone C068C2; Cat# 141712; 1:200).
7. IBA1 (ABclonal; Cat# A19776; source: rabbit; 1:200 for IF).
8. GFAP (Cell Signaling Technology; Cat# 80788; source: rabbit; 1:200 for IF).
9. CD80 (ABclonal; Cat# A16039; source: rabbit; 1:200 for IF).
10. CD206 (ABclonal; Cat# A21014; source: rabbit; 1:1000 for WB; 1:200 for IF).
11. IL1R1 (Proteintech; Cat# 27348-1-AP; source: rabbit; 1:1000 for WB).
12. IL6R (Proteintech; Cat# 23457-1-AP; source: rabbit; 1:1000 for WB).
13. TNFR1 (Proteintech; Cat# 21574-1-AP; source: rabbit; 1:1000 for WB).
14. Na,K-ATPase (Cell Signaling Technology; Cat# 3010; source: rabbit; 1:1000 for WB).
15. iNOS (Proteintech; Cat# 22226-1-AP; source: rabbit; 1:1000 for WB).
16. Arg1 (Proteintech; Cat# 16001-1-AP; source: rabbit; 1:1000 for WB).
17. CCL2 (Proteintech; Cat# 26161-1-AP; source: rabbit; 1:1000 for WB).
18. CCR2 (Proteintech; Cat# 16153-1-AP; source: rabbit; 1:1000 for WB).
19. β-actin (Cell Signaling Technology; Cat# 4970; source: rabbit; 1:1000 for WB).
20. METTL3 (ABclonal; Cat# A19079; source: rabbit; 1:1000 for WB).
21. BAX (Proteintech; Cat# 50599-2-Ig; source: rabbit; 1:1000 for WB).
22. Cleaved caspase-3 (Proteintech; Cat# 87055-4-RR; source: rabbit; 1:1000 for WB).
23. BCL-2 (Proteintech; Cat# 26593-1-AP; source: rabbit; 1:1000 for WB).
24. Phospho-JAK2 (ABclonal; Cat# AP0531; source: rabbit; 1:1000 for WB).
25. Phospho-STAT3 (ABclonal; Cat# AP0705; source: rabbit; 1:1000 for WB).
26. SOCS3 (ABclonal; Cat# A0769; source: rabbit; 1:1000 for WB).
27. Goat Anti-Rabbit IgG H&L (FITC) (Abcam; Cat# ab6717; 1:500 for IF).
28. Goat Anti-Rabbit IgG H&L (Alexa Fluor® 647) (Abcam; Cat# ab150079; 1:500 for IF).
29. PE anti-mouse CD31 (BD Biosciences; Clone MEC13.3; Cat# 553373; 1:200)
30. ACSA-2 Antibody, anti-mouse (Miltenyi Biotec; Clone REA969; Cat# 130-116-248)
31. NeuN Antibody, anti-mouse (Miltenyi Biotec; Clone REA1131; Cat# 130-136-607)

Validation

All antibodies used in our study are commercially available and have been previously documented and routinely utilized for their respective applications. The suppliers implement quality control protocols to guarantee their validity and reproducibility. Detailed validation information and prior references for each antibody can be found in the data sheets supplied by the manufacturers.
1. Fixable Viability Stain 510 has been validated to be used for flow cytometry analysis for the discrimination of viable from non-viable mammalian cells. (Data is provided on their website: https://www.bdbiosciences.com/zh-cn/products/reagents/flow-cytometry-reagents/research-reagents/single-color-antibodies-ruo/fixable-viability-stain-510.564406).
2. PerCP-Cy5.5 anti-mouse CD45 antibody has been validated to be used for flow cytometry analysis and mentioned species reactivity with mouse. (Data is provided on their website: https://www.bdbiosciences.com/zh-cn/products/reagents/flow-cytometry-reagents/research-reagents/single-color-antibodies-ruo/percp-cy-5-5-rat-anti-mouse-cd45.550994).
3. FITC anti-mouse CD11b antibody has been validated to be used for flow cytometry analysis and mentioned species reactivity with mouse. (Data is provided on their website: https://www.thermofisher.cn/cn/zh/antibody/product/CD11b-Antibody-clone-M1-70-Monoclonal/11-0112-82).
4. Brilliant Violet 421 anti-mouse CD80 antibody has been validated to be used for flow cytometry analysis and mentioned species reactivity with mouse. (Data is provided on their website: https://www.bdbiosciences.com/zh-cn/products/reagents/flow-cytometry-reagents/research-reagents/single-color-antibodies-ruo/bv421-hamster-anti-mouse-cd80.562611).
5. Brilliant Violet 421 anti-mouse CD86 antibody has been validated to be used for flow cytometry analysis and mentioned species reactivity with mouse. (Data is provided on their website: https://www.bdbiosciences.com/zh-cn/products/reagents/flow-cytometry-reagents/research-reagents/single-color-antibodies-ruo/bv421-rat-anti-mouse-cd86.564198).
6. Alexa Fluor 647 anti-mouse CD206 (MMR) antibody has been validated to be used for flow cytometry analysis and mentioned species reactivity with mouse. (Data is provided on their website: https://www.biolegend.com/en-gb/products/alexa-fluor-647-anti-

mouse-cd206-mmr-antibody-7427).
7. IBA1 antibody (ABclonal, A19776) has been validated to be used for Immunofluorescence analysis and mentioned species reactivity with mouse. (Data is provided on their website: https://abclonal.com.cn/catalog/A19776).
8. GFAP antibody (CST, 80788) has been validated to be used for Immunofluorescence analysis and mentioned species reactivity with mouse. (Data is provided on their website: https://www.cellsignal.cn/products/primary-antibodies/gfap-e4l7m-rabbit-monoclonal-antibody/80788).
9. CD80 antibody (ABclonal, A16039) has been validated to be used for Immunofluorescence analysis and mentioned species reactivity with mouse. (Data is provided on their website: https://abclonal.com.cn/catalog/A16039).
10. CD206 antibody (ABclonal, A21014) has been validated to be used for Western blotting and Immunofluorescence analysis and mentioned species reactivity with mouse. (Data is provided on their website: https://abclonal.com.cn/catalog/A21014).
11. IL1R1 antibody (Proteintech, 27348-1-AP) has been validated to be used for Western blotting analysis and mentioned species reactivity with mouse. (Data is provided on their website: https://www.ptgcn.com/products/IL-1R1-Antibody-27348-1-AP.htm).
12. IL6R antibody (Proteintech, 23457-1-AP) has been validated to be used for Western blotting analysis and mentioned species reactivity with mouse. (Data is provided on their website: https://www.ptgcn.com/products/IL6R-Antibody-23457-1-AP.htm).
13. TNFR1 antibody (Proteintech, 21574-1-AP) has been validated to be used for Western blotting analysis and mentioned species reactivity with mouse. (Data is provided on their website: https://www.ptgcn.com/products/TNFR1-Antibody-21574-1-AP.htm).
14. Na,K-ATPase antibody (CST, 3010) has been validated to be used for Western blotting analysis and mentioned species reactivity with mouse. (Data is provided on their website: https://www.cellsignal.cn/products/primary-antibodies/na-k-atpase-antibody/3010).
15. iNOS antibody (Proteintech, 22226-1-AP) has been validated to be used for Western blotting analysis and mentioned species reactivity with mouse. (Data is provided on their website: https://www.ptgcn.com/products/NOS2-Antibody-22226-1-AP.htm).
16. Arginase-1 (Arg1) antibody (Proteintech, 16001-1-AP) has been validated to be used for Western blotting analysis and mentioned species reactivity with mouse. (Data is provided on their website: https://www.ptgcn.com/products/ARG1-Antibody-16001-1-AP.htm).
17. MCP1 (CCL2) antibody (Proteintech, 26161-1-AP) has been validated to be used for Western blotting analysis and mentioned species reactivity with mouse. (Data is provided on their website: https://www.ptgcn.com/products/Mcp1-Antibody-26161-1-AP.htm).
18. CCR2 antibody (Proteintech, 16153-1-AP) has been validated to be used for Western blotting analysis and mentioned species reactivity with mouse. (Data is provided on their website: https://www.ptgcn.com/products/CCR2a-specific-Antibody-16153-1-AP.htm).
19. β-Actin antibody (CST, 4970T) has been validated to be used for Western blotting analysis and mentioned species reactivity with mouse. (Data is provided on their website: https://www.cellsignal.cn/products/primary-antibodies/beta-actin-13e5-rabbit-monoclonal-antibody/4970).
20. METTL3 antibody (ABclonal, A19079) has been validated to be used for Western blotting analysis and mentioned species reactivity with mouse. (Data is provided on their website: https://abclonal.com.cn/catalog/A19079).
21. BAX antibody (Proteintech, 50599-2-Ig) has been validated to be used for Western blotting analysis and mentioned species reactivity with mouse. (Data is provided on their website: https://www.ptgcn.com/products/BAX-Antibody-50599-2-Ig.htm).
22. Cleaved Caspase 3 antibody (Proteintech, 87055-4-RR) has been validated to be used for Western blotting analysis and mentioned species reactivity with mouse. (Data is provided on their website: https://www.ptgcn.com/products/Cleaved-Caspase-3-P12-Antibody-87055-4-RR.htm).
23. BCL2 antibody (Proteintech, 26593-1-AP) has been validated to be used for Western blotting analysis and mentioned species reactivity with mouse. (Data is provided on their website: https://www.ptgcn.com/products/Bcl2-Antibody-26593-1-AP.htm).
24. Phospho-JAK2 antibody (ABclonal, AP0531) has been validated to be used for Western blotting analysis and mentioned species reactivity with mouse. (Data is provided on their website: https://abclonal.com.cn/catalog/AP0531).
25. Phospho-STAT3 antibody (ABclonal, AP0705) has been validated to be used for Western blotting analysis and mentioned species reactivity with mouse. (Data is provided on their website: https://abclonal.com.cn/catalog/AP0705).
26. SOCS3 antibody (ABclonal, A0769) has been validated to be used for Western blotting analysis and mentioned species reactivity with mouse. (Data is provided on their website: https://abclonal.com.cn/catalog/A0769).
27. Goat Anti-Rabbit IgG H&L (FITC) (Abcam, ab6717) has been validated to be used for Immunofluorescence analysis and mentioned reactivity with rabbit IgG. (Data is provided on their website: https://www.abcam.cn/products/secondary-antibodies/goat-rabbit-igg-h-l-fitc-ab6717).
28. Goat Anti-Rabbit IgG H&L (Alexa Fluor® 647) (Abcam, ab150079) has been validated to be used for Immunofluorescence analysis and mentioned reactivity with rabbit IgG. (Data is provided on their website: https://www.abcam.cn/products/secondary-antibodies/goat-rabbit-igg-h-l-alexa-fluor-647-ab150079).
29. PE anti-mouse CD31 has been validated to be used for flow cytometry analysis and mentioned species reactivity with mouse. (Data is provided on their website: BD Pharmingen™ PE Rat Anti-Mouse CD31)
30. ACSA-2 Antibody, anti-mouse, Vio® Bright B515, REAfinity™ has been validated to be used for flow cytometry analysis and mentioned species reactivity with mouse. (Data is provided on their website: ACSA-2 Antibody, anti-mouse, REAfinity™)
31. NeuN Antibody, anti-human/mouse/rat, REAfinity™ has been validated to be used for neuronal detection and is listed by the manufacturer for mouse-reactive applications, including flow-cytometry-compatible conjugate options. (Data is provided on their website: NeuN Antibody, anti-human/mouse/rat, REAfinity™)

Eukaryotic cell lines

Policy information about cell lines and Sex and Gender in Research

Cell line source(s)	All cell lines were obtained from the American Type Culture Collection (ATCC). All cell lines were authenticated using short tandem repeat profiling and routinely tested for mycoplasma contamination.
Authentication	All cell lines were authenticated by DNA short tandem repeat profiling.
Mycoplasma contamination	All cell lines underwent mycoplasma contamination testing and no evidence of contamination was detected.

Commonly misidentified lines (See ICLAC register)	The study did not include any cell lines known to be commonly misidentified.

Animals and other research organisms

Policy information about studies involving animals; ARRIVE guidelines recommended for reporting animal research, and Sex and Gender in Research

Laboratory animals	Healthy C57BL/6J mice (female, 6-8 weeks old) were procured from GemPharmatech Co., Ltd (Nanjing, China). All animal experiments were conducted in accordance with the principles of animal experiment protection guidelines. The animals were maintained in a specific pathogen-free grade facility, with air humidity ranging from 40% to 70%, an ambient temperature of 22 ± 1 ° C, and a 12-hour dark/light cycle.
Wild animals	The study did not involve wild animals.
Reporting on sex	All animals involved in the experiments were female.
Field-collected samples	The study did not involve samples collected from field.
Ethics oversight	All animal experiments were reviewed and approved by the Institutional Animal Care and Use Committee of Shenzhen Bay Laboratory (AERL202403) .

Note that full information on the approval of the study protocol must also be provided in the manuscript.

Plants

Seed stocks	N/A
Novel plant genotypes	N/A
Authentication	N/A

Flow Cytometry

Plots

Confirm that:

- [x] The axis labels state the marker and fluorochrome used (e.g. CD4-FITC).
- [ ] The axis scales are clearly visible. Include numbers along axes only for bottom left plot of group (a 'group' is an analysis of identical markers).
- [x] All plots are contour plots with outliers or pseudocolor plots.
- [x] A numerical value for number of cells or percentage (with statistics) is provided.

Methodology

Sample preparation	*Describe the sample preparation, detailing the biological source of the cells and any tissue processing steps used.*
Instrument	*Identify the instrument used for data collection, specifying make and model number.*
Software	*Describe the software used to collect and analyze the flow cytometry data. For custom code that has been deposited into a community repository, provide accession details.*
Cell population abundance	*Describe the abundance of the relevant cell populations within post-sort fractions, providing details on the purity of the samples and how it was determined.*
Gating strategy	*Describe the gating strategy used for all relevant experiments, specifying the preliminary FSC/SSC gates of the starting cell population, indicating where boundaries between "positive" and "negative" staining cell populations are defined.*

- [x] Tick this box to confirm that a figure exemplifying the gating strategy is provided in the Supplementary Information.
