# Supplementary material for: Targeting m6A writer METTL3 with engineered nanovesicles reduces neuroinflammation in vitro and in vivo
Source: Nat Commun. 2026 Aug 8;17:9534. doi: 10.1038/s41467-026-75862-4 (PMC13547405; doi:10.1038/s41467-026-75862-4)
Supplement: Supplementary file 3 [file 41467_2026_75862_MOESM3_ESM.pdf]

nature portfolio

Peer Review File

Targeting m6A writer METTL3 with engineered nanovesicles reduces neuroinflammation in vitro and in vivo

Corresponding Author: Professor Lang Rao

This file contains all reviewer reports in order by version, followed by all author rebuttals in order by version.

Version 0:

Reviewer comments:

Reviewer #1

(Remarks to the Author)
This is a well-executed and thoughtfully designed study that addresses an important challenge in CNS drug delivery for neuroinflammatory diseases. The hybrid nanovesicle strategy combining microglia-derived membranes with siRNA-loaded liposomes is conceptually strong, and the authors provide extensive molecular, cellular, and in vivo data supporting therapeutic efficacy. The work is timely and of broad interest to the biomaterials and neuroimmunology communities. The comments below are intended to clarify several mechanistic interpretations and refine the strength of certain claims, which I believe will further strengthen the manuscript.

1. The authors repeatedly state “specific targeting of microglia.” However, the data do not convincingly demonstrate that uptake by other brain-resident or infiltrating cell types (e.g., astrocytes, endothelial cells, neurons, peripheral immune cells) is minimal. At present, the results support preferential uptake by microglia rather than specificity. The authors should either temper the language or provide additional quantitative data demonstrating limited targeting of other cell populations.

2. The proposed CCR2-CCL2-mediated mechanism for BBB transmigration is conceptually unclear. While CCL2-CCR2 signaling is well established in immune cell chemotaxis during neuroinflammation, BBB crossing following systemic administration is primarily governed by endothelial transport mechanisms or barrier disruption. CCR2 is a microglial/immune cell receptor rather than a canonical BBB endothelial transcytosis receptor, making it difficult to reconcile how CCR2 inherited from microglial membranes directly mediates endothelial traversal. The presented data appear more consistent with enhanced post-BBB accumulation or inflammatory-site retention rather than CCR2-dependent BBB penetration per se. Additional experiments disentangling endothelial crossing from parenchymal homing (e.g., CCR2 blockade on vesicles or endothelial interaction assays) would be required to substantiate this mechanism.

3. If the CCR2–CCL2 axis is a key driver of brain targeting and BBB penetration, an important prediction is that vesicle accumulation in the brain should be low under healthy conditions where CCL2 expression is minimal. Quantitative biodistribution data in healthy (non-inflamed) mice would therefore be informative. In addition, examining how vesicle accumulation scales with the degree of neuroinflammation (e.g., across different LPS doses or time points) would help establish whether targeting efficiency correlates with CCL2 levels, as implied by the proposed chemokine-gradient mechanism.

Reviewer #2

(Remarks to the Author)
In this manuscript, Xu et al. constructed a hybrid epigenetic nanomodulator, siMETTL3-hNVs, by integrating natural M2 microglial-derived nanovesicles (NVs) with synthetic liposomes to deliver siRNA targeting the m6A writer METTL3 for neuroinflammation therapy. By leveraging the native CCR2-CCL2 axis inherited from the parent microglial membranes, the system can efficiently cross the BBB and preferentially accumulate in inflamed brain regions. The authors demonstrate a dual-modality therapeutic mechanism: the NVs act as molecular decoys to neutralize pro-inflammatory cytokines while the siRNA intracellularly silences METTL3 to prevent the m6A-mediated degradation of SOCS3 mRNA. This stabilization of SOCS3 potently inhibits the JAK2-STAT3 signaling pathway, effectively driving microglial repolarization from a pro-inflammatory M1 state to a restorative M2 phenotype. The efficacy of this platform was validated in mouse models of LPS-

induced neuroinflammation and RIBI. I have the following concerns:

1. In the Introduction, the authors should provide a more comprehensive background on the specific role of SOCS3 within the inflammatory signaling cascade and explain its pivotal importance in guiding the design of the epigenetic nanomodulator therapy. Currently, the manuscript briefly mentions "intracellularly restoring SOCS3 stability via METTL3 knockdown", but it does not fully articulate why SOCS3 is a primary target for resolving neuroinflammation. The authors should also provide the full names of these genes and proteins before using their respective abbreviations.

2. In Page 8, lines 181–184, the authors indicate that "activated microglia as a principal source of this chemokine during neuroinflammation". However, this conclusion is not sufficiently supported as the experiments only tested BV2 microglial cells following LPS stimulation. Since the study does not include comparative expression data from other brain cells, such as astrocytes or neurons, the claim that microglia are the principal source is not solid. The authors should evaluate the chemokine production levels in other relevant brain cell lines to determine the relative contribution of microglia compared to other cell types.

3. In Figure 1 and Figure 3o, the mechanistic illustration suggests that the nanomodulators utilize the CCR2-CCL2 axis by binding circulating CCL2 to facilitate blood-brain barrier (BBB) transport, followed by CCL2 detachment. However, several points require further clarification:
3.1. There is no experimental evidence provided to support the claim that CCL2 detaches from the nanoparticles after crossing the BBB.
3.2. The specific cellular mechanism by which the nanoparticles use the CCR2-CCL2 axis to traverse endothelial layers remains undefined.
3.3. The authors should validate this proposed crossing mechanism in vivo using inhibitory assays, such as targeting CCL2 production or its receptors on endothelial layers, or by using comparative models involving healthy versus inflamed mice.

Reviewer #3

(Remarks to the Author)
This manuscript presents a hybrid epigenetic nanomodulator (siMETTL3-hNVs) composed of microglia-derived nanovesicles (NVs) integrated with siMETTL3-loaded synthetic liposomes for neuroinflammation therapy. The concept of m^6A-based epigenetic intervention combined with cytokine decoying is innovative, and the therapeutic effects in neuroinflammation and radiation-induced brain injury models are potentially impactful. However, several key claims regarding CCR2-mediated active targeting, BBB penetration, and formulation properties/stability are not fully supported by the current evidence. Addressing the following concerns is necessary to strengthen rigor and interpretation.
1. CCR2-mediated active targeting is not convincingly demonstrated. The manuscript attributes improved brain delivery/targeting to CCR2-mediated chemokine receptor transport, but direct evidence appears insufficient.
2. BBB crossing is not fully proven; LPS model likely has compromised BBB integrity. A central claim is that siMETTL3-hNVs "efficiently penetrate the BBB," yet the LPS-induced acute neuroinflammation model is known to disrupt BBB integrity. Therefore, the observed brain accumulation may reflect passive leakage rather than active BBB transcytosis.
3. In Fig. 1c, the nanocarriers show zeta potential close to 0 mV, which often implies limited electrostatic stabilization and potential aggregation during storage. Long-term stability data under storage conditions should be added.
4. The trend in Fig. 1f suggests that increasing the fraction of M2-NVs leads to decreased siRNA encapsulation/stability. This is counterintuitive and needs mechanistic explanation.
5. Fig. K indicates a pH-responsive release property. However, the formulation description (NVs + liposomes) does not obviously imply pH-triggered release. Please clarify what component(s) confer pH sensitivity and provide compositional details.
6. In Fig. 3i–k, siMETTL3-hNVs exhibit higher delivery efficiency than M2-NVs. Since hNVs already incorporate NV components, the manuscript should explain why the hybrid outperforms native M2-NVs (especially if the latter is expected to have strong biological homing).
7. If "decoy cytokine neutralization" is a major mechanism, consider quantifying binding/neutralization capacity and its contribution relative to METTL3 knockdown.

Version 1:

Reviewer comments:

Reviewer #1

(Remarks to the Author)
The authors have satisfactorily addressed all of the reviewer comments. The revised manuscript is significantly improved, with additional mechanistic clarification, expanded experimental validation, and improved presentation of the data. The conclusions are now better supported by the results, and the overall translational significance of the study is clearer. I believe the manuscript is suitable for publication in its current form.

Reviewer #2

(Remarks to the Author)
The authors have fully addressed my concerns and have done a good job revising the manuscript. The current version is

now suitable for publication in Nature Communications.

Reviewer #3

(Remarks to the Author)
The authors have addressed my concerns.

Open Access This Peer Review File is licensed under a Creative Commons Attribution 4.0 International License, which permits use, sharing, adaptation, distribution and reproduction in any medium or format, as long as you give appropriate credit to the original author(s) and the source, provide a link to the Creative Commons license, and indicate if changes were made.
In cases where reviewers are anonymous, credit should be given to 'Anonymous Referee' and the source.
The images or other third party material in this Peer Review File are included in the article's Creative Commons license, unless indicated otherwise in a credit line to the material. If material is not included in the article's Creative Commons license and your intended use is not permitted by statutory regulation or exceeds the permitted use, you will need to obtain permission directly from the copyright holder.
To view a copy of this license, visit https://creativecommons.org/licenses/by/4.0/

<u>Response to Reviewer #1</u>:

Comments:

This is a well-executed and thoughtfully designed study that addresses an important challenge in CNS drug delivery for neuroinflammatory diseases. The hybrid nanovesicle strategy combining microglia-derived membranes with siRNA-loaded liposomes is conceptually strong, and the authors provide extensive molecular, cellular, and *in vivo* data supporting therapeutic efficacy. The work is timely and of broad interest to the biomaterials and neuroimmunology communities. The comments below are intended to clarify several mechanistic interpretations and refine the strength of certain claims, which I believe will further strengthen the manuscript.

<u>Response:</u> Many thanks for your thoughtful evaluation of our work and for these constructive comments aimed at refining several mechanistic interpretations and calibrating the strength of certain claims. In response, we performed additional experiments, revised the relevant text and schematics, and carefully clarified the scope of our conclusions.

Comment 1: The authors repeatedly state "specific targeting of microglia." However, the data do not convincingly demonstrate that uptake by other brain-resident or infiltrating cell types (e.g., astrocytes, endothelial cells, neurons, peripheral immune cells) is minimal. At present, the results support preferential uptake by microglia rather than specificity. The authors should either temper the language or provide additional quantitative data demonstrating limited targeting of other cell populations.

<u>Response:</u> We appreciate you pointing out this overstatement. We agree that the original wording was overly strong. Based on the currently available evidence, our data support preferential uptake by microglia, rather than strict specificity. <u>Accordingly, we have revised the manuscript throughout to replace statements such as "specific targeting of microglia" with the more accurate description "preferential uptake by microglia"</u>.

Following your suggestion, <u>we performed further *in vitro* and *in vivo* experiments to quantitatively assess the distribution of siMETTL3-hNVs across different brain cell populations</u>. *In vitro*, siMETTL3-hNVs showed higher uptake in BV2 microglia than in C8-D1A astrocytes, HT22 neurons, and bEnd.3 endothelial cells (Supplementary Fig. 11). *In vivo*, siMETTL3-hNVs were formulated using Alexa Fluor 647-labeled siRNA and administered *via* a single intravenous injection, followed by flow cytometric analysis of dissociated brain cells at 12 h post-injection. siMETTL3-hNVs showed the highest uptake in microglia (14.97 ± 0.65%), followed by neurons (10.43 ± 0.51%), astrocytes (8.25 ± 1.67%), brain capillary endothelial cells (BCECs, 4.90 ± 1.33%) and infiltrating myeloid cells (3.67 ± 0.44%) (Fig. 3f, g; Supplementary Fig. 13), further supporting preferential uptake by microglia.

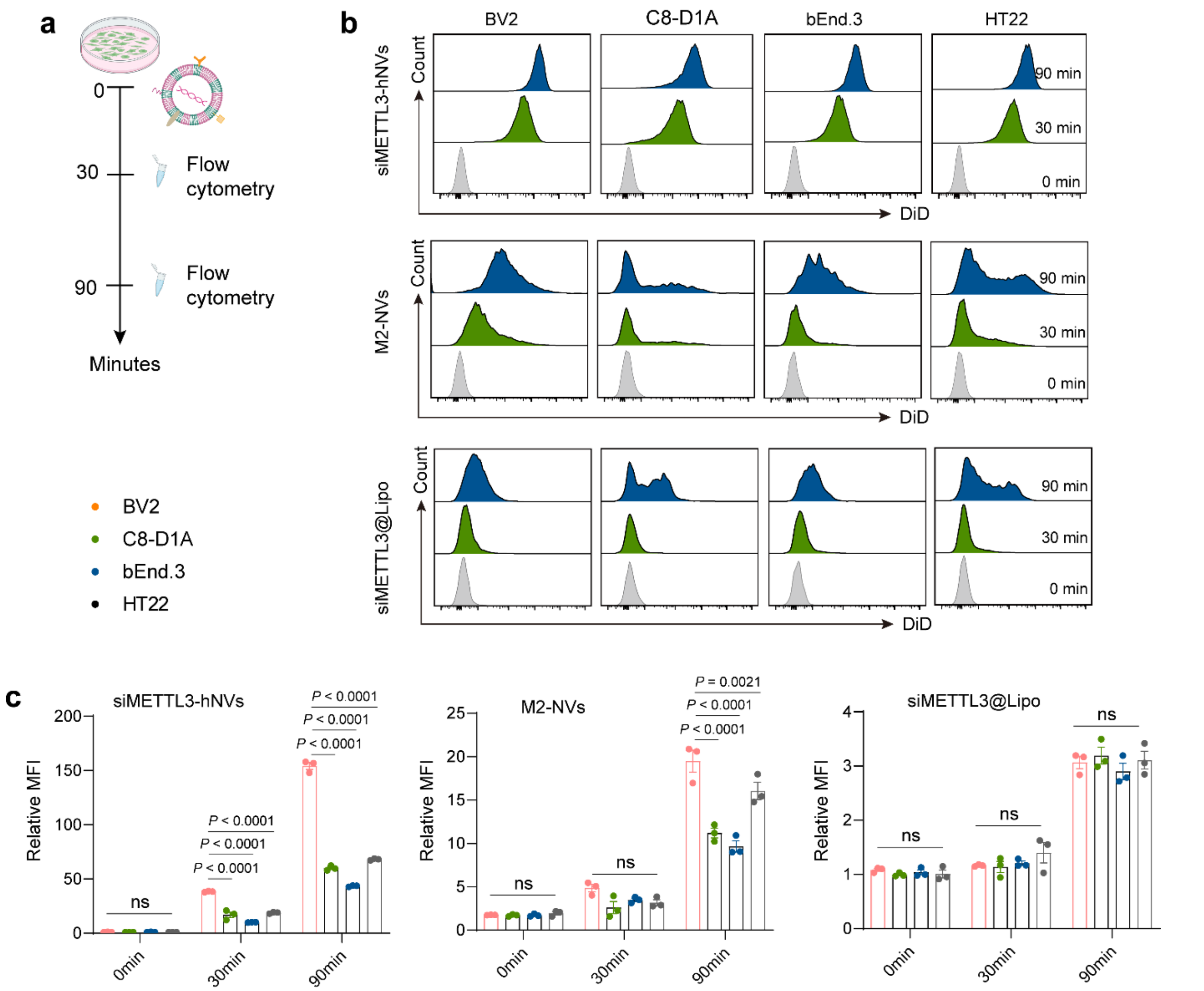

__Supplementary Fig. 11 | __In vitro cellular uptake of siMETTL3-hNVs by different brain-relevant cell types. __a,__ Schematic of cellular uptake by different cells treated with siMETTL3-hNVs. __b,__ Flow cytometry analysis of siMETTL3-hNVs uptake by BV2 microglia, C8-D1A astrocytes, HT22 neurons, and bEnd.3 endothelial cells after 30 or 90 min of incubation. __c,__ Quantitative analysis of mean fluorescence intensity (MFI) in the indicated cell types. MFI, mean fluorescence intensity. Data are presented as mean ± SEM (n = 3). Statistical significance was determined by two-way ANOVA followed by Tukey's multiple comparisons test.

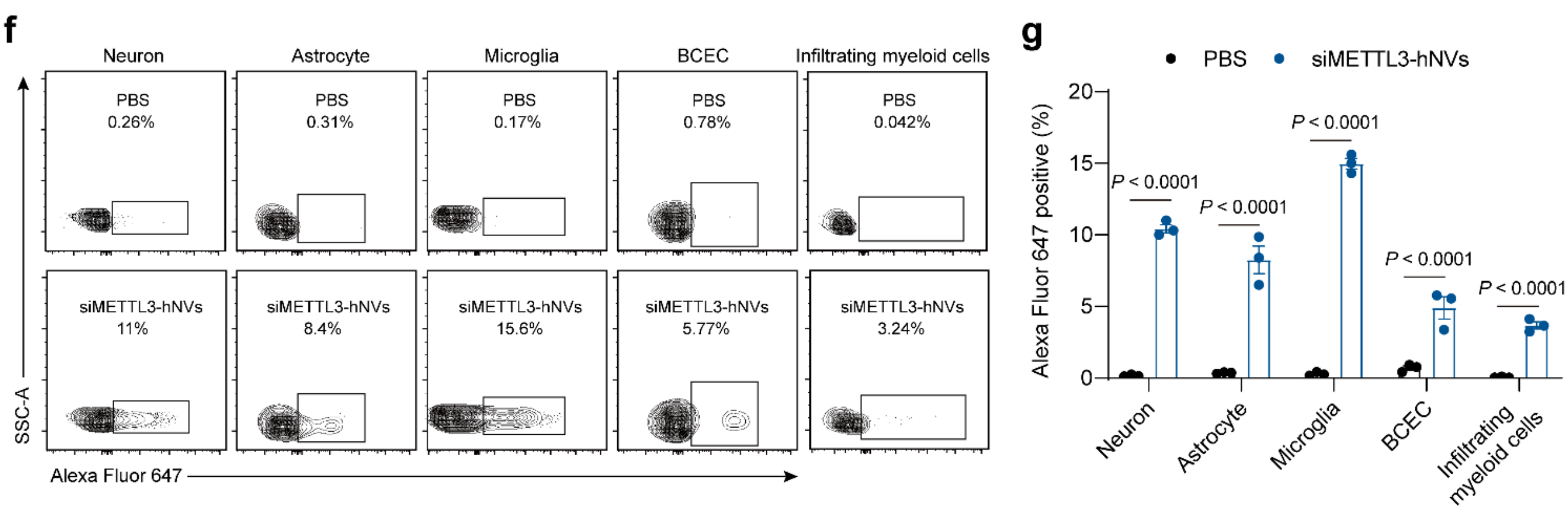

Fig. 3 | f-g, *Immunofluorescence flow cytometry analysis (**f**) and quantitative analysis (**g**) of the percentages of Alexa Fluor 647-positive cells among different brain cell populations in mice after intravenous administration of siMETTL3-hNVs.*

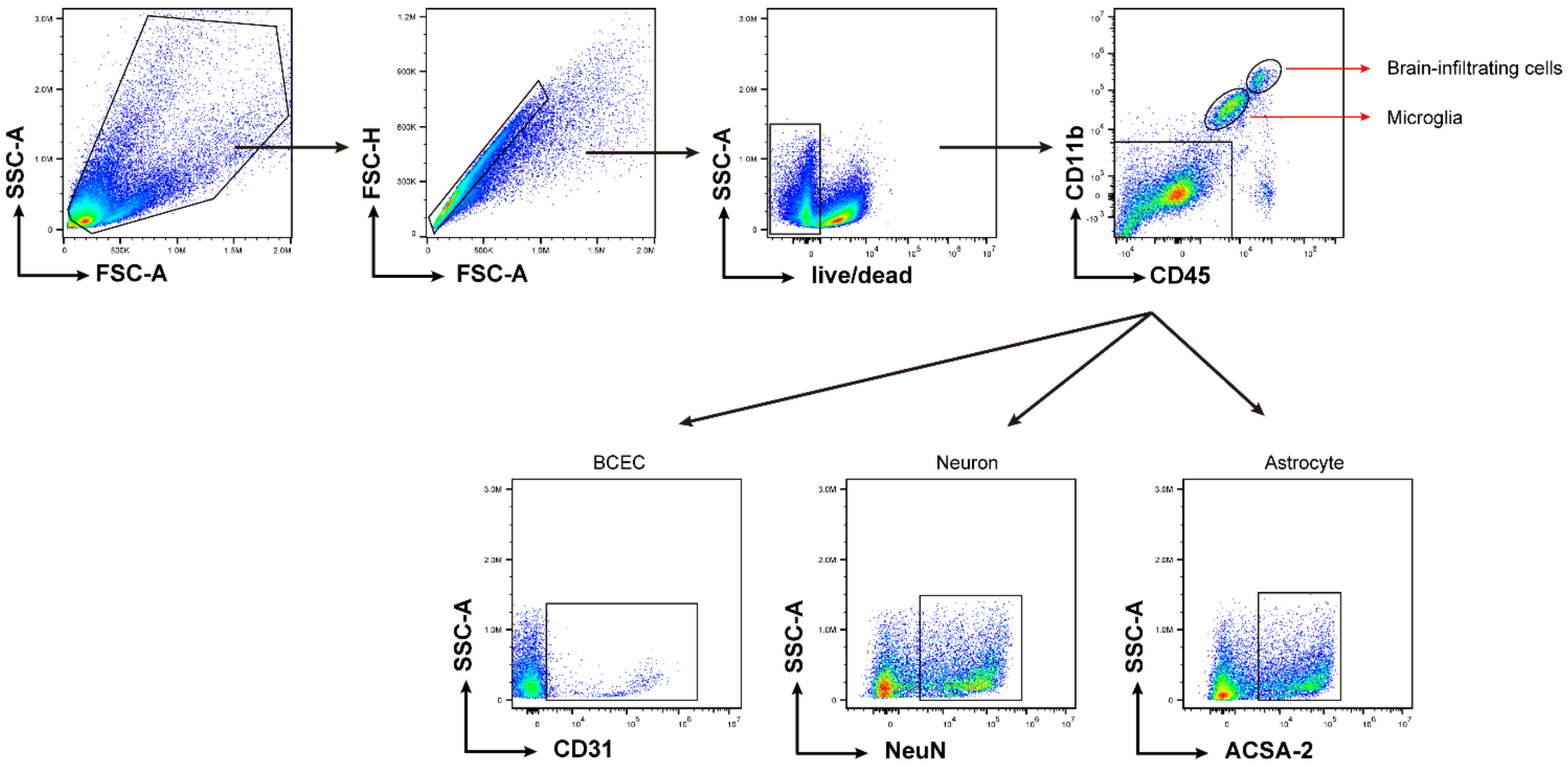

Supplementary Fig. 13 | *Gating scheme for immunofluorescence flow cytometry analysis of brain cells. The cell markers used for subsequent gating were: NeuN for neurons, ACSA-2 for astrocytes, CD11b for microglia, CD31 for BCECs.*

Materials and methods section:

"In vitro cellular uptake

To analyze cellular uptake, BV2, C8-D1A, HT22, and bEnd.3 cells seeded in 24-well plates were incubated with DiD-labeled siMETTL3-hNVs for 30 or 90 min. Cellular uptake was quantified by flow cytometry (CytoFLEX S, Beckman Coulter)."

"In vivo biodistribution and brain-cell distribution of siMETTL3-hNVs

For cargo-distribution analysis at the cellular level, siMETTL3-hNVs were formulated using Alexa Fluor 647-labeled siRNA and administered via a single intravenous injection. At 12 h post-injection, brains were harvested, dissociated into single-cell suspensions, and analyzed by flow cytometry to determine the percentage of Alexa Fluor 647-positive cells in different brain cell populations. Brain-cell flow cytometry was performed using the following panel: Live/Dead (BV510), CD45 (PerCP-Cy5.5), CD11b (FITC), CD31 (BV421), ACSA-2 (PE), NeuN (Vio 780), and Alexa Fluor 647-labeled siRNA (AF647)."

Results and discussion section:

"To initially explore whether siMETTL3-hNVs exhibit cell-type-dependent uptake, we first compared their internalization across several brain-relevant cell types in vitro. siMETTL3-hNVs showed higher uptake in BV2 microglia than in C8-D1A astrocytes, HT22 neurons, and bEnd.3 endothelial cells

(Supplementary Fig. 11)."

"We then evaluated cargo distribution in vivo by formulating siMETTL3-hNVs with Alexa Fluor 647-labeled siRNA, followed by a single intravenous injection and flow cytometric analysis of brain cell populations at 12 h post-injection. siMETTL3-hNVs showed the highest uptake in microglia (14.97 ± 0.65%), followed by neurons (10.43 ± 0.51%), astrocytes (8.25 ± 1.67%), brain capillary endothelial cells (BCECs, 4.90 ± 1.33%) and infiltrating myeloid cells (3.67 ± 0.44%) (Fig. 3f, g; Supplementary Fig. 13), indicating preferential uptake by microglia despite measurable delivery to other brain-resident cell populations."

Comment 2: The proposed CCR2-CCL2-mediated mechanism for BBB transmigration is conceptually unclear. While CCL2-CCR2 signaling is well established in immune cell chemotaxis during neuroinflammation, BBB crossing following systemic administration is primarily governed by endothelial transport mechanisms or barrier disruption. CCR2 is a microglial/immune cell receptor rather than a canonical BBB endothelial transcytosis receptor, making it difficult to reconcile how CCR2 inherited from microglial membranes directly mediates endothelial traversal. The presented data appear more consistent with enhanced post-BBB accumulation or inflammatory-site retention rather than CCR2-dependent BBB penetration per se. Additional experiments disentangling endothelial crossing from parenchymal homing (e.g., CCR2 blockade on vesicles or endothelial interaction assays) would be required to substantiate this mechanism.

Response: We appreciate your concern regarding the mechanistic interpretation of the CCR2–CCL2 axis. We agree that our original description did not sufficiently distinguish CCR2–CCL2 chemotaxis from BBB transport. We have therefore revised the manuscript to clarify that the CCR2–CCL2 axis is more appropriately interpreted as a mechanism promoting inflammatory-site homing and brain enrichment, rather than direct endothelial transcytosis.

To address this point, siMETTL3-hNVs were pretreated with the selective CCR2 antagonist RS504393. In the bEnd.3–BV2 Transwell model, RS504393 pretreatment significantly reduced the fluorescence signal in the lower chamber (Fig. 3h, i). Consistently, *in vivo* imaging showed that mice receiving RS504393-pretreated siMETTL3-hNVs exhibited markedly lower brain fluorescence intensity after intravenous injection(Fig. 3j, k). These data support a role for the CCR2–CCL2 axis in chemotactic enrichment under inflammatory conditions.

BBB transport fundamentally depends on the interaction between endothelial cells and nanocarriers[1]. This process may involve multiple internalization pathways, including phagocytosis, macropinocytosis, clathrin-mediated endocytosis, and caveolae-mediated endocytosis, depending on nanoparticle size, shape, surface functionalization, charge, and morphology[2]. To investigate how siMETTL3-hNVs are internalized by brain endothelial cells, we further assessed endothelial transport using inhibitors of major endocytic pathways. MβCD, a cholesterol-depleting agent commonly used to disrupt lipid rafts and caveolae-associated endocytosis, reduced the transcytosis efficiency of siMETTL3-hNVs by 72.92 ± 0.19%, whereas CPZ (a clathrin-mediated endocytic inhibitor) and EIPA

(a macropinocytosis inhibitor) showed no significant effect (Supplementary Fig. 20). In addition, MβCD significantly reduced fluorescence signals in both upper-chamber bEnd.3 cells and lower-chamber BV2 cells (Fig. 3i), supporting a major contribution of caveolae-associated transcytosis to endothelial transport *in vitro*. *In vivo*, MβCD pretreatment significantly reduced brain fluorescence intensity after injection of DiR-labeled siMETTL3-hNVs, and this effect was further enhanced when combined with RS504393 pretreatment (Fig. 3j, k). This interpretation is consistent with previous studies reporting caveolae-associated BBB transport of nanocarriers[3,4], which may be facilitated by the abundance of caveolae in endothelial cells[5] as well as by PEGylation[6] and membrane-derived surface components[7] that favor caveolae-associated uptake.

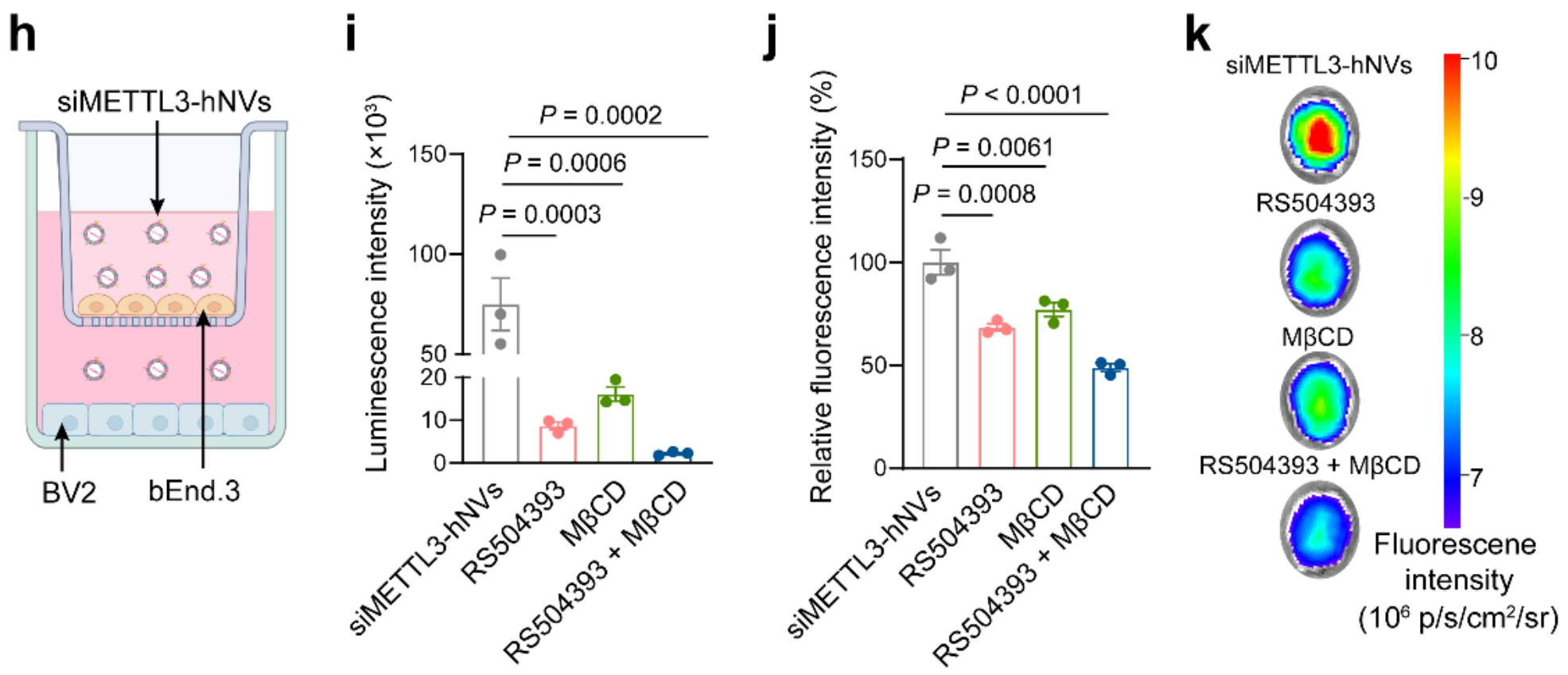

Fig. 3* | *h, *Schematic illustration of the bEnd.3–BV2 Transwell assay for BBB penetration assessment, with BV2 cells seeded in the lower chamber.* ***i,*** *Fluorescence intensity of BV2 cells in the lower chamber after treatment with siMETTL3-hNVs, including RS504393-pretreated siMETTL3-hNVs and MβCD-pretreated bEnd.3 cells.* ***j-k,*** *Quantification of brain fluorescence intensity* ***(j)*** *and representative IVIS images* ***(k)*** *after intravenous injection of siMETTL3-hNVs, including mice pretreated with MβCD or receiving RS504393-pretreated siMETTL3-hNVs.*

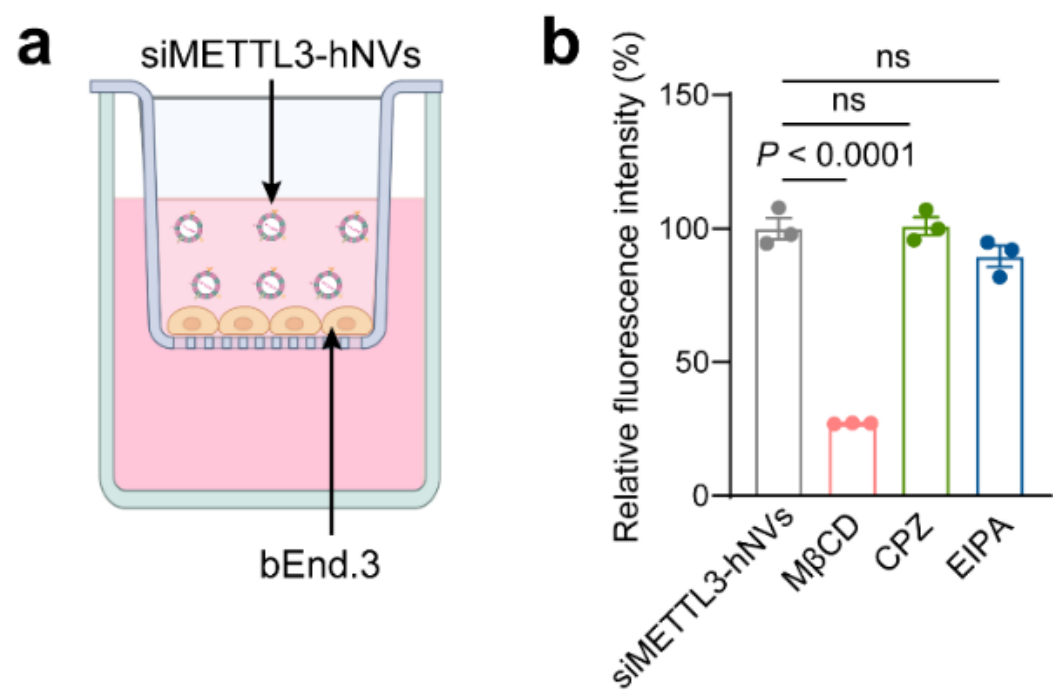

Supplementary Fig. 20* | *a, *Schematic illustration of the bEnd.3 Transwell assay for BBB penetration assessment.* ***b,*** *Relative fluorescence intensity of siMETTL3-hNVs in the Transwell system after pretreatment of bEnd.3 cells with different endocytic inhibitors, normalized to the siMETTL3-hNVs group. Data are expressed as mean ± SEM (n = 3). Statistical significance was determined using one-way ANOVA followed by Tukey's multiple comparisons test.*

Reference:

1. G.C. Terstappen, Meyer, A.H., Bell, R.D. & Zhang, W. Strategies for delivering therapeutics across the blood-brain barrier. *Nat. Rev. Drug Discov.* 20, 362-383 (2021)

2. M. Sousa de Almeida, et al. Understanding nanoparticle endocytosis to improve targeting strategies in nanomedicine. *Chem. Soc. Rev.* 50, 5397-5434 (2021)

3. C. Wang, et al. Blood-brain-barrier-crossing lipid nanoparticles for mRNA delivery to the central nervous system. *Nat. Mater.* 24, 1653-1663 (2025)

4. Z. Wang, et al. A sphingolipid-derived paclitaxel nanovesicle enhances efficacy of combination therapies in triple-negative breast cancer and pancreatic cancer. *Nat. Cancer* 6, 1734-1753 (2025)

5. M. Gumbleton, Abulrob, A.G. & Campbell, L. Caveolae: an alternative membrane transport compartment. *Pharm. Res.* 17, 1035-1048 (2000)

6. C. Brandenberger, et al. Quantitative evaluation of cellular uptake and trafficking of plain and polyethylene glycol-coated gold nanoparticles. *Small* 6, 1669-1678 (2010)

7. S.T. Chiang, et al. Biomimetic Nanovesicles Synergize with Short-Term Fasting for Enhanced Chemotherapy of Triple-Negative Breast Cancer. *ACS Nano* 18, 33875-33889 (2024)

Materials and methods section:

*"**In vitro BBB Transwell model and endocytic inhibition assays***

To investigate the mechanism of transendothelial transport, bEnd.3 cells were pretreated with MβCD (4.5 mM), CPZ (5 μg/mL), or EIPA (10 μM) for 30 min at 37 °C, respectively, before addition of siMETTL3-hNVs. To assess the contribution of the CCR2–CCL2 axis, siMETTL3-hNVs were pretreated with RS504393 (10 μM) for 30 min at 37 °C before being applied to the upper chamber. Transendothelial transport was then quantified by flow cytometry analysis of lower-chamber BV2 cells."

*"**In vivo inhibitor validation and inflammation-dependent brain delivery studies***

For in vivo inhibitor validation, mice received an intraperitoneal injection of MβCD (100 mg/kg) 1 h before intravenous administration of DiR-labeled siMETTL3-hNVs. In separate experiments, siMETTL3-hNVs were pretreated with RS504393 before injection. Brain fluorescence was quantified by IVIS imaging at 12 h post-injection, followed by ex vivo IVIS imaging of isolated brains."

Results and discussion section:

"To assess the contribution of the CCR2–CCL2 axis, a bEnd.3–BV2 Transwell model under inflammatory conditions was established as described previously (Supplementary Fig. 18)[40], and we found that pretreatment of siMETTL3-hNVs with RS504393 (a selective CCR2 antagonist) significantly reduced the fluorescence signal in the lower chamber (Fig. 3h, i, Supplementary Fig. 19). Consistently, in vivo imaging showed that mice receiving RS504393-pretreated siMETTL3-hNVs exhibited markedly lower brain fluorescence intensity after intravenous injection (Fig. 3j, k). These results support the interpretation that the CCR2-CCL2 axis acts as a chemotactic cue that promotes inflammatory-site homing and brain enrichment of siMETTL3-hNVs.

BBB translocation depends on the interaction between endothelial cells and nanocarriers and

may involve multiple uptake pathways determined by nanoparticle size, shape, surface chemistry, charge, and morphology[41,42]. In the bEnd.3-BV2 Transwell model, methyl-β-cyclodextrin (MβCD, a caveolae-mediated endocytic inhibitor) reduced the transcytosis efficiency of siMETTL3-hNVs by 72.92 ± 0.19%, whereas chlorpromazine (CPZ, a clathrin-mediated endocytic inhibitor) and 5-(N-ethyl-N-isopropyl)-amiloride (EIPA, a micropinocytosis-mediated endocytic inhibitor) showed no significant effect (Supplementary Fig. 20). This result suggests that endothelial transport of siMETTL3-hNVs may preferentially rely on a caveolae-associated pathway. MβCD also significantly decreased fluorescence signals in both upper-chamber bEnd.3 cells and lower-chamber BV2 cells (Fig. 3i), and pretreatment of mice with MβCD similarly reduced brain fluorescence intensity after injection of DiR-labeled siMETTL3-hNVs in vivo, with a further reduction observed when combined with RS504393-pretreated siMETTL3-hNVs (Fig. 3j, k). Together, these findings support that caveolae might be critical mediators in facilitating the BBB crossing of siMETTL3-hNVs, in line with previous studies[43,44].”

Comment 3: If the CCR2–CCL2 axis is a key driver of brain targeting and BBB penetration, an important prediction is that vesicle accumulation in the brain should be low under healthy conditions where CCL2 expression is minimal. Quantitative biodistribution data in healthy (non-inflamed) mice would therefore be informative. In addition, examining how vesicle accumulation scales with the degree of neuroinflammation (e.g., across different LPS doses or time points) would help establish whether targeting efficiency correlates with CCL2 levels, as implied by the proposed chemokine-gradient mechanism.

Response: We thank you for this valuable suggestion. As requested, we performed additional *in vivo* experiments comparing brain accumulation of siMETTL3-hNVs in healthy mice and in LPS-induced neuroinflammatory mice with different degrees of inflammation. As expected, brain accumulation of siMETTL3-hNVs was markedly lower in healthy mice than in inflamed mice. Moreover, with increasing LPS dose, brain fluorescence intensity increased in a dose-dependent manner, indicating progressively enhanced accumulation of siMETTL3-hNVs under stronger inflammatory conditions (Supplementary Fig. 21a, b).

To further examine the relationship between vesicle accumulation and inflammatory signaling, we also measured CCL2 levels in brain tissues from these mice. Notably, brain CCL2 levels increased in parallel with the severity of LPS-induced neuroinflammation, and this trend was consistent with the enhanced brain accumulation of siMETTL3-hNVs (Supplementary Fig. 21c). These results support the interpretation that brain enrichment of siMETTL3-hNVs is associated with the inflammatory state and correlates with CCL2 upregulation, consistent with a chemokine-gradient-associated homing mechanism.

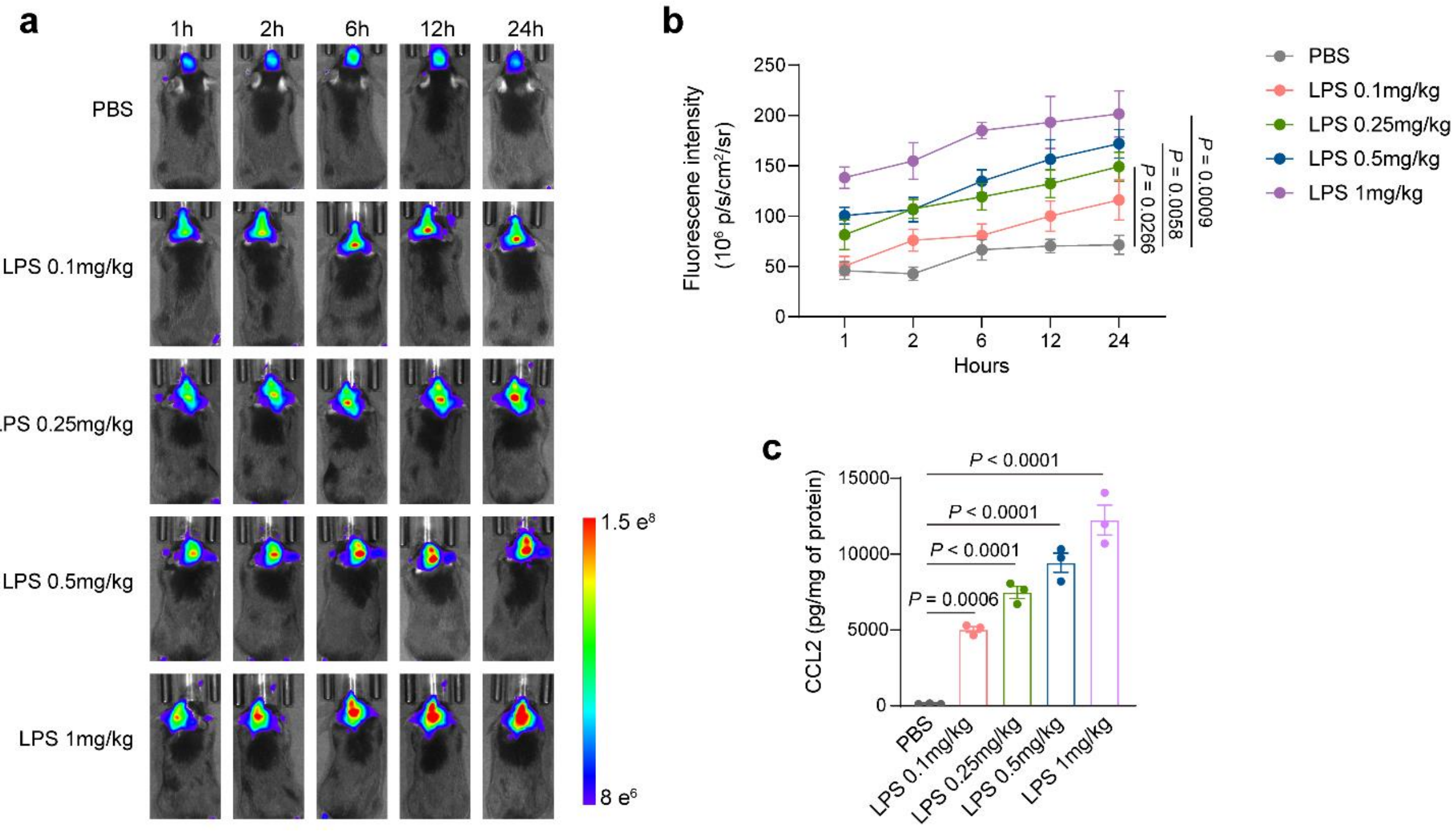

***Supplementary Fig. 21 | a,** In vivo fluorescence imaging of healthy mice and LPS-induced neuroinflammatory mice receiving PBS or siMETTL3-hNVs at different time points. **b,** Quantitative analysis of brain fluorescence intensity from panel **a** at the indicated time points. **c,** ELISA analysis of CCL2 levels in brain tissues under different inflammatory conditions. Data are expressed as mean ± SEM (n = 3). Statistical significance was determined using one-way ANOVA (**c**) or two-way ANOVA (**b**) followed by Tukey's multiple comparisons test.*

Materials and methods section:

*"**In vivo inhibitor validation and inflammation-dependent brain delivery studies***

For inflammation-dependent brain delivery studies, mice were assigned to a healthy control group or to neuroinflammatory groups treated with LPS at 0.1, 0.25, 0.5, or 1 mg/kg by intraperitoneal injection once daily for 7 consecutive days. Fluorescently labeled siMETTL3-hNVs were then intravenously administered, and brain accumulation was quantified by IVIS imaging. Brain tissues were collected for CCL2 ELISA."

Results and discussion section:

"To further evaluate the inflammation dependence of this process, we compared brain accumulation of siMETTL3-hNVs in healthy mice and LPS-induced neuroinflammatory mice with different degrees of inflammation. Brain accumulation was markedly lower in healthy mice and increased with inflammatory severity, in parallel with elevated CCL2 levels in brain tissue (Supplementary Fig. 21). These results are consistent with a CCL2-related chemokine-guided enrichment process."

Response to Reviewer #2:

Comments:

In this manuscript, Xu et al. constructed a hybrid epigenetic nanomodulator, siMETTL3-hNVs, by integrating natural M2 microglial-derived nanovesicles (NVs) with synthetic liposomes to deliver siRNA targeting the m6A writer METTL3 for neuroinflammation therapy. By leveraging the native CCR2-CCL2 axis inherited from the parent microglial membranes, the system can efficiently cross the BBB and preferentially accumulate in inflamed brain regions. The authors demonstrate a dual-modality therapeutic mechanism: the NVs act as molecular decoys to neutralize pro-inflammatory cytokines while the siRNA intracellularly silences METTL3 to prevent the m6A-mediated degradation of SOCS3 mRNA. This stabilization of SOCS3 potently inhibits the JAK2-STAT3 signaling pathway, effectively driving microglial repolarization from a pro-inflammatory M1 state to a restorative M2 phenotype. The efficacy of this platform was validated in mouse models of LPS-induced neuroinflammation and RIBI. I have the following concerns:

Response: We sincerely thank you for the careful evaluation of our study and for the thoughtful comments regarding the mechanistic framing of the manuscript. We particularly appreciate your suggestions on clarifying the role of SOCS3, refining the schematic descriptions, and improving the precision of several mechanistic interpretations. In response, we have revised the Introduction and corresponding figures, moderated several statements, and incorporated additional experiments to strengthen support for the revised interpretation.

Comment 1: In the Introduction, the authors should provide a more comprehensive background on the specific role of SOCS3 within the inflammatory signaling cascade and explain its pivotal importance in guiding the design of the epigenetic nanomodulator therapy. Currently, the manuscript briefly mentions "intracellularly restoring SOCS3 stability *via* METTL3 knockdown", but it does not fully articulate why SOCS3 is a primary target for resolving neuroinflammation. The authors should also provide the full names of these genes and proteins before using their respective abbreviations.

Response: We appreciate your suggestion to strengthen the mechanistic context of the Introduction. To address this point, we revised the second paragraph of the Introduction to more clearly explain why suppressor of cytokine signaling 3 (SOCS3) is mechanistically relevant to our therapeutic design. In the revised text, we now state that SOCS3 is a key endogenous feedback inhibitor of inflammatory signaling that constrains Janus kinase 2/signal transducer and activator of transcription 3 (JAK2–STAT3) activation in myeloid cells, including microglia, thereby limiting neuroinflammatory amplification[1,2]. We further incorporated literature showing that N6-methyladenosine (m^6A)-dependent regulation can modulate SOCS-family[3] signaling, and that a recently reported alkB homolog 5, RNA demethylase (ALKBH5)–m6A–SOCS3 axis in microglia/macrophages alleviates neuroinflammation after brain injury by stabilizing SOCS3 transcripts and suppressing JAK2–STAT3 signaling[4]. Together, these additions clarify why SOCS3 represents a biologically relevant downstream effector linking METTL3-dependent epitranscriptomic regulation to neuroinflammatory control. In

addition, the full names of the relevant genes and proteins are now provided at first mention in the revised manuscript.

Reference:

1. M. Yan, et al. SOCS modulates JAK-STAT pathway as a novel target to mediate the occurrence of neuroinflammation: Molecular details and treatment options. *Brain Res. Bull.* 213, 110988 (2024)
2. H. Qin, et al. Signal transducer and activator of transcription-3/suppressor of cytokine signaling-3 (STAT3/SOCS3) axis in myeloid cells regulates neuroinflammation. *Proc. Natl. Acad. Sci. U. S. A.* 109, 5004-5009 (2012)
3. H.B. Li, et al. m^6A mRNA methylation controls T cell homeostasis by targeting the IL-7/STAT5/SOCS pathways. *Nature* 548, 338-342 (2017)
4. L. Cai, et al. ALKBH5 demethylates the m^6A modification of SOCS3 in microglia/macrophages and alleviates neuroinflammation after brain injury. *Proc. Natl. Acad. Sci. U. S. A.* 122, e2504697122 (2025)

Introduction section:

"Notably, suppressor of cytokine signaling 3 (SOCS3) is a key endogenous feedback inhibitor of inflammatory signaling that constrains Janus kinase 2/signal transducer and activator of transcription 3 (JAK2–STAT3) activation in myeloid cells, including microglia, thereby limiting neuroinflammatory amplification[18,19]. Emerging evidence further suggests that m6A-dependent regulation can modulate SOCS-family signaling[20], and a recent study identified an ALKBH5–m^6A–SOCS3 axis in microglia/macrophages that alleviates neuroinflammation after brain injury by stabilizing SOCS3 transcripts and suppressing JAK2–STAT3 signaling[21]. Together, these findings position SOCS3 as a key downstream effector linking epitranscriptomic regulation to neuroinflammatory control."

Comment 2: In Page 8, lines 181–184, the authors indicate that "activated microglia as a principal source of this chemokine during neuroinflammation". However, this conclusion is not sufficiently supported as the experiments only tested BV2 microglial cells following LPS stimulation. Since the study does not include comparative expression data from other brain cells, such as astrocytes or neurons, the claim that microglia are the principal source is not solid. The authors should evaluate the chemokine production levels in other relevant brain cell lines to determine the relative contribution of microglia compared to other cell types

Response: We thank you for pointing out this overstatement. Per your suggestion, we performed additional *in vitro* experiments comparing CCL2 production across several brain-relevant cell types, including BV2 microglia, bEnd.3 endothelial cells, HT22 neurons, and C8-D1A astrocytes, following stimulation with LPS (100 ng/mL). CCL2 levels in the culture supernatants were then quantified by ELISA. Among the tested cell types, BV2 microglial cells exhibited the highest level of CCL2 secretion after LPS stimulation, whereas the other cell types produced substantially lower levels (Supplementary Fig. 16). We have therefore revised the manuscript accordingly and now describe microglia as a major contributor to CCL2 production among the tested brain-relevant cell types under

our experimental conditions.

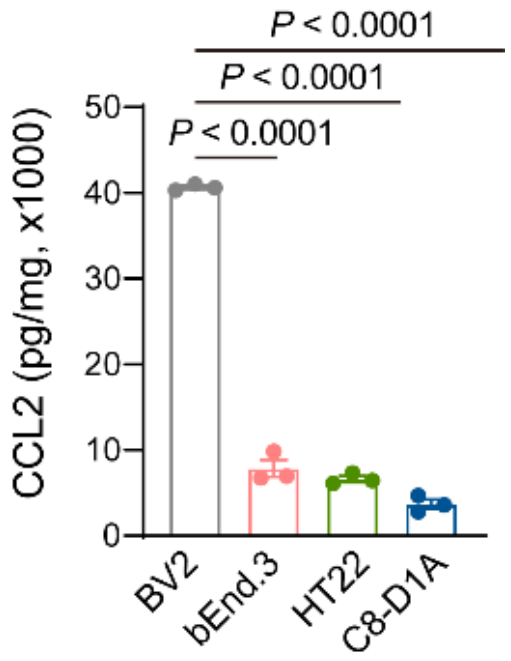

Supplementary Fig. 16 | *ELISA analysis of CCL2 levels in the culture supernatants of BV2, bEnd.3, HT22, and C8-D1A cells after LPS stimulation (100 ng/mL). Data are expressed as mean ± SEM (n = 3). Statistical significance was determined using one-way ANOVA followed by Tukey's multiple comparisons test.*

Materials and methods section:

*"**CCL2 secretion assay in different brain-relevant cell types***

To compare the inflammatory production of CCL2 across different brain-relevant cell types, BV2, bEnd.3, HT22, and C8-D1A cells were cultured under identical conditions and stimulated with LPS (100 ng/mL) for 24 h. Culture supernatants were collected, clarified by centrifugation, and analyzed using a commercial CCL2 ELISA kit according to the manufacturer's instructions. Cytokine levels were normalized to total cellular protein content determined by BCA assay."

Results and discussion section:

"Consistent with this, ELISA analysis showed that, among BV2 microglia, bEnd.3 endothelial cells, HT22 neurons and C8-D1A astrocytes, BV2 cells secreted the highest level of CCL2 after LPS stimulation (Supplementary Fig. 16), supporting microglia as a major contributor to CCL2 production under our experimental conditions."

Comment 3: In Figure 1 and Figure 3o, the mechanistic illustration suggests that the nanomodulators utilize the CCR2-CCL2 axis by binding circulating CCL2 to facilitate blood-brain barrier (BBB) transport, followed by CCL2 detachment. However, several points require further clarification:

Response: We thank you for this important comment. The original schematics and related text were not sufficiently precise and may have led to misunderstanding. We have revised them accordingly, and the specific points are addressed below.

Comment 3.1: There is no experimental evidence provided to support the claim that CCL2 detaches from the nanoparticles after crossing the BBB.

Response: We apologize for the confusion caused by the original schematic illustrations. In the original schematic, the interaction between siMETTL3-hNVs and CCL2 was not explicitly shown after

BBB crossing, which may have led to the mistaken impression that CCL2 dissociated during the transport process. Our intended model was that siMETTL3-hNVs undergo chemotactic enrichment toward the inflamed brain through the CCR2–CCL2 interaction, without implying a separate dissociation step. We have now revised Figure 1 and Figure 3 to explicitly depict this interaction and to avoid the previous misunderstanding. The corresponding schematic originally shown as Fig. 3o has been revised and is now presented as Fig. 3l.

REDACTED 3PM

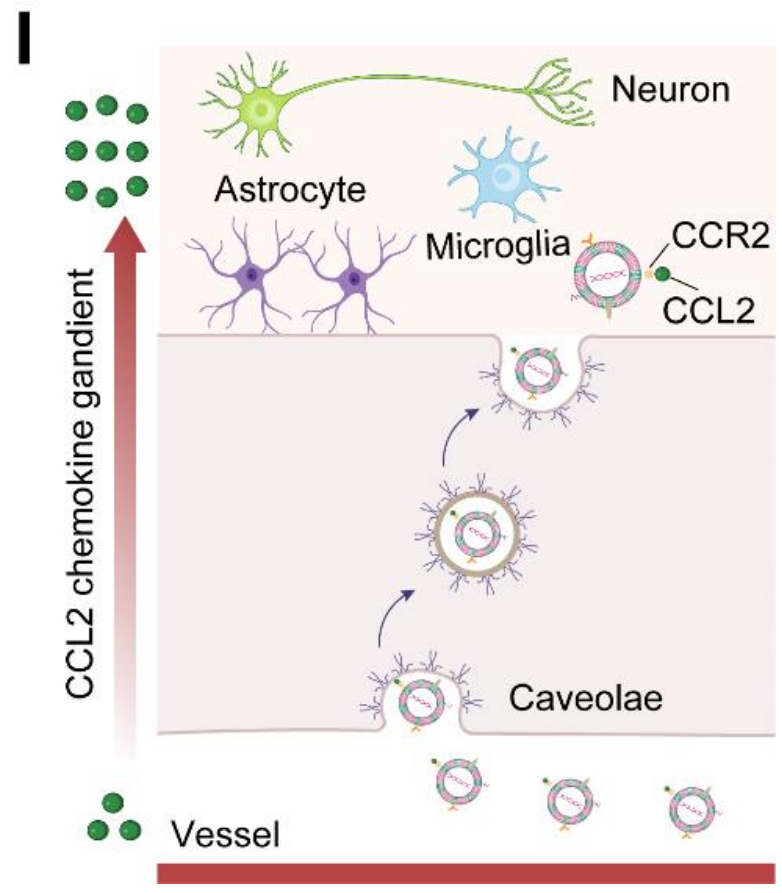

Fig. 3 | l, Schematic diagram illustrating the proposed mechanism underlying brain delivery of siMETTL3-hNVs, involving CCR2-CCL2 chemotactic enrichment, caveolae-mediated transcytosis across the BBB, and preferential uptake by microglia.

Comment 3.2: The specific cellular mechanism by which the nanoparticles use the CCR2-CCL2 axis to traverse endothelial layers remains undefined.

Response: Thank you very much for this professional and insightful comment. We appreciate your point that the specific cellular mechanism underlying endothelial traversal required further clarification. Importantly, our revised interpretation is that the CCR2–CCL2 axis does not directly define the cellular route of endothelial traversal, but instead primarily contributes to chemotactic enrichment and thereby facilitates the inflamed-brain delivery of siMETTL3-hNVs. To clarify this distinction, we performed additional experiments to separate the contribution of CCR2–CCL2-associated chemotactic signaling from that of endothelial transport, using the bEnd.3–BV2 Transwell model.

In this setting, pretreatment of siMETTL3-hNVs with RS504393, a selective CCR2 antagonist, significantly reduced the fluorescence signal detected in the lower chamber, supporting the interpretation that the CCR2–CCL2 axis promotes particle enrichment toward the inflammatory side of the model (Fig. 3h, i).

We then focused on the cellular mechanism of endothelial transport. Because transendothelial transport of nanocarriers may involve multiple endocytic pathways depending on nanoparticle size, surface chemistry, and membrane properties[1,2], we examined the effects of inhibitors of caveolae-associated endocytosis (MβCD), clathrin-mediated endocytosis (CPZ), and macropinocytosis (EIPA). Among these, MβCD reduced the transcytosis efficiency of siMETTL3-hNVs by 72.92 ± 0.19%, whereas CPZ and EIPA did not produce significant inhibition. In addition, MβCD pretreatment significantly decreased the fluorescence signals in both upper-chamber bEnd.3 cells and lower-chamber BV2 cells (Fig. 3i; Supplementary Fig. 20). These findings support a major contribution of caveolae-associated transcytosis to endothelial transport of siMETTL3-hNVs in our *in vitro* BBB model. This interpretation is consistent with previous studies reporting caveolae-associated BBB transport of nanocarriers[3,4], which may be facilitated by the abundance of caveolae in endothelial cells[5] as well as by PEGylation[6] and membrane surface components[7] that favor caveolae-associated uptake.

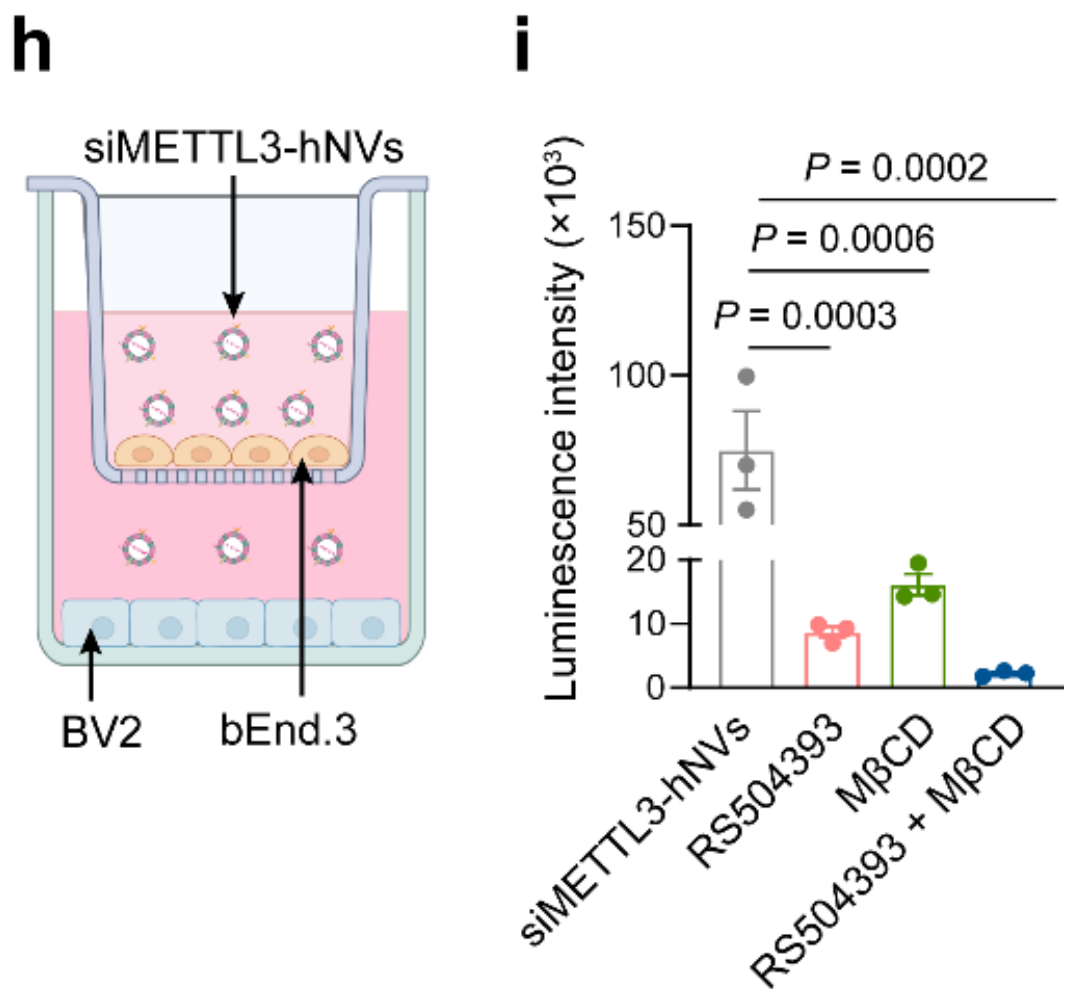

Fig. 3* | *h, *Schematic illustration of the bEnd.3–BV2 Transwell assay for BBB penetration assessment, with BV2 cells seeded in the lower chamber.* ***i,*** *Fluorescence intensity of BV2 cells in the lower chamber after treatment with siMETTL3-hNVs, including RS504393-pretreated siMETTL3-hNVs and MβCD-pretreated bEnd.3 cells.*

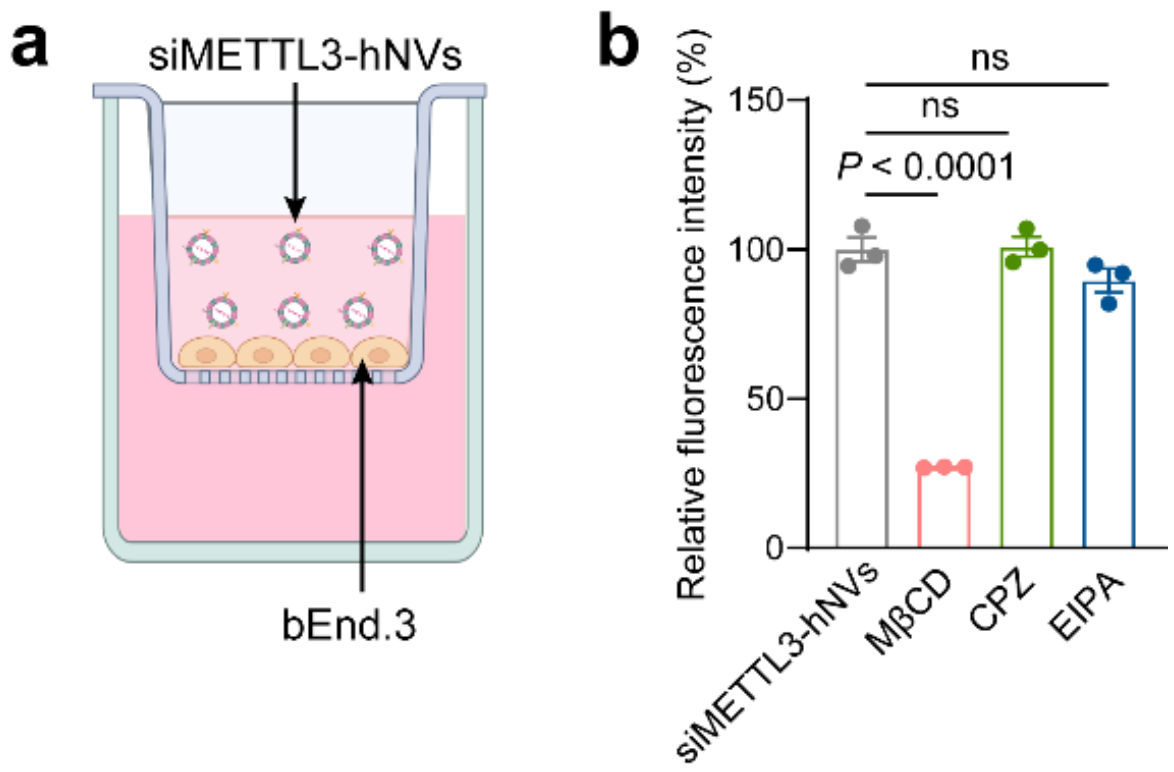

Supplementary Fig. 20* | *a, *Schematic illustration of the bEnd.3 Transwell assay for BBB penetration assessment.* ***b,*** *Relative fluorescence intensity of siMETTL3-hNVs in the Transwell system after pretreatment of bEnd.3 cells with different endocytic inhibitors, normalized to the siMETTL3-hNVs group. Data are expressed as mean ± SEM (n = 3). Statistical significance was determined using one-way ANOVA followed by Tukey's multiple comparisons test.*

Reference:

1. G.C. Terstappen, Meyer, A.H., Bell, R.D. & Zhang, W. Strategies for delivering therapeutics across the blood-brain barrier. *Nat. Rev. Drug Discov.* 20, 362-383 (2021)

2. M. Sousa de Almeida, et al. Understanding nanoparticle endocytosis to improve targeting strategies in nanomedicine. *Chem. Soc. Rev.* 50, 5397-5434 (2021)

3. C. Wang, et al. Blood-brain-barrier-crossing lipid nanoparticles for mRNA delivery to the central nervous system. *Nat. Mater.* 24, 1653-1663 (2025)

4. Z. Wang, et al. A sphingolipid-derived paclitaxel nanovesicle enhances efficacy of combination therapies in triple-negative breast cancer and pancreatic cancer. *Nat. Cancer* 6, 1734-1753 (2025)

5. M. Gumbleton, Abulrob, A.G. & Campbell, L. Caveolae: an alternative membrane transport

compartment. *Pharm. Res.* 17, 1035-1048 (2000)
6. C. Brandenberger, et al. Quantitative evaluation of cellular uptake and trafficking of plain and polyethylene glycol-coated gold nanoparticles. *Small* 6, 1669-1678 (2010)
7. S.T. Chiang, et al. Biomimetic Nanovesicles Synergize with Short-Term Fasting for Enhanced Chemotherapy of Triple-Negative Breast Cancer. *ACS Nano* 18, 33875-33889 (2024)

Materials and methods section:

*"**In vitro BBB Transwell model and endocytic inhibition assays***

To investigate the mechanism of transendothelial transport, bEnd.3 cells were pretreated with MβCD (4.5 mM), CPZ (5 μg/mL), or EIPA (10 μM) for 30 min at 37 °C, respectively, before addition of siMETTL3-hNVs. To assess the contribution of the CCR2–CCL2 axis, siMETTL3-hNVs were pretreated with RS504393 (10 μM) for 30 min at 37 °C before being applied to the upper chamber. Transendothelial transport was then quantified by flow cytometry analysis of lower-chamber BV2 cells."

Results and discussion section:

"To assess the contribution of the CCR2–CCL2 axis, a bEnd.3-BV2 Transwell model under inflammatory conditions was established as described previously (Supplementary Fig. 18)[40], and we found that pretreatment of siMETTL3-hNVs with RS504393 (a selective CCR2 antagonist) significantly reduced the fluorescence signal in the lower chamber (Fig. 3h, i, Supplementary Fig. 19)."

"BBB translocation depends on the interaction between endothelial cells and nanocarriers and may involve multiple uptake pathways determined by nanoparticle size, shape, surface chemistry, charge, and morphology[41,42]. In the bEnd.3-BV2 Transwell model, methyl-β-cyclodextrin (MβCD, a caveolae-mediated endocytic inhibitor) reduced the transcytosis efficiency of siMETTL3-hNVs by 72.92 ± 0.19%, whereas chlorpromazine (CPZ, a clathrin-mediated endocytic inhibitor) and 5-(N-ethyl-N-isopropyl)-amiloride (EIPA, a micropinocytosis-mediated endocytic inhibitor) showed no significant effect (Supplementary Fig. 20). This result suggests that endothelial transport of siMETTL3-hNVs may preferentially rely on a caveolae-associated pathway."

Comment 3.3: The authors should validate this proposed crossing mechanism *in vivo* using inhibitory assays, such as targeting CCL2 production or its receptors on endothelial layers, or by using comparative models involving healthy versus inflamed mice.

Response: We appreciate your valuable suggestion. Building on the *in vitro* findings described above, we further performed *in vivo* experiments to validate the revised mechanism. To assess the contribution of the CCR2–CCL2 axis *in vivo*, siMETTL3-hNVs were pretreated with RS504393, a selective CCR2 antagonist, prior to intravenous injection. Mice receiving RS504393-pretreated siMETTL3-hNVs exhibited markedly reduced brain fluorescence intensity, supporting a role for the CCR2–CCL2 axis in brain enrichment under inflammatory conditions. To further evaluate the endothelial transport component *in vivo*, mice were pretreated with MβCD before injection of DiR-labeled siMETTL3-

hNVs, which likewise significantly reduced brain fluorescence intensity. Notably, this reduction became more pronounced when MβCD pretreatment was combined with RS504393-pretreated siMETTL3-hNVs, further supporting that chemotactic enrichment and endothelial transcytosis likely contribute through partly distinct processes to the inflamed-brain delivery of siMETTL3-hNVs (Fig. 3j, k).

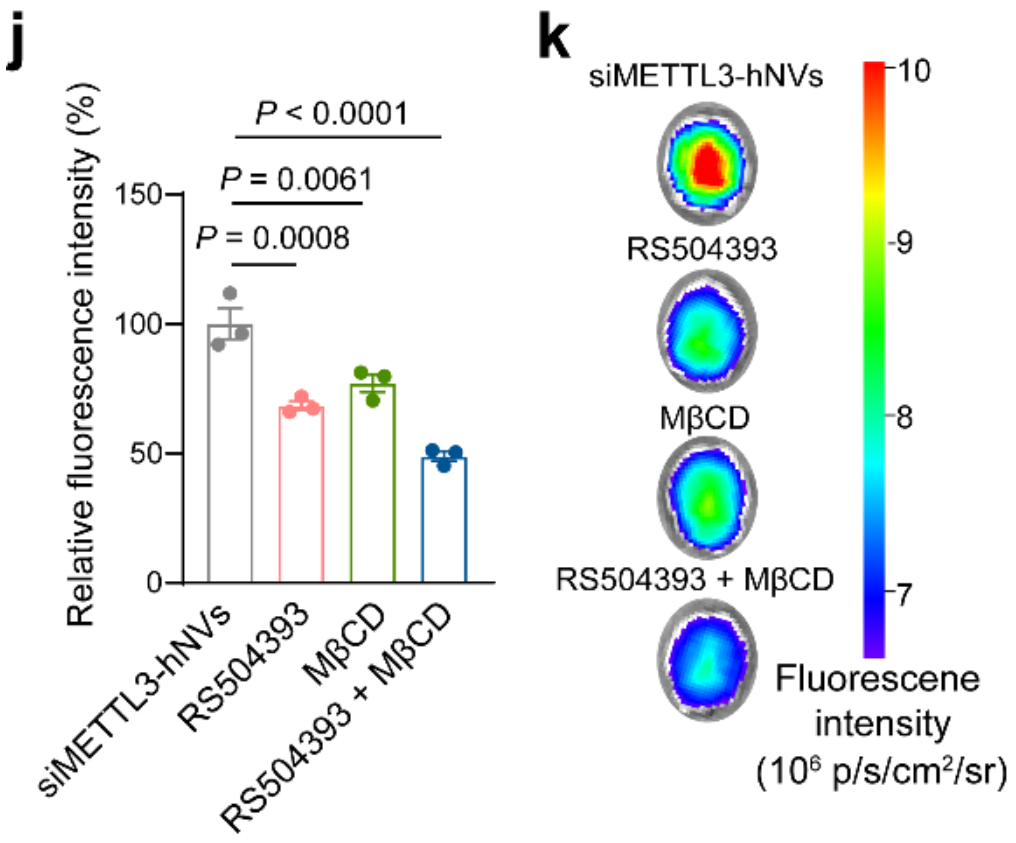

Fig. 3 | j-k, *Quantification of brain fluorescence intensity (**j**) and representative IVIS images (**k**) after intravenous injection of siMETTL3-hNVs, including mice pretreated with MβCD or receiving RS504393-pretreated siMETTL3-hNVs.*

In addition, we compared brain accumulation of siMETTL3-hNVs in healthy mice and in LPS-induced neuroinflammatory mice with different degrees of inflammation. Brain accumulation was markedly lower in healthy mice, and increased progressively with inflammatory severity, in parallel with elevated CCL2 levels in brain tissue (Supplementary Fig. 21). Together, these *in vivo* results support a revised model in which the CCR2–CCL2 axis primarily promotes inflammatory-site homing and brain enrichment, whereas MβCD-sensitive endothelial transport contributes to BBB traversal.

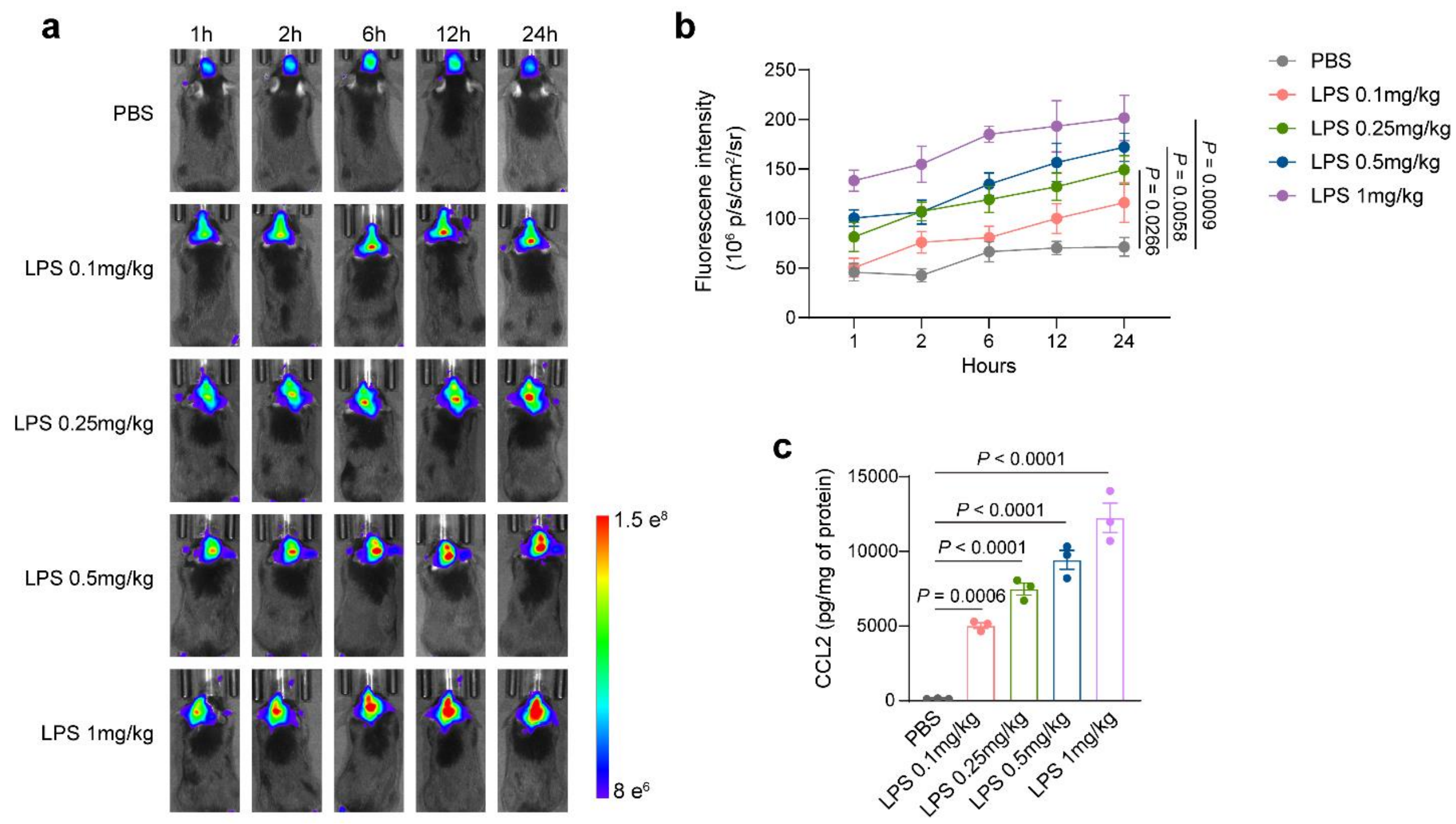

***Supplementary Fig. 21 | a**, In vivo fluorescence imaging of healthy mice and LPS-induced neuroinflammatory mice receiving PBS or siMETTL3-hNVs at different time points. **b,** Quantitative analysis of brain fluorescence intensity from panel **a** at the indicated time points. **c,** ELISA analysis of CCL2 levels in brain tissues under different inflammatory conditions. Data are expressed as mean ± SEM (n = 3). Statistical significance was determined using one-way ANOVA (**c**) or two-way ANOVA (**b**) followed by Tukey's multiple comparisons test.*

Materials and methods section:

*"**In vivo inhibitor validation and inflammation-dependent brain delivery studies***

For in vivo inhibitor validation, mice received an intraperitoneal injection of MβCD (100 mg/kg) 1 h before intravenous administration of DiR-labeled siMETTL3-hNVs. In separate experiments, siMETTL3-hNVs were pretreated with RS504393 before injection. Brain fluorescence was quantified by IVIS imaging at 12 h post-injection, followed by ex vivo IVIS imaging of isolated brains."

*"**In vivo inhibitor validation and inflammation-dependent brain delivery studies***

For inflammation-dependent brain delivery studies, mice were assigned to a healthy control group or to neuroinflammatory groups treated with LPS at 0.1, 0.25, 0.5, or 1 mg/kg by intraperitoneal injection once daily for 7 consecutive days. Fluorescently labeled siMETTL3-hNVs were then intravenously administered, and brain accumulation was quantified by IVIS imaging. Brain tissues were collected for CCL2 ELISA."

Results and discussion section:

"MβCD also significantly decreased fluorescence signals in both upper-chamber bEnd.3 cells and lower-chamber BV2 cells (Fig. 3i), and pretreatment of mice with MβCD similarly reduced brain fluorescence intensity after injection of DiR-labeled siMETTL3-hNVs in vivo, with a further reduction

observed when combined with RS504393-pretreated siMETTL3-hNVs (Fig. 3j, k). Together, these findings support that caveolae might be critical mediators in facilitating the BBB crossing of siMETTL3-hNVs, in line with previous studies[43,44].”

“To further evaluate the inflammation dependence of this process, we compared brain accumulation of siMETTL3-hNVs in healthy mice and LPS-induced neuroinflammatory mice with different degrees of inflammation. Brain accumulation was markedly lower in healthy mice and increased with inflammatory severity, in parallel with elevated CCL2 levels in brain tissue (Supplementary Fig. 21). These results are consistent with a CCL2-related chemokine-guided enrichment process.”

Response to Reviewer #3:

Comments:

This manuscript presents a hybrid epigenetic nanomodulator (siMETTL3-hNVs) composed of microglia-derived nanovesicles (NVs) integrated with siMETTL3-loaded synthetic liposomes for neuroinflammation therapy. The concept of m^6A-based epigenetic intervention combined with cytokine decoying is innovative, and the therapeutic effects in neuroinflammation and radiation-induced brain injury models are potentially impactful. However, several key claims regarding CCR2-mediated active targeting, BBB penetration, and formulation properties/stability are not fully supported by the current evidence. Addressing the following concerns is necessary to strengthen rigor and interpretation.

Response: We thank you for the careful and critical evaluation of our study. We particularly appreciate the concerns raised regarding BBB transport, mechanistic interpretation, and the physicochemical characterization of the formulation. In response, we have moderated several statements, performed additional validation experiments, and revised the manuscript extensively to improve rigor and precision.

Comment 1: CCR2-mediated active targeting is not convincingly demonstrated. The manuscript attributes improved brain delivery/targeting to CCR2-mediated chemokine receptor transport, but direct evidence appears insufficient.

Response: We appreciate your concern regarding the strength of this mechanistic claim. We agree that the original manuscript overstated the evidence for CCR2-mediated active targeting and for CCR2-mediated chemokine receptor transport as the direct basis of BBB traversal. In the revised manuscript, we have therefore moderated this interpretation and now describe the CCR2–CCL2 axis primarily as a mechanism that promotes chemotactic enrichment and inflammatory-site homing, rather than as direct evidence for the cellular route of endothelial transport.

To further clarify this point experimentally, siMETTL3-hNVs were pretreated with RS504393, a selective CCR2 antagonist, prior to evaluation. In the bEnd.3–BV2 Transwell model, RS504393 pretreatment significantly reduced the fluorescence signal detected in the lower chamber. Consistently, *in vivo* imaging showed that mice receiving RS504393-pretreated siMETTL3-hNVs exhibited markedly decreased brain fluorescence intensity after intravenous injection (Fig. 3h-k). These findings support the interpretation that the CCR2–CCL2 axis functions as an important chemotactic driver that promotes the brain enrichment of siMETTL3-hNVs under inflammatory conditions.

We therefore next focused on the endothelial transport mechanism itself. Because nanocarriers may cross endothelial barriers through multiple uptake pathways[1,2], we assessed the effects of pathway-specific pharmacological inhibitors. Among these, methyl-β-cyclodextrin (MβCD), an inhibitor of caveolae-associated endocytosis, reduced the transcytosis efficiency of siMETTL3-hNVs by 72.92 ± 0.19%, whereas chlorpromazine (CPZ) and EIPA, inhibitors of clathrin-mediated endocytosis and macropinocytosis, respectively, did not produce significant inhibition (Supplementary

Fig. 20). In addition, MβCD significantly reduced fluorescence signals in both upper-chamber bEnd.3 cells and lower-chamber BV2 cells (Fig. 3i), supporting a major contribution of caveolae-associated transcytosis to endothelial transport *in vitro*. *In vivo*, MβCD pretreatment significantly reduced brain fluorescence intensity after injection of DiR-labeled siMETTL3-hNVs, and this effect was further enhanced when combined with RS504393 pretreatment (Fig. 3j, k). This interpretation is consistent with previous studies reporting caveolae-associated BBB transport of nanocarriers[3,4], which may be facilitated by the abundance of caveolae in endothelial cells[5] as well as by PEGylation[6] and membrane-derived surface components[7] that favor caveolae-associated uptake.

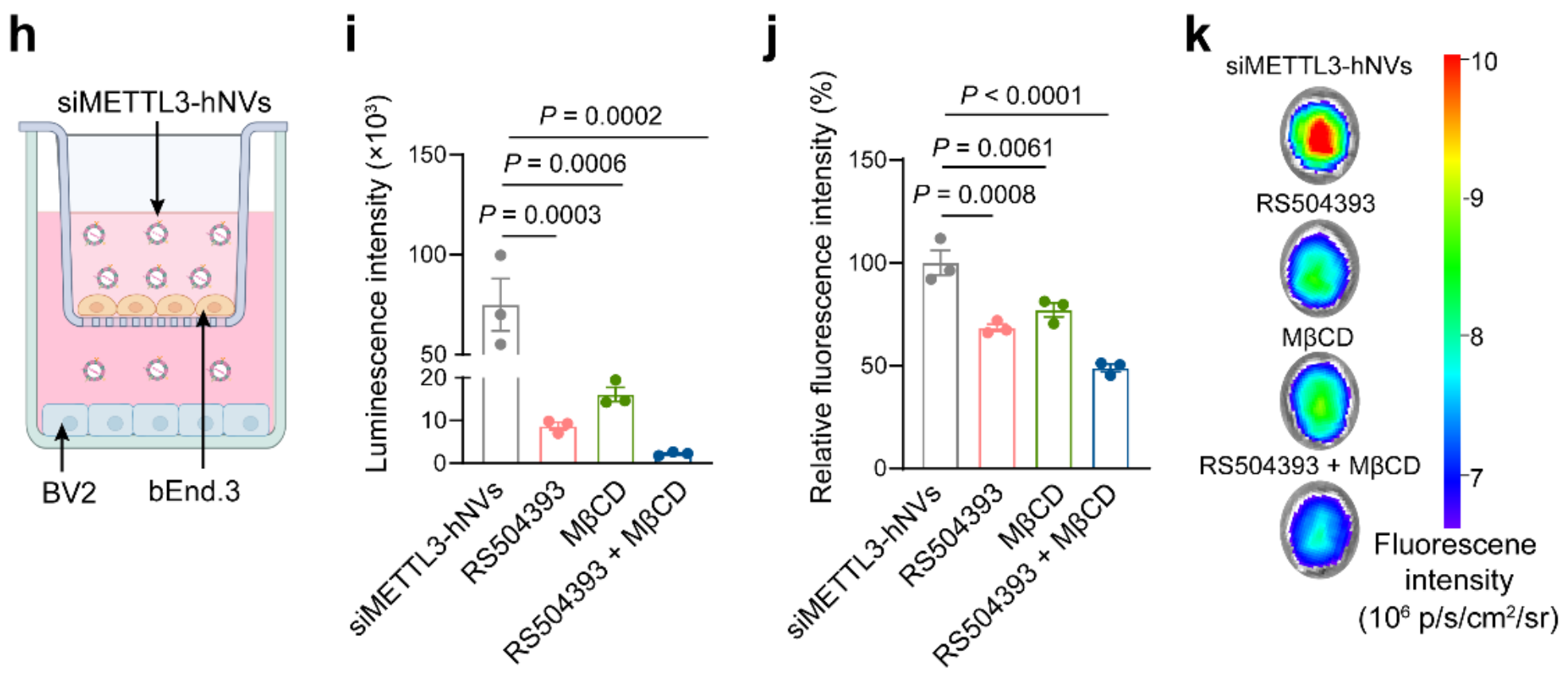

***Fig. 3 | h**, Schematic illustration of the bEnd.3–BV2 Transwell assay for BBB penetration assessment, with BV2 cells seeded in the lower chamber. **i,** Fluorescence intensity of BV2 cells in the lower chamber after treatment with siMETTL3-hNVs, including RS504393-pretreated siMETTL3-hNVs and MβCD-pretreated bEnd.3 cells. **j-k,** Quantification of brain fluorescence intensity (**j**) and representative IVIS images (**k**) after intravenous injection of siMETTL3-hNVs, including mice pretreated with MβCD or receiving RS504393-pretreated siMETTL3-hNVs.*

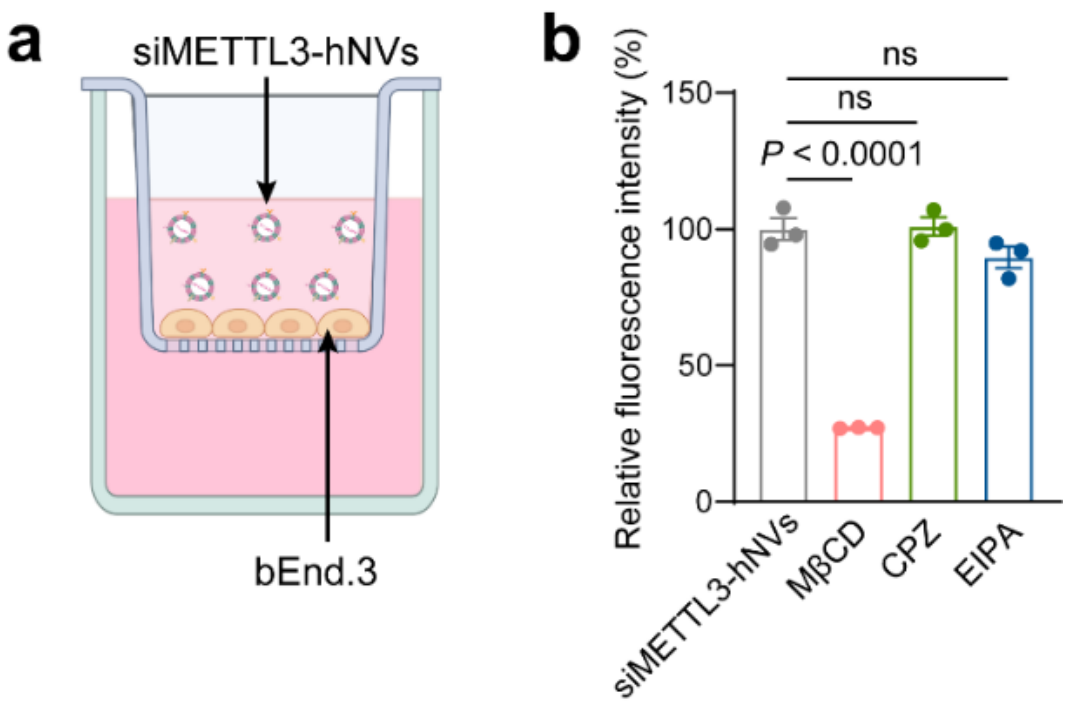

***Supplementary Fig. 20 | a,** Schematic illustration of the bEnd.3 Transwell assay for BBB penetration assessment. **b,** Relative fluorescence intensity of siMETTL3-hNVs in the Transwell system after pretreatment of bEnd.3 cells with different endocytic inhibitors, normalized to the siMETTL3-hNVs group. Data are expressed as mean ± SEM (n = 3). Statistical significance was determined using one-way ANOVA followed by Tukey's multiple comparisons test.*

Reference:

1. G.C. Terstappen, Meyer, A.H., Bell, R.D. & Zhang, W. Strategies for delivering therapeutics across the blood-brain barrier. *Nat. Rev. Drug Discov.* 20, 362-383 (2021)
2. M. Sousa de Almeida, et al. Understanding nanoparticle endocytosis to improve targeting strategies in nanomedicine. *Chem. Soc. Rev.* 50, 5397-5434 (2021)
3. C. Wang, et al. Blood-brain-barrier-crossing lipid nanoparticles for mRNA delivery to the central nervous system. *Nat. Mater.* 24, 1653-1663 (2025)
4. Z. Wang, et al. A sphingolipid-derived paclitaxel nanovesicle enhances efficacy of combination therapies in triple-negative breast cancer and pancreatic cancer. *Nat. Cancer* 6, 1734-1753 (2025)
5. M. Gumbleton, Abulrob, A.G. & Campbell, L. Caveolae: an alternative membrane transport compartment. *Pharm. Res.* 17, 1035-1048 (2000)
6. C. Brandenberger, et al. Quantitative evaluation of cellular uptake and trafficking of plain and polyethylene glycol-coated gold nanoparticles. *Small* 6, 1669-1678 (2010)
7. S.T. Chiang, et al. Biomimetic Nanovesicles Synergize with Short-Term Fasting for Enhanced Chemotherapy of Triple-Negative Breast Cancer. *ACS Nano* 18, 33875-33889 (2024)

Materials and methods section:

*"**In vitro BBB Transwell model and endocytic inhibition assays***

To investigate the mechanism of transendothelial transport, bEnd.3 cells were pretreated with MβCD (4.5 mM), CPZ (5 μg/mL), or EIPA (10 μM) for 30 min at 37 °C, respectively, before addition of siMETTL3-hNVs. To assess the contribution of the CCR2–CCL2 axis, siMETTL3-hNVs were pretreated with RS504393 (10 μM) for 30 min at 37 °C before being applied to the upper chamber. Transendothelial transport was then quantified by flow cytometry analysis of lower-chamber BV2 cells."

*"**In vivo inhibitor validation and inflammation-dependent brain delivery studies***

For in vivo inhibitor validation, mice received an intraperitoneal injection of MβCD (100 mg/kg) 1 h before intravenous administration of DiR-labeled siMETTL3-hNVs. In separate experiments, siMETTL3-hNVs were pretreated with RS504393 before injection. Brain fluorescence was quantified by IVIS imaging at 12 h post-injection, followed by ex vivo IVIS imaging of isolated brains."

Results and discussion section:

"To assess the contribution of the CCR2–CCL2 axis, a bEnd.3-BV2 Transwell model under inflammatory conditions was established as described previously (Supplementary Fig. 18)[40], and we found that pretreatment of siMETTL3-hNVs with RS504393 (a selective CCR2 antagonist) significantly reduced the fluorescence signal in the lower chamber (Fig. 3h, i, Supplementary Fig. 19). Consistently, in vivo imaging showed that mice receiving RS504393-pretreated siMETTL3-hNVs exhibited markedly lower brain fluorescence intensity after intravenous injection (Fig. 3j, k). These results support the interpretation that the CCR2-CCL2 axis acts as a chemotactic cue that promotes inflammatory-site homing and brain enrichment of siMETTL3-hNVs.

BBB translocation depends on the interaction between endothelial cells and nanocarriers and may involve multiple uptake pathways determined by nanoparticle size, shape, surface chemistry,

charge, and morphology[41,42]. In the bEnd.3-BV2 Transwell model, methyl-β-cyclodextrin (MβCD, a caveolae-mediated endocytic inhibitor) reduced the transcytosis efficiency of siMETTL3-hNVs by 72.92 ± 0.19%, whereas chlorpromazine (CPZ, a clathrin-mediated endocytic inhibitor) and 5-(N-ethyl-N-isopropyl)-amiloride (EIPA, a micropinocytosis-mediated endocytic inhibitor) showed no significant effect (Supplementary Fig. 20). This result suggests that endothelial transport of siMETTL3-hNVs may preferentially rely on a caveolae-associated pathway. MβCD also significantly decreased fluorescence signals in both upper-chamber bEnd.3 cells and lower-chamber BV2 cells (Fig. 3i), and pretreatment of mice with MβCD similarly reduced brain fluorescence intensity after injection of DiR-labeled siMETTL3-hNVs in vivo, with a further reduction observed when combined with RS504393-pretreated siMETTL3-hNVs (Fig. 3j, k). Together, these findings support that caveolae might be critical mediators in facilitating the BBB crossing of siMETTL3-hNVs, in line with previous studies[43,44].”

Comment 2: BBB crossing is not fully proven; LPS model likely has compromised BBB integrity. A central claim is that siMETTL3-hNVs “efficiently penetrate the BBB,” yet the LPS-induced acute neuroinflammation model is known to disrupt BBB integrity. Therefore, the observed brain accumulation may reflect passive leakage rather than active BBB transcytosis.

Response: We appreciate your important concern regarding BBB integrity in the LPS-induced neuroinflammatory model. We agree that LPS challenge is associated with increased BBB permeability, and that passive leakage may therefore contribute, at least in part, to brain accumulation in this setting. Consistent with this, Evans blue extravasation was increased in the brains of LPS-treated mice compared with PBS-treated controls, indicating BBB disruption in this model (Supplementary Fig. 15). However, under the same inflammatory background, different formulations still showed markedly different levels of brain accumulation, with siMETTL3-hNVs exhibiting substantially higher fluorescence intensity than Free DiR, siMETTL3@Lipo, and M2-NVs (Fig. 3a-e). These findings indicate that BBB disruption may contribute to brain accumulation, but cannot fully account for the superior brain delivery of siMETTL3-hNVs.

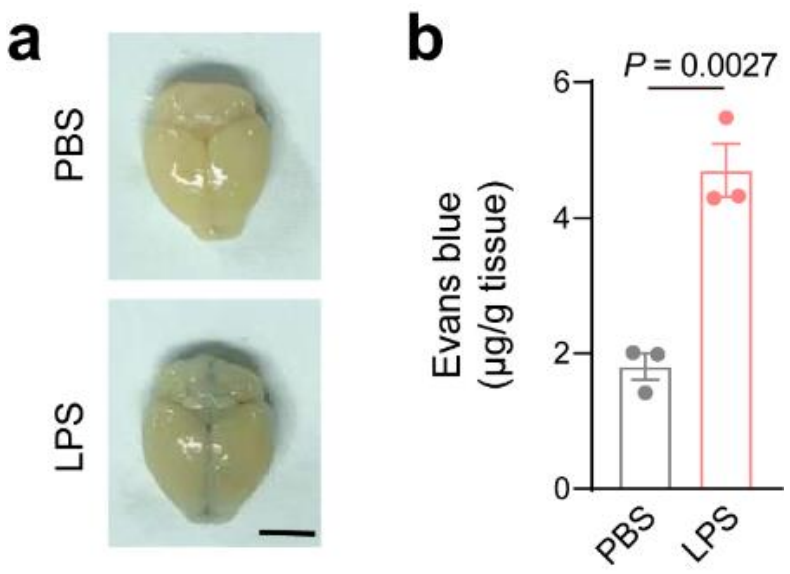

***Supplementary Fig. 15 | a-b,** Representative images (**a**) and Quantification of Evans blue extravasation (**b**) in the brain tissue of PBS- or LPS-injected mice. Scale bars, 0.5 cm. Data are presented as mean ± SEM (n = 3). Statistical significance was determined by unpaired two-tailed t test.*

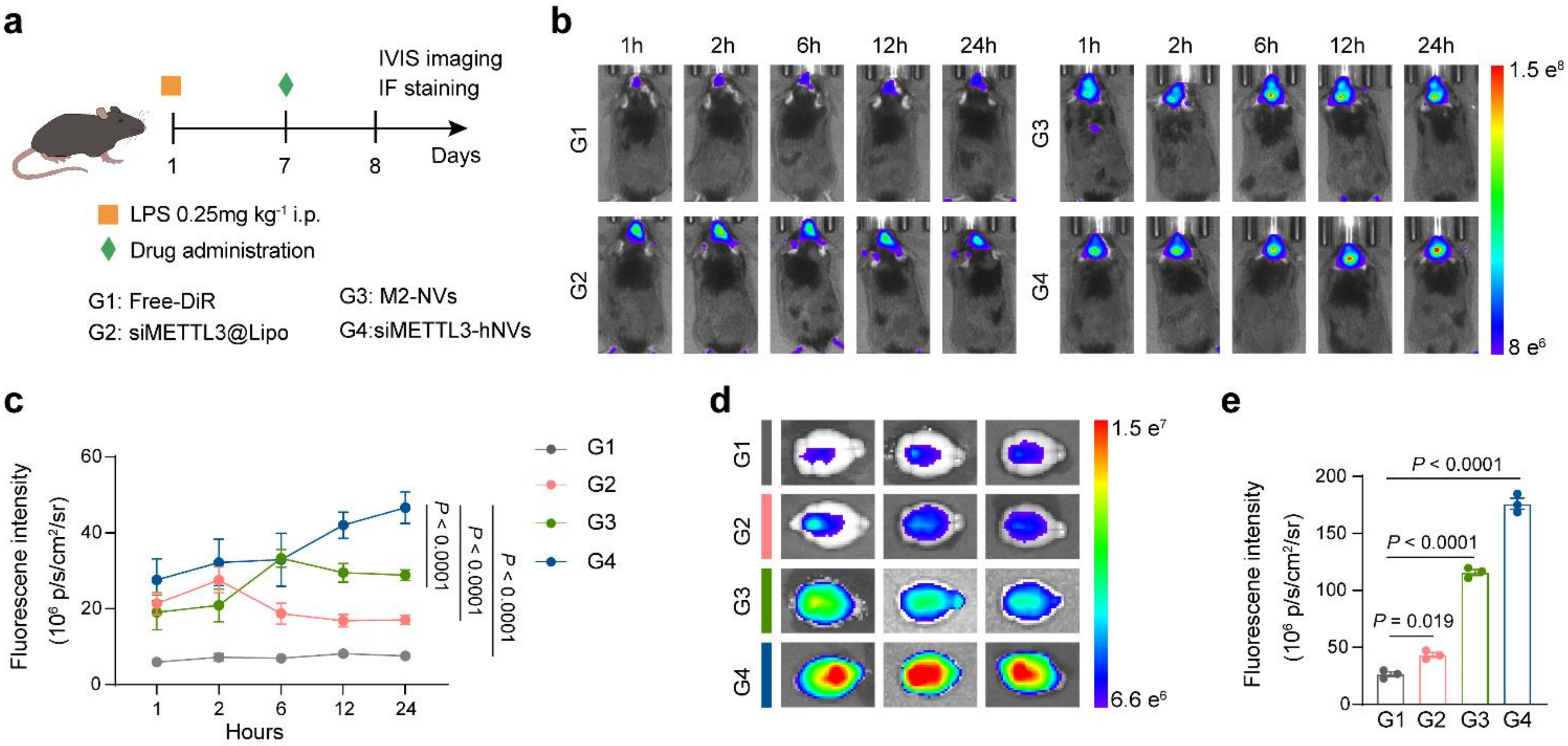

***Fig. 3 | a,** Schematic illustrating the intravenous administration and in vivo imaging timeline in the neuroinflammatory mouse model. **b,** In vivo fluorescence images of mice at different time points after injection of Free DiR, DiR-labeled siMETTL3@Lipo, M2-NVs, or siMETTL3-hNVs. **c,** Quantitative analysis of the brain fluorescence intensity in mice from panel **b** at different time points. **d,** Ex vivo fluorescence images of brains collected 24 h post-injection. **e,** Quantitative analysis of fluorescence intensity in isolated brains.*

Materials and methods section:

*"**Evans blue assay for BBB permeability***

BBB integrity in the LPS-induced neuroinflammatory model was assessed using an Evans blue (EB) extravasation assay. Briefly, mice received intravenous injection of Evans blue solution at the indicated dose, and brains were harvested after circulation for the designated time. For gross imaging, whole brains were photographed immediately after dissection. For quantitative analysis, Evans blue extravasation was determined from brain tissues using the established extraction and fluorescence or absorbance-based readout described in the corresponding figure protocol."

Results and discussion section:

"LPS-induced neuroinflammation is associated with partial BBB disruption[37]. Consistent with this, Evans blue extravasation confirmed increased BBB permeability in our model (Supplementary Fig. 15). Based on this result, we were interested in answering a critical question as to how siMETTL3-hNVs crossed the BBB, namely whether their brain accumulation arose solely from passive leakage or also involved active transport processes. In this regard, the marked differences in brain accumulation among nanoparticle formulations under the same inflammatory conditions (Fig. 3b-e) suggested that passive leakage alone could not fully account for the superior brain delivery of siMETTL3-hNVs."

Comment 3: In Fig. 1c, the nanocarriers show zeta potential close to 0 mV, which often implies limited electrostatic stabilization and potential aggregation during storage. Long-term stability data under

storage conditions should be added.

Response: We thank you for the detailed comment. Although a near-neutral zeta potential may suggest limited electrostatic stabilization, colloidal stability is not determined by surface charge alone[1]. In our formulation, the liposomal component contains DSPE-PEG2000, and hybridization with microglial membrane-derived nanovesicles provides an additional biomimetic interfacial layer, both of which may contribute steric stabilization[2,3]. To directly address this concern, we extended the storage-stability study and monitored siMETTL3-hNVs in PBS and 10% FBS for up to 14 days by DLS. The particles maintained a stable hydrodynamic size distribution without obvious aggregation during the observation period, supporting favorable colloidal stability under storage-relevant conditions (Fig. 2l).

l

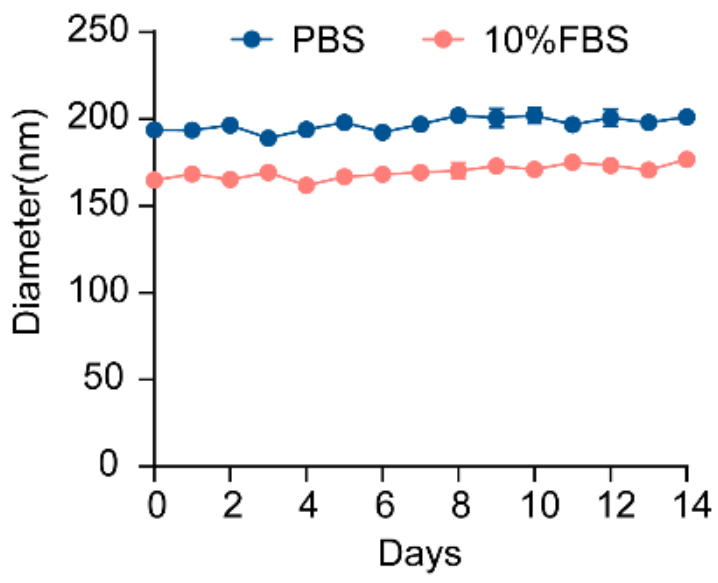

Fig. 2 | l, Stability of siMETTL3-hNVs in PBS or 10% FBS over 14 days, determined by DLS.

Reference:

1. C. Curtis, Toghani, D., Wong, B. & Nance, E. Colloidal stability as a determinant of nanoparticle behavior in the brain. *Colloids Surf. B Biointerfaces* 170, 673-682 (2018)

2. J.S. Suk, Xu, Q., Kim, N., Hanes, J. & Ensign, L.M. PEGylation as a strategy for improving nanoparticle-based drug and gene delivery. *Adv. Drug Deliv. Rev.* 99, 28-51 (2016)

3. J. Schubert & Chanana, M. Coating Matters: Review on Colloidal Stability of Nanoparticles with Biocompatible Coatings in Biological Media, Living Cells and Organisms. *Curr. Med. Chem.* 25, 4553-4586 (2018).

Results and discussion section:

"Additionally, siMETTL3-hNVs exhibited high stability, maintaining structural integrity for at least 14 days in buffer solutions (Fig. 2l)"

Comment 4: The trend in Fig. 1f suggests that increasing the fraction of M2-NVs leads to decreased siRNA encapsulation/stability. This is counterintuitive and needs mechanistic explanation.

Response: We thank you for this insightful comment. The reduced apparent siRNA encapsulation/stability at higher M2-NV fractions is most likely attributable to dilution and partial shielding of the cationic liposomal component, which is the main driver of siRNA complexation in this system[1]. Accordingly, increasing the membrane-vesicle fraction is expected to reduce the effective positive-charge density and thereby weaken siRNA retention to some extent[2]. This trend likely reflects a trade-off between biomimetic membrane incorporation and cargo retention during hybrid

nanoparticle assembly, rather than a contradictory result[3].

Reference:

1. A. Lechanteur, et al. Cationic Liposomes Carrying siRNA: Impact of Lipid Composition on Physicochemical Properties, Cytotoxicity and Endosomal Escape. *Nanomaterials* (Basel, Switzerland) 8 (2018)

2. L. Chen, et al. Recent progress in targeted delivery vectors based on biomimetic nanoparticles. *Signal Transduct. Target. Ther.* 6, 225 (2021)

3. L. Liu, et al. Cell membrane coating integrity affects the internalization mechanism of biomimetic nanoparticles. *Nat. Commun.* 12, 5726 (2021).

Comment 5: Fig. K indicates a pH-responsive release property. However, the formulation description (NVs + liposomes) does not obviously imply pH-triggered release. Please clarify what component(s) confer pH sensitivity and provide compositional details.

Response: We thank you for this valuable comment. The pH-responsive release behavior in our system is primarily attributable to 1,2-dioleoyl-sn-glycero-3-phosphoethanolamine (DOPE) in the liposomal core. DOPE is a well-established fusogenic lipid that destabilizes bilayer structure under acidic conditions and promotes non-bilayer phase transition, thereby facilitating cargo release and endosomal escape[1,2]. In the present study, the liposomal component was composed of DOTAP/DOPE/DSPE-PEG2000/cholesterol at a molar ratio of 20:24:5:1.

Reference:

1. X. Hou, Zaks, T., Langer, R. & Dong, Y. Lipid nanoparticles for mRNA delivery. *Nat. Rev. Mater.* 6, 1078-1094 (2021)

2. Y. Gu, et al. Engineering hybrid nanoparticles for targeted codelivery of triptolide and CYP3A4-siRNA against pulmonary metastatic melanoma. *Sci. Adv.* 11, eadv6990 (2025)

Comment 6: In Fig. 3i–k, siMETTL3-hNVs exhibit higher delivery efficiency than M2-NVs. Since hNVs already incorporate NV components, the manuscript should explain why the hybrid outperforms native M2-NVs (especially if the latter is expected to have strong biological homing).

Response: Many thanks for your important comment. We agree that native M2-NVs retain membrane-derived biofunctions that may support biomimetic recognition[1]. However, the superior performance of siMETTL3-hNVs likely reflects enhanced overall delivery efficiency rather than stronger homing capability alone[2]. Whereas M2-NVs mainly contribute membrane-associated recognition, the hybrid design is expected to improve siRNA loading and formulation robustness, including colloidal stability and circulation-associated stability through the PEGylated lipid component, thereby yielding a more favorable balance between biological functionality and delivery performance[3].

Reference:

1. H.I. Kim, et al. Recent advances in extracellular vesicles for therapeutic cargo delivery. *Exp. Mol. Med.* 56, 836-849 (2024)
2. C. Ducrot, et al. Hybrid extracellular vesicles for drug delivery. *Cancer Lett.* 558, 216107 (2023)
3. M. Piffoux, Silva, A.K.A., Wilhelm, C., Gazeau, F. & Tareste, D. Modification of Extracellular Vesicles by Fusion with Liposomes for the Design of Personalized Biogenic Drug Delivery Systems. *ACS Nano* 12, 6830-6842 (2018).

Results and discussion section:

"These results indicate that incorporation of the M2-NV membrane improves inflamed-brain delivery, whereas the superior performance of siMETTL3-hNVs over native M2-NVs likely reflects the hybrid formulation, which preserves membrane-associated recognition while improving siRNA loading and physicochemical robustness[36]."

Comment 7: If "decoy cytokine neutralization" is a major mechanism, consider quantifying binding/neutralization capacity and its contribution relative to METTL3 knockdown.

Response: Thank you very much for your valuable suggestion. To directly address this point, we quantified the cytokine-scavenging capacity of siMETTL3-hNVs and compared the contribution of the membrane-decoy effect with that of METTL3 knockdown. *In vitro*, 40 μg of siMETTL3-hNVs removed 42.01 ± 6.42 pg of TNF-α, 42.62 ± 5.16 pg of IL-6, and 198.47 ± 11.63 pg of IL-1β, confirming substantial cytokine-scavenging capability. In parallel, M2-NVs reduced TNF-α, IL-6, and IL-1β to a greater extent than siMETTL3@Lipo, with fold differences of 2.8 ± 0.36, 1.87 ± 0.28, and 1.5 ± 0.19, respectively. These findings support a substantial contribution of membrane-mediated cytokine scavenging to the anti-inflammatory activity of siMETTL3-hNVs.

Results and discussion section:

"Notably, 40 μg of siMETTL3-hNVs removed 42.01 ± 6.42 pg of TNF-α, 42.62 ± 5.16 pg of IL-6, and 198.47 ± 11.63 pg of IL-1β, further confirming their substantial cytokine-binding/removal capability."

"Moreover, M2-NVs reduced TNF-α, IL-6, and IL-1β to a greater extent than siMETTL3@Lipo, by 2.8 ± 0.36-fold, 1.87 ± 0.28-fold, and 1.5 ± 0.19-fold, respectively, supporting a substantial contribution of membrane-mediated cytokine scavenging to the overall anti-inflammatory effect."
